# Methods for Evaluation of medical prediction Models, Tests And Biomarkers (MEMTAB) 2018 Symposium: Utrecht, The Netherlands. 2-3 July 2018

**DOI:** 10.1186/s41512-018-0036-3

**Published:** 2018-07-02

## Poster Presentations

### P1 Online interactive tools for exploring and communicating the technically useful and clinically important measures of test accuracy

#### T. R. Fanshawe^1^, M. Power^2^, S. Graziadio^2^, W. Jones^3^, J. M. Ordonez-Mena^1^, A. J. Simpson^3^, A. J. Allen^3^

##### ^1^Nuffield Department of Primary Care Health Sciences, University of Oxford, Oxford, UK; ^2^NIHR Newcastle In Vitro Diagnostics Co-operative Newcastle, Newcastle upon Tyne Hospitals Foundation Trust, Newcastle upon Tyne, UK; ^3^NIHR Newcastle In Vitro Diagnostics Co-operative, Newcastle University, Newcastle upon Tyne, UK

###### **Correspondence:** A. J. Allen

**Background**

Personal experience and a growing body of empirical studies (initiated by Gerd Gigerenzer) show that people generally find it hard to understand statistical measures of test accuracy. Sensitivity and specificity are *technically useful* for comparing assay performance because they are (mathematically at least) independent of study design and disease prevalence. For patients and clinicians, the *clinically important* measures for decision-making are predictive values and their relation to decision thresholds that depend on the personal values (positive and negative) placed on outcomes.

**Objectives**

To help people develop an intuitive understanding of diagnostic accuracy measures and their technical and clinical application.

**Methods**

We introduce the concepts of *technical accuracy* (sensitivity and specificity) and *clinical accuracy* (predictive values) to distinguish between the two main applications of test performance measures, and to be used alongside the concept of *clinical utility*.

We developed two free interactive tools using the RStudio application “Shiny” that allow users to quantitatively and visually explore the effects on technical and clinical accuracy of true and false test results and prevalence.

https://micncltools.shinyapps.io/TestAccuracy/

https://micncltools.shinyapps.io/ClinicalAccuracyAndUtility/

Both tools also show the effects of study sample size on uncertainties in test performance measures. The clinical accuracy tool visualises pre-test and post-test probabilities of disease in relation to clinical decision thresholds for positive and negative test results.

**Results**

Using a point of care test for *Clostridium difficile,* we demonstrate the effect of prevalence and distinct clinical scenarios on the clinical accuracy and utility of the test in the UK NHS.

**Conclusions**

These tools may be useful for developers of clinical tests, authors of test evaluation reports, and clinicians and patients for interpreting and applying test results.

Future developments should include tools to help people quantify their utilities for the outcomes resulting from acting/not acting on test results, and determine what their decision thresholds are.

### P2 Use of PREP model to predict complications in early-onset pre-eclampsia by healthcare professionals and factors influencing their decision-making

#### John Allotey, Ratna Sohanpal, Esther Amaefule, Princee Kalra, Khalid S. Khan, Javier Zamora, Shakila Thangaratinam

##### Queen Mary University of London, London, United Kingdom

###### **Correspondence:** Shakila Thangaratinam

This abstract has been previously published in Category G: E‐Poster Presentations: High Risk Obstetrics and Labour Complications. BJOG: Int J Obstet Gy, 2018; 125: 66-105. DOI:10.1111/1471-0528.7_15132

### P3 External validation of a clinical risk prediction model for predicting early-onset pre-eclampsia using maternal risk factors and mean arterial pressure in early pregnancy

#### Z. T. A. Al-Rubaie^1^, L. M. Askie^2^, H. M. Hudson^2,3^, J. G. Ray^4^, G. Jenkins^5^, S. J. Lord^1,2^

##### ^1^School of Medicine, The University of Notre Dame Australia, Sydney, NSW, Australia; ^2^NHMRC Clinical Trial Centre, University of Sydney, Sydney, NSW, Australia; ^3^Department of Statistics, Macquarie University, Sydney, NSW, Australia; ^4^Departments of Medicine, Health Policy Management and Evaluation, and Obstetrics and Gynecology, St. Michael's Hospital, University of Toronto, Toronto, Ontario, Canada; ^5^Department of Obstetrics, Westmead Hospital, Westmead, NSW, Australia

###### **Correspondence:** ZTA Al-Rubaie

**Background**: Risk prediction models for early-onset pre-eclampsia (requiring delivery <34 weeks’ gestation) may improve maternal and infant health outcomes by identifying women who will benefit from management such as aspirin prophylaxis. Risk models using routinely measured factors are needed in settings where specialised tests are not available. However, few such models have been externally validated.

**Objective:** To assess the performance of the Baschat (2014) [1] risk model that incorporates history of chronic hypertension, diabetes and mean arterial pressure (MAP) to predict early-onset pre-eclampsia in early pregnancy using the Perinatal Antiplatelet Review of International Studies (PARIS) randomised controlled trial dataset.

**Methods**: A retrospective individual-participant data meta-analysis to validate the Baschat model (reported sensitivity 55%/66% at 10%/20% false positive rates (FPRs) respectively, area-under-curve (AUC) 0.83). Trials were eligible if they did not select women based on the presence/absence of high-risk factors; enrolled women <28 weeks’ gestation; and reported model predictors and pre-eclampsia. Women assigned to the control arm were included. Model performance was assessed by estimating sensitivity, specificity, positive (PPV) and negative (NPV) predictive value for predicting early-onset pre-eclampsia at: (i) 0.7% risk threshold to classify low- versus high-risk; and (ii) 10%/20% FPRs as reported in the original publication. The AUC and 95% confidence interval (CI) was calculated. Model calibration was assessed using the Hosmer and Lemeshow goodness-of-fit test and a calibration plot.

**Results**: Three eligible trials included 4510 women. Pre-eclampsia prevalence was 4.9%. For prediction of early-onset pre-eclampsia (n=25, 0.6%), model sensitivity was 28.0% (95% CI 14.3-47.6%), specificity 84.3% (83.2-85.3%), PPV 1.0% (0.5%-2.0%), NPV 99.5% (99.3-99.7%). At 10% and 20% FPRs, sensitivity was 20.0% (8.9-39.1%) and 32.0% (17.2-51.6%) respectively; AUC=0.55 (0.43-0.68), goodness-of-fit *p*=0.86.

**Conclusion**:

Model performance for predicting early-onset pre-eclampsia was poor in this validation population. Determining appropriate risk thresholds for assessment of clinical performance will be important for ongoing model development.

**Reference**

1. Baschat AA, Magder LS, Doyle LE, Atlas RO, Jenkins CB, Blitzer MG. Prediction of preeclampsia utilizing the first trimester screening examination. American Journal of Obstetrics & Gynecology. 2014 Nov 1;211(5):514-e1.

### P4 Selecting biomarkers for evaluation in a prognostic systematic review: a case study from Crohn’s disease

#### L. Archer^1^, D. Boone^2^, S. A. Taylor^2^, S. Halligan^2^, S. Mallett^1^

##### ^1^Institute of Applied Health Sciences, University of Birmingham, Edgbaston, Birmingham B15 2TT, United Kingdom; ^2^Centre for Medical Imaging, UCL, Charles Bell House, 43-45 Foley Street, London, W1W TS

###### **Correspondence:** L. Archer

**Background:** Systematic reviews in prognosis can become unmanageable due to large numbers of predictors, many of which are considered by very few individual studies.

However, a “rule of thumb” excluding predictors only found in few studies can risk excluding clinically important predictors. Methods are needed to select those that are worthwhile for review.

**Objectives:** To describe methods used to select biomarkers for inclusion in a systematic review of prognostic factors for severe Crohn’s disease.

**Methods:** To manage the potentially large number of candidate predictors, we first subdivided the full review into four separate biomarker areas: (1) serological; (2) clinical; (3) genetic and; (4) combinations of tests/biomarkers.

Only biomarkers reported in five or more primary studies were included automatically, with the remainder reviewed by a panel of gastroenterologists to identify those believed to be “promising” despite being reported in few studies. We stipulated *a priori* that only five such “promising” biomarkers would be included across all reviews.

The panel was blinded to how many and which studies had considered each biomarker. Each member ranked their top five across all biomarker areas, with the top scoring biomarkers then being eligible.

**Results:** Overall 169 candidate predictors were identified, 32 were included and 137 were excluded.

The panel selected one additional biomarker each for the serological (CRP) and genetic reviews (FOX03A), while three were selected for the clinical review (severe endoscopic lesions, stricturing disease and response to therapy) in addition to those which were automatically eligible.

**Conclusion:** Our approach eliminated a large volume of biomarkers with insufficient evidence to be clinically useful, and which were not considered promising by our panel.

Reference to expert opinion ensured the review did not exclude important or newer biomarkers while simultaneously minimising inclusion of results that have not been well evaluated in the literature.

### P5 Measurement of cellular oxygenation in critically ill patients receiving red blood cell transfusion

#### M. Baysan^1,2,3^, M. S. Arbous^1,2^, E. G. Mik^4^, N. P. Juffermans^5^, J. G. van der Bom^2,3^

##### ^1^Leiden University Medical Center, Department of Intensive Care, Leiden, the Netherlands; ^2^Leiden University Medical Center, Department of Clinical Epidemiology, Leiden, the Netherlands; ^3^Sanquin Research, Department of Clinical Transfusion Research, Leiden, the Netherlands; ^4^Erasmus Medical Center, Department of Anaesthesiology, Rotterdam, the Netherlands; ^5^Academic Medical Center, Department of Intensive Care, Amsterdam, the Netherlands

###### **Correspondence:** M. Baysan

**Background**: Tissue oxygenation is essential in critically ill patients, but is difficult to measure at the cellular level. The protoporphyrin IX-triple state lifetime technique, measuring mitochondrial oxygenation tension (mitoPO_2_) in vivo, may be a new monitor to measure oxygenation the cellular level. The measurements are obtained through the oxygen-dependent optical properties of protoporphyrin IX.

**Objective:** To determine the feasibility and variability of mitoPO_2_ measurement in critically ill patients with anaemia.

**Methods**: We prospectively included 20 critically ill patients admitted to the Intensive Care of the Leiden University Medical Center with anaemia scheduled to receive a red cell transfusion. We assessed mitoPO_2_ on the anterior chest wall at multiple time points, before and after red cell transfusion. MitoPO_2_ measurements were performed using a COMET monitor (Photonics Healthcare, Utrecht, The Netherlands) on skin primed during 4 hours with an ALA containing patch (Alacare, Photonamic, Wedel, Germany) for induction of mitochondrial PpIX. Reported values are a mean mitoPO_2_ of 5 consecutive measures at each time point.

**Results**: A mitoPO_2_ measurement was obtained in all but 1 participant, most likely due to excessive chlorhexidin at the measurement site. All measurements were above the signal-to-noise ratio of 25, irrespective of severity of critical illness assessed via APACHE IV score (range 49-171). The median and interquartile ranges of mitoPO_2_ before and after transfusion were 66.9 mmHg (IQR 61.5-77.7 mmHg), and 65.8 mmHg (IQR 57.5-87.2) mmHg, respectively. Median within-subject variability was limited during the first 3 hours after transfusion (3.96 (IQR 2.1-11.4)mmHg), but increased considerably after 24 hours (7.9 (IQR 4.3-13.9) mmHg).

**Conclusion**: It is feasible to measure mitochondrial oxygen tension in critically ill patients. The measurements seem to be most reliable in the first 3 hours after patch removal. Interestingly, mitoPO_2_ values in our study population were higher than those previously reported in healthy volunteers.

### P6 The area between curves, a non-parametric method to evaluate a biomarker for patient treatment selection

#### Y. Blangero^1,2^, M. Rabilloud^1,2^, F. Subtil^1,2^

##### ^1^Service de Biostatistique-Bioinformatique, Hospices Civils de Lyon, Lyon, France; ^2^Univ Lyon, Université Lyon 1, CNRS, Laboratoire de Biométrie et Biologie Évolutive UMR 5558, Villeurbanne, France

###### **Correspondence:** Y. Blangero

**Background:** Biological markers able to predict the benefit of a given treatment vs. another one are essential in precision medicine. Classically, a predictive marker is detected through testing a marker- by-treatment interaction in a parametric regression model, and most of the other methods rely on modelling the risk of event occurrence under each treatment arm. All these methods make assumptions that may be difficult to check.

**Objectives:** A simple approach, which does not make any parametric assumption, is proposed to detect and assess the overall predictive ability of a quantitative marker in clinical trials.

**Methods:** This approach is a non-parametric and graphical method that relies on the area between each treatment-arm-specific ROC curve (ABC) as an indicator of the predictive ability of the maker. The approach is justified by the relationship between ROC curves and risk curves, the latter being key tools in assessing predictive markers.

**Results:** A simulation study was conducted to assess the ABC estimation method and compare it with two approaches based on risk modelling: the Total Gain approach (TG) and the interaction approach. The simulations showed that the ABC estimate has a low relative bias and that its confidence interval has a good coverage probability. The mean relative bias in the ABC is at least as low as in the TG in almost all combinations of sample size, ABC, and risk. The power of the ABC estimation method was close to that of the interaction coefficient. The method was applied to PETACC-8 trial data on the use of FOLFOX4 vs. FOLFOX4 + cetuximab in stage III colon adenocarcinoma. It enabled detecting a predictive marker: the DDR2 gene amplification level.

**Conclusion:** The ABC is a simple indicator that may be recommended as a first step in the identification and overall assessment of a predictive marker.

### P7 Development and internal validation of a prognostic model including quantitative fetal fibronectin to predict preterm delivery in symptomatic women (QUIDS study): an IPD meta-analysis

#### M. M. C. Bruijn^1^, E. Schuit^2,6^, R. D. Riley^3^, J. Norrie^4^, R. K. Morris^5^, J. E. Norman^1^, S. J. S. Stock^1^ on behalf of the QUIDS team

##### ^1^Tommy's Centre for Maternal and Fetal Health, MRC Centre for Reproductive Health, University of Edinburgh, UK; ^2^Julius Center for Health Sciences and Primary Care, University Medical Center Utrecht, Utrecht University, Utrecht, the Netherlands; ^3^Centre for Prognosis Research, Research Institute for Primary Care and Health Sciences, Keele University, Staffordshire, UK; ^4^Medical Statistics and Trial Methodology, Usher Institute of Population Health Sciences and Informatics, University of Edinburgh, Edinburgh, UK; ^5^Birmingham Centre for Women's & Newborn's Health, Institute of Metabolism and Systems Research, College of Medical & Dental Sciences, University of Birmingham, Birmingham, UK; ^6^Cochrane Netherlands, University Medical Center Utrecht, Utrecht University, Utrecht, the Netherlands

###### **Correspondence:** MMC Bruijn

**Background**

Accurate prediction of preterm delivery remains notoriously challenging. It would enable targeted interventions and reduce unnecessary hospital admissions and transfers. Quantitative fetal fibronectin (qfFN) is a new bedside test to improve diagnosis of preterm labour.

**Objectives**

To evaluate the accuracy of qfFN to rule out spontaneous preterm delivery within seven days, and to develop and internally validate a decision support tool for the management of symptomatic women.

**Methods**

We performed an IPD meta-analysis of 5 European studies of symptomatic women at 22^+0^-34^+6^ weeks gestation. We used qfFN and clinical risk factors from a pre-defined set of predictors. We used multivariable logistic regression firstly with all predictors, and secondly with backward stepwise selection (threshold of p-value<0.1) to develop a prognostic model to predict preterm delivery within seven days. Multiple imputation was used for predictor values considered missing at random, and non-linear trends allowed for continuous predictors. Clustering and between-study heterogeneity of outcome incidence was taken into account by a separate intercept term per study. The performance of the model was assessed by overall fit (Nagelkerke R^2^), discrimination (AUC). Bootstrap re-sampling techniques were used for internal validation and optimism-adjustment using shrinkage.

**Results**

We included 1783 women, with 139(7.8%) events of preterm delivery within seven days. Table 1 shows the prognostic model before and after variable selection. For the latter, besides qfFN, the model included smoking, ethnicity, nulliparity and multiple pregnancy. After applying a uniform shrinkage factor of 0.92, the model showed an R^2^ of 0.39 and an AUC of 0.89 (95% CI 0.87-0.93).

**Conclusion**

A prognostic model including qfFN and clinical risk factors showed excellent performance in the prediction of preterm delivery. As part of the QUIDS study, the model (including choice of intercept) will be externally validated using data from a prospective cohort study in 26 UK sites.

**Table 1 (abstract P7).** Multivariable models (before and after variable selection) for predicting spontaneous preterm delivery within seven days in symptomatic women

	Model including all variables		Model after variable selection	
	Beta	OR (95% CI)	Beta^a^	OR (95% CI)
Intercept				
Study 1	-7.9		-4.6	
Study 2	-8.5		-5.3	
Study 3	-9.0		-5.7	
Study 4	-8.7		-5.4	
Study 5	-9.3		-6.0	
Quantitative fetal fibronectin (qfFN)^b^				
(qfFN+1)/100)^0.5	2.0	7.6 (5.7 – 10)	1.89	6.6 (4.9 – 8.9)
Age (yr)	0.02	1.0 (0.98 – 1.1)	-	-
BMI (kg/m^2^)	0.02	1.0 (0.96 – 1.1)	-	-
Smoking	-0.66	0.52 (0.24 – 1.1)	-0.67	0.51 (0.24 – 1.08)
Ethnicity				
1 Caucasian	Reference		Reference	
2 South Asian	1.1	2.9 (0.93 – 9.1)	0.94	2.6 (0.84 – 7.9)
3 East Asian	-1.2	0.31 (0.04 – 2.5)	-1.004	0.37 (0.05 – 2.8)
4 African, Caribbean, Middle-East	-0.21	0.81 (0.42 – 1.5)	-0.21	0.81 (0.43 – 1.5)
5 Other	-0.25	0.78 (0.20 – 3.0)	-0.31	0.74 (0.19 – 2.8)
Nulliparity	0.53	1.7 (1.1 – 2.7)	0.37	1.4 (0.92 – 2.2)
Multiple pregnancy	0.85	2.3 (1.4 – 4.1)	0.83	2.3 (1.3 – 3.9)
Previous spontaneous PTD < 34 weeks	0.43	1.5 (0.78 – 1.1)	-	-
Gestational age at assessment (wks)	0.03	1.0 (0.96 – 1.1)	-	-
Nagelkerke R^2^	0.39		0.39	
AUC (95%CI)	0.90 (95% CI 0.88 – 0.93)		0.89 (95% CI 0.87 – 0.93)	

*PTD* preterm delivery < 7 days

^a^Regression coefficients of the predictors in the model were shrunken with a uniform shrinkage factor 0.92. The intercepts were re-estimated after shrinkage of the regression coefficients of the predictors to ensure perfect calibration-in-the-large.

^b^Transformation of continuous variable ‘quantitative fetal fibronectin’ because of non-linearity

### P8 Guidance for deriving and presenting percentage study weights in meta-analysis of test accuracy studies

#### Danielle L. Burke, Joie Ensor, Kym I. E. Snell, Danielle Van Der Windt, Richard D. Riley

##### Keele University, United Kingdom

###### **Correspondence:** Danielle L. Burke

This abstract has been previously published.

Burke DL, Ensor J, Snell KIE, van der Windt D, Riley RD. Guidance for deriving and presenting percentage study weights in meta‐analysis of test accuracy studies. Res Syn Meth. 2018;1–16. https://doi.org/10.1002/jrsm.1283

### P9 Opportunities and challenges of prediction modelling with UK Biobank data

#### F. M. Chappell, J. M. Wardlaw, C. A. Hutchison

##### Neuroimaging Sciences, Centre for Clinical Brain Sciences (CCBS), University of Edinburgh, UK

###### **Correspondence:** F. M. Chappell

**Background**

The UK Biobank dataset (http://www.ukbiobank.ac.uk/about-biobank-uk/) is a resource established by the Wellcome Trust, available to researchers based anywhere. Over 500,000 UK participants contributed extensive health-related data, giving a unique opportunity to investigate predictors of disease.

Data were collected from people aged 40-69, initial assessments were from 2006–2010 and follow-up is ongoing.

**Objectives**

To use early life factors and clinical data to predict stroke and recurrent stroke. To develop a method to identify participants with stroke and date of stroke. Strokes and dates can be self-reported via touchscreen, nurse-led interview, or taken from hospital records. Self-reported stroke without corroboration is not reliable (REF http://journals.plos.org/plosone/article?id=10.1371/journal.pone.0137538), and hospital data is challenging to use.

**Methods**

We compared self-reported strokes, interview-reported strokes, and hospital stroke data and tried to ascertain consistency and accuracy. We estimated the proportion of missing data for key variables.

**Results**

7669 people out of 502,619 reported stroke at initial assessment. This was not confirmed in interview for 1068 participants, while 793 people did say they had had a stroke in interview but not via touchscreen. Only 75% of participants had an interview. Reported dates of stroke have inconsistencies.

The hospital data uses consultant referral as the unit-of-analysis, so a single stroke may have multiple rows. 6548 participants had from 1 to 24 strokes. Admittance dates, needed to work out if a participant has had two strokes or two consultant referrals are incompletely collected, with 23% missing. 846 of the hospital strokes occurred prior to Biobank recruitment but were not self-reported via touchscreen, of these 656 were also not picked up at interview.

Missing data in non-stroke predictors can be extensive. For example, 33% did not report age left full-time education, and 67% are missing cognitive data.

**Conclusion**

UK Biobank is a huge resource, but poses challenges for researchers.

### P10 Surprising results when selecting predictors for a clinical prediction rule

#### Francesca M. Chappell^1^, Fay Crawford^2^, Margaret Horne^3^, on behalf of PODUS CPR Group

##### ^1^Neuroimaging Sciences, Centre for Clinical Brain Sciences (CCBS), University of Edinburgh, UK; ^2^R & D Department, NHS Fife, Dunfermline, UK; ^3^Usher Institute of Population Health Sciences and Informatics, University of Edinburgh, UK

###### **Correspondence:** Francesca M. Chappell

**Background**

We conducted a systematic review and meta-analysis of individual patient data (IPD) on predictors of diabetic foot ulceration. These predictors can be used to develop a clinical prediction rule for health professionals working directly with patients.

**Objectives**

To develop a clinical prediction rule

**Methods**

Using IPD from nine studies (14897 patients), we chose candidate predictors based on (i) clinical plausibility, (ii) availability, (ii) consistency of definition, and (iv) acceptable heterogeneity. From 22 variables, this left six candidates: age, gender, diabetes duration, monofilament testing, pulses testing, and history of ulceration to be used in a two-step meta-analysis (11522 patients). We used a tenth externally held dataset (1489 patients) – not available to the project team – for validation. Predictors were considered validated if the external dataset’s results were consistent with meta-analysis results and they achieved statistical significance.

**Results**

Three predictors were validated in the external dataset: an inability to feel a 10g monofilament, any absent pedal pulse and ulcer history, all binary. Two non-validated predictors were age and diabetes duration – generally considered highly plausible predictors of diabetes complications. They are also continuous variables, which have more statistical power than corresponding categorical variables. We therefore compared logistic regression models using the three validated predictors and all six predictors using discrimination (ROC plots and area under the curve) and calibration plots. The models using the three validated predictors were not lower performing than the models using six predictors.

**Fig. 1 (abstract P10).**
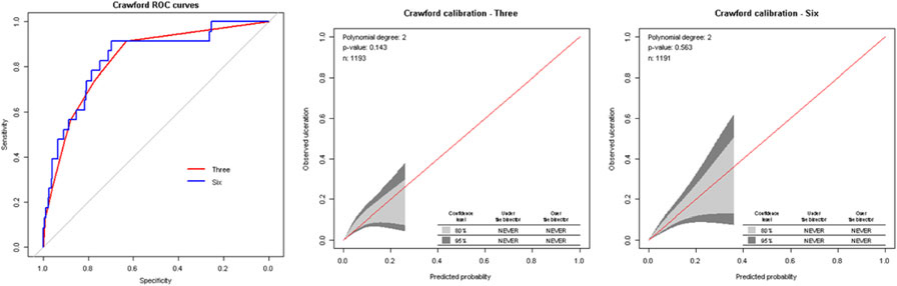
Example results from one study

**Discussion**

The three validated predictors are all foot-specific. The non-validated predictors are all “systemic”. It may be that in the prediction of foot ulcer, data on foot health is more informative than data on the whole patient.

**Conclusion**

Understanding the clinical context and sound statistical methods are important in the selection of predictors.

### P11 Applying Shared Decision Making Platform Improve I-131 Patient Medical Quality

#### J. J. Chen, Y. H. Chang, S. H. Huang, Y. T. Lai

##### Chang Gung Memorial Hospital, Kaohsiung Medical Center, Chang Gung University College of Medicine, Kaohsiung, Taiwan

**Background**

Taiwan patient safety reporting system 2016 annual report states that “communication factor” caused by an event 41% belongs to “between medical staff and patients” communication problems.

**Objectives**

Make use of the simple, easy-to-understand questions on the Shared Decision Making platform to provide a clear and complete explanation of the medical staff's interpretation, cross-comparison, assessment, patient selection, and patient support make decisions, express their willingness to accept and exercise medical consent.

**Methods**

This study will collect the diagnostic statements of all diseases related to thyroid cancer with radioactive iodine 131, purpose of the treatment, the methods of implementation, the possible complications, the success rate and the risk of non-treatment, the treatment alternatives and post-treatment precautions, health status, patient preferences, patient values and so on into the database so that the physician can discuss directly with the patient from the platform to display the relative information needed by the patient to check, which may be appropriate to integrate into the patient Questions and consideration of the problem, to help patients make the most appropriate way to check this.

**Results**

This study is based on the Iodine 131 examination project of Chang Gung Memorial Hospital, Kaohsiung Medical Center and the two major concepts of Evidence-Based Medicine and Shared Decision Making. I-131 Shared Decision Platform architecture is divided into five parts: Patient Search System, Shared Decision Making System, Health Education System, Evidence-Based Medicine System, Data Repository System.

**Conclusion**

To guide patients and their families in structured steps to make important considerations. After discussions between both doctors and patients to reduce their mutual cognitive deficits, they also have three elements of knowledge, communication and respect. They have reached the philosophy of “Quality, Efficiency and service” so as to obtain the best and feasible treatment, protect the patients’ medical interests and enhance the quality of medical care.

### P12 Development of risk prediction models combining routine EHR data for use in colorectal cancer screening referral decisions

#### Jennifer Anne Cooper^1^, Tom Marshall^2^, Ronan Ryan^2^, Nick Parsons^1^, Chris Stinton^1^, Sian Taylor-Phillips^1^

##### ^1^Division of Health Sciences, Warwick Medical School, University of Warwick, Gibbet Hill Road, Coventry CV4 7AL, UK; ^2^Primary Care Clinical Sciences, School of Health and Population Sciences, University of Birmingham, Birmingham, West Midlands B15 2TT, UK

###### **Correspondence:** Jennifer Anne Cooper

**BACKGROUND**: Risk prediction models which incorporate the FOBT with other colorectal cancer risk factors have demonstrated increased sensitivity compared with FOBT alone. EHRs from primary care have a rich level of data and may add a further dimension to risk prediction models. The aim of the study was to determine the availability of GP data for key predictors of colorectal cancer in the screening population and whether we can use this additional information to make more accurate screening referral decisions.

**METHODS**: The Health Improvement Network (THIN) database was used to define a screening population by identifying practices which receive electronic bowel cancer screening programme notifications. A prediction model combining the FOBT with other clinical predictors was developed using Cox Regression and multivariable fractional polynomials with backwards elimination. For internal validation, optimism adjusted performance metrics were determined using bootstrapping and absolute risk predictions were estimated.

**RESULTS**: The screening cohort derived from THIN gave 292,168 patients. The Cox Regression model which included the FOBT result (n=98,303, 1197 colorectal cancer/polyps) had 13 predictors and 2 interactions including; MCV, various symptoms/diagnoses and whether previous polyps had been diagnosed. The optimism adjusted performance metrics gave a; C-statistic of 0.850, c-slope of 0.991, D statistic 2.298 and R^2^ of 0.558. A model investigating negative results only (n = 95,792, 587 colorectal cancer/polyps) included a similar pattern of variables. Performance metrics included a C-statistic of 0.650, C-Slope of 0.944, D statistic 0.836 and R^2^ of 0.144.

**CONCLUSIONS**: This study has shown that a screening cohort can be derived from a primary care database using the electronic bowel cancer screening programme notifications. The prediction models estimate an individual’s absolute risk of colorectal cancer. Additional data could be drawn from primary care onto the Bowel Cancer Screening system using the NHS Spine to contribute to a referral algorithm.

### P13 Performance of the Framingham Risk Score, Pooled Cohort Equations and SCORE for predicting cardiovascular diseases in women with and without a history of hypertensive disorders of pregnancy: an external validation study

#### Veerle Dam^1,2^, N. Charlotte Onland-Moret^1^, W. M. Monique Verschuren^1,3^, Jolanda M. A. Boer^3^, Laura Benschop^2,4^, Arie Franx^5^, Karel G. M. Moons^1^, Eric Boersma^4^, Yvonne T. van der Schouw^1^, on behalf of the CREW-consortium

##### ^1^Julius Center for Health Sciences and Primary Care, University Medical Center Utrecht, Utrecht, the Netherlands; ^2^Netherlands Heart Intstitute, Utrecht, the Netherlands; ^3^National Institute of Public Health and the Environment, Bilthoven, the Netherlands; ^4^Erasmus University Medical Center, Rotterdam, the Netherlands; ^5^University Medical Center Utrecht, Utrecht, the Netherlands

###### **Correspondence:** Veerle Dam

**Background**: Performance of cardiovascular disease (CVD) risk prediction models for the general female population in women with a history of hypertensive disorders of pregnancy (HDP) is not established.

**Objectives**: Assess predictive performance of the Framingham Risk Score (FRS), Pooled Cohort Equations (PCE) and Systematic Coronary Risk Evaluation model (SCORE) in women with a history of HDP, compare these to women without, and determine the effects of models’ recalibration or refitting on predictive performance.

**Methods**: We included 29,751 women of whom 6,302 had a history of HDP and 17,369 had not. Model performance was assessed with calibration (calibration curves, Expected:Observed (E:O) ratios) and discrimination (C-statistics) for the original, recalibrated and refitted FRS, PCE and SCORE models. All three models predict a form of CVD, include classical CVD risk factors as predictors, and have a 10-year prediction horizon.

**Results**: In women with and without HDP, calibration showed an overprediction for FRS and PCE, which decreased after recalibration, whereas the original SCORE model slightly underpredicted, which improved after recalibration. Discrimination was reasonable for all models, C-statistics ranging from 0.70-0.81 (women with HDP) and 0.72-0.74 (women without HDP). Refitting improved this slightly with C-statistics from 0.71-0.83 (women with HDP) and 0.73-0.80 (women without HDP).

**Conclusions**: SCORE performed best in women. FRS and PCE overpredicted risk in women with and without HDP, but improved after recalibrating and refitting the models. No separate model for women with a history of HDP as compared to without a history of HDP, despite their higher baseline risk, seems necessary.

### P14 Personalized dynamic predictions of a binary outcome based on repeated measurements

#### R. Dandis, S. Teerenstra, J. IntHout

##### Department of Health Evidence, Radboud University Medical Center Nijmegen, PO Box 9101, 6500 HB Nijmegen, The Netherlands

**Objective**

To evaluate four approaches used to provide dynamically updates of personalized predictions for a binary outcome based on a repeatedly measured biomarker: likelihood two-stage method (2SMLE), likelihood joint model (JMMLE), Bayesian two-stage method (2SB) and Bayesian joint model (JMB).

**Method**

We applied the four approaches to predict the development of gestational trophoblastic neoplasia (GTN) based on age and repeated measurements of human Chorionic Gonadotropin (hCG), using data from the Dutch Central Registry for hydatidiform moles at the Radboudumc in Nijmegen. We assessed the predictive power using the area under the ROC curves, and obtained dynamically updated predictions for new patients.

**Results**

The JMMLE failed to achieve convergence due to incomplete optimization. The remaining three approaches (2SMLE, 2SB and JMB) gave basically the same estimates, but with slightly higher posterior parameter estimates of the binary submodel of JMB. Using all available data, the three models equivalently showed excellent predictive power. The updated subject-specific predictions for new patients were approximately the same.

**Conclusion**

This study provides comprehensive explanation and R syntax for a toolbox of approaches to obtain updated predictions of a binary outcome based on newly available measurements.

**Keywords**

Longitudinal data, binary outcome, dynamic prediction, two-stage model, joint model, GTN, hCG.

### P15 How accurately do trialists pre-specify sample sizes for test evaluation trials? The experience in NIHR funded trials in the HTA and EME programmes

#### J. Deeks^1,2,3^, L. Archer^1^, V. Cheed^2^, K. Handley^2^, C. A. Hewitt^2^, N. Marchevsky^2^, S. Mehta^2^, L. Quinn^1^, A. Sitch^1,3^, Y. Sun^2^, Y. Takwoingi^1,3^, K. Tryposkiasdis^2^, R. Woolley^2^, Q. Zhou^2,4^, S. Mallett^1,3^

##### ^1^Test Evaluation Research Group, Institute of Applied Health Sciences, University of Birmingham, Edgbaston, Birmingham B15 2TT, United Kingdom; ^2^Birmingham Clinical Trials Unit, Institute of Applied Health Sciences, University of Birmingham, Edgbaston, Birmingham B15 2TT, United Kingdom; ^3^NIHR Birmingham Inflammatory Biomedical Research Centre, University of Birmingham, Edgbaston, Birmingham B15 2TT, United Kingdom; ^4^First Affiliated Hospital, Sun Yat-Sen University, China

###### **Correspondence:** J. Deeks

**Background:** Investigators often struggle to identify appropriate methods for computing sample size for test evaluation studies, and there is often little data available to inform the assumptions made.

**Objective:** To review methods used for sample size calculation for trials of tests and assess the validity of the assumptions made in comparison with the experience of the trial.

**Method:** Final reports from the NIHR Health Technology Assessment (HTA) and Efficacy and Mechanism Evaluation (EME) programmes for all studies evaluating tests were identified. Sample size calculations were identified and classified according to (i) the study outcome and (ii) the method used. Assumed values for key parameters in each sample size calculation were compared with the estimates observed in the studies. Details of any sample size revisions undertaken were identified and reported. All assessments were initially undertaken independently in duplicate and consensus reached through team discussion.

**Results:** 45 reports containing 53 test evaluation studies were identified; 46 studies of accuracy or agreement and 15 of the impact of test use. Sample size calculations were given for 37(80%) of the accuracy and agreement studies using precision based (12), power for differences in paired proportions (10) or independent proportions (9) or compared with a fixed value (3), or other methods (3). 14 (93%) of the impact studies reported calculations of power for differences in independent groups (11), precision (1), or another method (1). 22 (41%) studies reported sample size revisions because of changes in disease prevalence (7), recruitment (12) or other reasons (3). Observed prevalence varied between 4% and 400% of that assumed in sample size calculations.

**Discussion:** Uncertainty in estimates of prevalence is rarely accounted for in sample size calculations, and often requires adjustments to be made during the study. Within study monitoring of prevalence is required during studies of test accuracy and impact.

### P16 Model Based Evaluations of Diagnostic Point –of-Care Tests: Are They Fit for Purpose?

#### K. Breheny^1^, A. J. Sutton^2^, J. Deeks^3,4^

##### ^1^Health Economics Unit, University of Birmingham, Birmingham, B15 2TT, UK; ^2^Leeds Institute of Health Sciences, University of Leeds, Leeds, LS2 9JT, UK; ^3^Test Evaluation Research Group, Institute of Applied Health Sciences, University of Birmingham, Edgbaston, Birmingham B15 2TT, United Kingdom; ^4^NIHR Birmingham Inflammation Biomedical Research Centre, University of Birmingham, Edgbaston, Birmingham B15 2TT, United Kingdom

###### **Correspondence:** J. Deeks

**Background:** Linked evidence or model based evaluations of tests are recommended and frequently used to predict health benefit and assess cost effectiveness. The validity of a linked evidence assessment depends on whether it appropriately models the mechanisms by which tests impact on patient outcomes.

**Objective:** To assess the extent to which model based evaluations of point-of-care tests appropriately account for the effects and impact of changes in timing of tests on patient health and costs.

**Method:** We reviewed model based evaluations of point-of-care tests published between 2004 to 2017 identified by systematic searches of MEDLINE, EMBASE, CINAHL, NHS EED, PsychInfo and HEED. Each model was evaluated for the patient outcomes considered, whether the model estimated the impact of reduced time to diagnosis on health status and costs, and whether societal costs were included.

**Results:** 74 model based evaluations met the inclusion criteria, of which 54 compared point-of-care tests with a slower laboratory counterpart. Of these, only 39% assessed the economic benefits and 37% the health benefits of faster diagnosis. Only 32% assessed the impact on patient health; intermediate outcomes such as rates of correct diagnosis were used instead. 95% of models did incorporate evidence on test accuracy and consider the impact of false positive and false negative results.

**Discussion:** Many model based evaluations fail to capture the effects of point of care tests related to advancing the time to diagnosis and treatment, reduced anxiety and potential cost impact for society and the healthcare system. Neither do they consider the impact of testing on patient health beyond that related to changed accuracy. Ensuring models incorporate the changes in testing pathways associated with early testing, and obtain empirical evidence to populate decision models will lead to model based evaluations that better reflect the impact of point-of-care technologies.

### P17 Selecting candidate predictors for evaluation in a Prognostic study using a Delphi process – a case study from Atrial Fibrillation

#### C. Easter, K. Hemming

##### Institute of Applied Health Sciences, University of Birmingham, Edgbaston, Birmingham B15 2TT, United Kingdom

###### **Correspondence:** C. Easter

**Background:**

Candidate predictors are often selected in a non-systematic way, likely informed by a-priori beliefs as to which are clinically important, although often limited by the availability of existing data and variables recorded. More formal ways of identifying candidate predictors include literature or systematic reviews and expert opinions. However there is no recommended approach for selecting candidate predictors.

**Objective:**

To explore the use of a Delphi process in selecting candidate predictors for use in the development of a prognostic model for Atrial Fibrillation (AF).

**Methods:**

A selection of AF expert healthcare professionals were invited to participate in a Delphi process to select candidate predictors from a group of patient characteristics. This process consisted of completing multiple surveys (rounds) with the aim of gaining consensus amongst the participants for each patient characteristic. Each characteristic was rated independently (using a Likert scale) on how important it is in predicting recurrence of AF. When consensus was reached, the results were analysed and the characteristics were ordered from the most to the least predictive.

**Results:**

Three rounds of the Delphi survey were completed, with the addition of a consensus meeting which concluded in 217 days. In round 1, 57 of 120 characteristics gained consensus (47.5%). In round 2, 35 of 63 characteristics gained consensus (55.6%) and in round 3, 11 of 28 characteristics gained consensus (39.3%). At the consensus meeting the remaining 17 characteristics (14.2%) were discussed and subsequently gained consensus.

**Conclusions:**

Undertaking a Delphi process requires a large amount of time in which to complete and requires commitment from each individual within the expert group to adequately find the most predictive patient characteristics for recurrence of AF. Overall the Delphi process works efficiently when combining a group of expert’s knowledge to identify candidate predictors to use in developing the prognostic model.

### P18 Sample size in test accuracy systematic reviews: A methodological systematic review

#### K. Estrada-Orozco^1^, S. Eiffert^2^, L. Thabane^3^, H. J.Schünemann^3^, R. A. Mustafa^2^

##### ^1^Clinical Research Institute, National University of Colombia; ^2^Division of Nephrology and Hypertension, University of Kansas Medical Center; ^3^Department of Health Research Methods, Evidence and Impact, McMaster University, Hamilton, Ontario, Canada

**Background**: Test accuracy reviews are increasingly published in the literature and their results are used in making clinical and policy decisions. In contrast to clinical trials, there has been little research into the determinants, magnitude, and impact of optimal sample size needed for test accuracy studies.

The objective of our study is to assess the proportion of test accuracy systematic reviews that consider sample size when analyzing and interpreting results.

**Methods:** We conducted a methodological systematic survey of test accuracy systematic reviews published in 2016 and 2017. We are reviewing a 1:1 stratified random sampling of 280 Cochrane vs. non-Cochrane systematic reviews. We will calculate the proportion of systematic reviews discussing sample size in the results, discussion and conclusion of included reviews. For each systematic review, we will calculate the preferred sample size required for accurate results using an equation that integrates the values of prevalence, margin of error and values of sensitivity or specificity (1). We will report the proportion of reviews that meet the minimum sample size.

**Results:** We are in the process of completing this work and we will have the results ready at the time of the presentation.

**Conclusion:** The findings of this study will inform the test accuracy researchers community and clinicians about the current practice of considering sample size as a factor that may affect the quality of the results in both Cochrane and non-Cochrane reviews. We will also explore the frequency that systematic reviews achieve a preferred minimum sample size to appropriately calculate test accuracy. This will work will inform future initiatives to empirically assess the effect of imprecision in test accuracy reviews.

References

1. Hajian-Tilaki K. Sample size estimation in diagnostic test studies of biomedical informatics. Journal of Biomedical Informatics 2014;48 193–204.

### P19 Rationale and Design of UKGRIS – a cluster randomised trial to evaluate the impact of a risk scoring tool on the use of guideline recommended care and impact on clinical outcomes for patients with non-ST-elevation acute coronary syndrome

#### Colin C. Everett^1^, Linda D. Sharples^2^, Catherine Reynolds^1^, Catherine Fernandez^1^, Deborah D. Stocken^1^, Kathryn Carruthers^3^, Harry Hemingway^4 5^, Keith A. A. Fox^3^, Chris P. Gale^6^

##### ^1^Clinical Trials Research Unit, Leeds Institute for Clinical Trials Research, University of Leeds, Leeds, UK; ^2^London School of Hygiene and Tropical Medicine, London, UK; ^3^University of Edinburgh, Edinburgh, UK; ^4^Farr Institute of Health Informatics Research, University College London. UK; ^5^The National Institute for Health Research, Biomedical Research Centre, University College London Hospitals NHS Foundation Trust/University College London, UK; ^6^Leeds Institute of Cardiovascular and Metabolic Medicine, University of Leeds, Leeds, UK

###### **Correspondence:** Colin C. Everett

**Background:** Non-ST Elevation Acute Coronary Syndrome (NSTE-ACS), comprising unstable angina and Non-ST Elevation Myocardial Infarction is the leading cause of emergency hospitalization in Europe and a leading cause of death and disability. Different treatment strategies according to patient risk status are recommended by both National Institute for Health and Care Excellence and the European Society for Cardiology. Although validated risk scores exist to determine a patient’s risk level, no randomised controlled trial has tested the effectiveness of applying an ACS risk stratification tool on the use of guideline recommended treatments and assessed impact on clinical outcomes.

**Objectives:** The UKGRIS (ISRCTN29731761) trial will evaluate the effectiveness of the systematic application of the GRACE risk score on the use of guideline recommended care and major adverse cardiovascular events (MACE).

**Methods:** 2-arm cluster randomised trial. At least 30 UK hospitals randomised 1:1 to GRACE score-based or standard care to determine NSTE-ACS case management. GRACE risk scoring in randomised sites is accompanied by specific Class I guideline recommended processes to follow. (eg pharmacotherapies, invasive coronary strategy, cardiac rehabilitation). Standard care cluster patients are managed as per local policy, but, crucially, GRACE risk scoring is not performed. Endpoints are (co-primary) guideline uptake and composite MACE and (secondary) unscheduled revascularization, duration of inpatient stay and quality of life, all at 12 months. Long term outcomes data will be obtained from routine Electronic Health Records and clinical registries, including Hospital Episode Statistics, and National Institute for Cardiovascular Outcomes Research data. A harmonised international protocol with the AGRIS trial (ACTRN12614000550606) will allow planned individual patient data meta-analysis of long term outcomes.

**Results:** UKGRIS opened March 2017 and is recruiting successfully, with 1075/3000 patients from 30/30 hospitals.

**Conclusion:** The UKGRIS trial will test the clinical impact of the use of a risk prediction model in an urgent care setting.

### P20 Development and validation of pre-test likelihood of coronary artery disease, and external validation of existing risk models

#### Colin C. Everett^1^, James R. Foley^2^, Julia M. Brown^1^, Petra Bijsterveld^2^, David P. Ripley^2^, Sven Plein^2^, John P. Greenwood^2^

##### ^1^Clinical Trials Research Unit, Leeds Institute for Clinical Trials Research, University of Leeds, UK; ^2^Multidisciplinary Cardiovascular Research Centre (MCRC) & Leeds Institute of Cardiovascular and Metabolic Medicine, University of Leeds, Leeds, UK

###### **Correspondence:** Colin C. Everett

**Background**: Pre-test likelihood models recommended in current US guidelines (and 2010 UK guidelines) have been shown to over-estimate the probability of coronary artery disease (CAD). The 2010 UK Guidelines mandating invasive coronary angiography (ICA) for PTL>60% raised concerns of too many non-diagnostic referrals.

**Objectives**: To use data from the CE-MARC (N=752, single centre, recruited 2006-2009) diagnostic accuracy and CE-MARC2 (N=1202, six centres, recruited 2012-2015) intervention trials to develop and validate a multivariable logistic PTL model, and to externally validate the Duke and CAD Consortium models. Both trials recruited patients with stable chest pain suitable for revascularization if required, the latter required Duke Risk PTL of 10-90% and excluded non-cardiac chest pain.

**Methods**: From CE-MARC, 675 patients without prior AMI/ACS (650 had known CAD status by ICA) were used, while 264 CE-MARC2 patients (with known ICA outcome and no prior AMI/ACS) plus 105 anonymised angiography patients selected for Duke PTL <10%,>90% were used. The new model was developed in CE-MARC, and validated in CE-MARC2, while the existing models were externally validated in each dataset. Discrimination (c-statistic) was estimated and calibration assessed by fitting terms for calibration in the large and logistic miscalibration to the existing logistic risk models.

**Results**: In both datasets, all models had similar discrimination (c-statistic range 0.75 to 0.77). The Duke Clinical Risk score over-estimated average prevalence of CAD in these datasets, and generated PTLs too extreme after adjusting for difference in average prevalence. The CAD Consortium models under-estimated prevalence of CAD in both datasets, but generated appropriate PTL estimates once this under-estimation was adjusted for.

**Conclusion**: Despite similar discrimination, the Duke Clinical Risk score was poorly calibrated, even after adjusting for PTL over-estimation. The CAD Consortium models only required prevalence adjustment before being well-calibrated. The new CE-MARC model was well-calibrated, but requires further external validation.

**Table 1 (abstract P20).** Summary of model performance

Model	Discrimination (c-statistic)	Calibration in the large (alpha)	Logistic miscalibration (beta)
CE-MARC: n=675, Mean age 59.4, 61.8% male, Median CAD PTL by [Duke Clinical/CAD Consortium Basic/Clinical] = [56.2%, 18.3%, 17.6%]. CAD detected 235/650 (36.2%)			
CE-MARC (2016)	0.779 (0.742, 0.814)	NA	NA
Duke Clinical Risk Score (1993)	0.763 (0.725, 0.800)	-1.108 (-1.305, 0.911); P<0.001	-0.298 (-0.416, -0.180); P<0.001
CAD Consortium (2012) Basic	0.770 (0.733, 0.806)	0.713 (0.532, 0.893); P<0.001	-0.015 (-0.131, 0.101); P=0.803
CAD Consortium (2012) Clinical	0.762 (0.725, 0.7995)	0.822 (0.639, 1.005); P<0.001	-0.051 (-0.159, 0.057); P=0.354
CE-MARC2+ (n=369, Mean age 59.6, 43.4% male, Median CAD PTL by [Duke Clinical/CAD Consortium Basic/Clinical] = [67.4%, 16.8%, 14.8%]. CAD detected 158/369 (42.8%)			
CE-MARC (2016)	0.777 (0.731, 0.824)	0.045 (-0.190, 0.280); P=0.709	0.028 (-0.214, 0.269); P=0.823
Duke Clinical Risk Score (1993)	0.752 (0.704, 0.801)	-1.016 (-1.265, -0.766); P<0.001	-0.207 (-0.363, -0.050); P=0.010
CAD Consortium (2012) Basic Model	0.755 (0.706, 0.803)	0.738 (0.507, 0.969); P<0.001	-0.007 (-0.182, 0.169); P=0.940
CAD Consortium (2012) Clinical	0.752 (0.703, 0.800)	0.866 (0.629, 1.103); P<0.001	-0.054 (-0.121, 0.105); P=0.507

### P21 Developing risk models for multicenter data using standard logistic regression produced suboptimal predictions: a simulation study

#### N. Falconieri^1^, B. Van Calster^1,2^, D. Timmerman^1,3^, L. Wynants^1^

##### ^1^KU Leuven, Department of Development and Regeneration, Herestraat 49 box 805, Leuven, Belgium; ^2^Department of Biomedical Data Sciences, Leiden University Medical Center (LUMC), Leiden, Netherlands; ^3^Department of Obstetrics and Gynecology, University Hospitals Leuven, Leuven, Belgium

###### **Correspondence:** N. Falconieri

The objective of this research is to evaluate the predictive performance of regression methods to develop clinical risk prediction models using multicenter data, and provide guidelines for practice.

To this end, we compared the predictive performance of standard logistic regression, generalized estimating equations, random intercepts logistic regression and fixed effects logistic regression. First, we presented a case study on the diagnosis of ovarian cancer using data from the International Ovarian Tumor Analysis group (IOTA). Subsequently, a simulation study investigated the performance of the different models as a function of the amount of clustering, development sample size, distribution of center-specific intercepts, the presence of a center-predictor interaction and the presence of a dependency between center effects and predictors. During validation, both new patients from centers in the development dataset and from new centers were included.

The results showed that sufficiently large sample sizes lead to calibrated predictions under conditional models and miscalibrated predictions under marginal models. Small sample sizes led to overfitting and unreliable predictions. This miscalibration was worse with more heavily clustered data. Calibration of random intercepts logistic regression was better than that of standard logistic regression even when center-specific intercepts were not normally distributed, a center-predictor interaction was present, center effects and predictors were dependent, or when the model was applied in a new center.

In conclusion, to make reliable predictions in a specific center, we recommend random intercepts logistic regression.

### P22 Was evidence on test accuracy from secondary care enough to approve faecal calprotectin testing for primary care use?

#### Karoline Freeman^1^, Brian Willis^2^, Hannah Fraser^1^, Sian Taylor-Phillips^1^, Aileen Clarke^1^

##### ^1^Warwick Medical School, University of Warwick, Coventry, UK; ^2^Institute of Applied Health Research, University of Birmingham, Birmingham, UK

###### **Correspondence:** Karoline Freeman

**Introduction:** Decisions about test availability for patient care are often based on limited evidence. Faecal calprotectin (FC) testing has been approved by NICE for the differential diagnosis of inflammatory bowel disease and irritable bowel syndrome in UK primary care in adults with unexplained abdominal complaints. The decision was based solely on evidence from secondary care. However, transferability of test accuracy estimates between settings cannot be assumed when patient populations differ between settings. We aimed to reassess the evidence against a primary care pathway with FC testing to evaluate what we know about test accuracy of FC testing in primary care.

**Methods:** We updated the previous test accuracy review [1] of FC testing with colonoscopy as the reference standard. Meta-analyses in R version 3.4.1 explored heterogeneity.

**Results:** Thirty-eight studies were eligible including five from primary care. The studies’ patient populations, however, resembled a continuum from primary to secondary care. None of the studies sufficiently addressed the research question. Primary care studies either defined the target disease broader than the intended IBD group or did not use the preferred reference standard. The studies were highly heterogeneous in terms of tests and clinical question frequently offering more than one 2x2 diagnostic table for different tests and different clinical questions. Meta-analysing outcomes and investigating setting as a covariate was not feasible as this would have required expressing a preference for a test and clinical question and disregarding others. Separate exploration of test type and clinical question by meta-regression showed that neither can be assumed to be generic.

**Discussion:** We are lacking evidence to ascertain the assumed test performance of FC testing in primary care. Alternative approaches to simply categorising settings into primary and secondary care are needed to assess studies for their plausibility to reflect the performance of FC testing in primary care.

**Reference**

1. Waugh N, Cummins E, Royle P, Kandala NB, Shyangdan D, Arasaradnam R*, et al.* Faecal calprotectin testing for differentiating amongst inflammatory and non-inflammatory bowel diseases: systematic review and economic evaluation*. Health Technology Assessment (Winchester, England)*. 2013;17(55):xv-xix, 1-211.

### P23 Agreeing on agreement analysis before conduct – five questions you should ask yourself and discuss with your statistician

#### O. Gerke^1^, S. Möller^2^, B. Debrabant^3^, U. Halekoh^3^; Odense Agreement Working Group

##### ^1^Department of Nuclear Medicine, Odense University Hospital, Odense, Denmark; ^2^Odense Patient Data Exploratory Network, Odense University Hospital, Odense, Denmark; ^3^Department of Public Health, Epidemiology, Biostatistics, and Biodemography, University of Southern Denmark, Odense, Denmark

###### **Correspondence:** O. Gerke

**Background:** Guidelines for Reporting Reliability and Agreement Studies (GRRAS) were established in 2011. Studies of agreement and/or reliability are in our experience more often than not part of larger diagnostic accuracy studies, clinical trials, or epidemiological studies in which agreement and/or reliability are reported as quality control by using data of the main study. Unfortunately, the planning of such minor studies regularly fails to precede its conduct and/or researchers are unfamiliar with central concepts of agreement and reliability.

**Objectives:** To propose 5 questions to be addressed in the planning phase from a statistical point of view in order to secure an appropriate analysis plan for an agreement and/or reliability study that actually illuminates what it is supposed to illuminate.

**Methods:** We gathered examples from our consultancy experience and derived an overview sheet characterizing agreement and/or reliability studies. Then, we identified 5 central questions to fine-tune the statistical analysis and related these to respective items of GRRAS.

**Results:** (1) Do you want to investigate interrater/intrarater agreement or reliability? {Item 1}; (2) Who represents the rater population of interest? {Item 4}; (3) Which factors shall your model be accounting for? {Item 6}; (4) Which indices for agreement and/or reliability are you aiming for? {Item 13}; (5) What is your statistical analysis plan? {Item 10}.

**Conclusion:** GRRAS state explicitly that *“Researchers should clearly state a priori their assumptions, why a certain approach was chosen, and what was intended to be demonstrated.”* However, this is in our experience alarmingly often not the case. GRRAS have proven to be most helpful, but consulting on agreement and reliability studies resemble a continuous awareness campaign. We hope that our 5 supplementary questions to GRRAS help improving the planning of such studies which, in turn, are then more focused, more appropriate, and more easily reported by using GRRAS.

### P24 Study designs for response evaluation with PET/CT – a systematic review

#### K. Ehlers^1,2^, O. Gerke^1^, E. Motschall^3^, P. F. Høilund-Carlsen^1,2^, W. Vach^4^

##### ^1^Department of Nuclear Medicine, Odense University Hospital, Odense, Denmark; ^2^Department of Clinical Research, University of Southern Denmark, Odense, Denmark; ^3^Institute for Medical Biometry and Statistics, University of Freiburg, Freiburg, Germany; ^4^Department of Orthopaedics and Traumatology, University Hospital Basel, Basel, Switzerland

###### **Correspondence:** O. Gerke

**Background:** Response evaluation with PET/CT has potential in personalizing cancer treatment and evaluating treatment response. PET/CT is a powerful assessor due to its ability to differentiate between anatomical and physiological response, but designing clinical studies is challenging; there is no consensus on which response criteria to follow, which time points to choose for scans, and how to establish rules for differentiating between responders and non-responders.

**Objectives:** To create an overview of study designs used for response evaluation with PET/CT and basic methodological characteristics.

**Methods:** PubMed/MEDLINE and Web of Science were systematically searched for original articles on response evaluation in cancer, published in 2015 and employing at least one baseline and one post-baseline PET/CT scan.

**Results:** We could identify 124 studies, 61% being prospective and the remaining 39% retrospective. Fifty-two percent were prognostic, 28% accuracy studies, and 10% a mix of these. Nor RCT could be found. Most studies used (one) fixed time points for imaging and evaluated one predefined rule for response evaluation. The median sample size was 39. Seventeen medical areas were represented in the study, the largest group being lymphomas (N=20, 16%); PET time points varied hugely in this group.

**Conclusion:** From a methodological point of view, we expected to see descriptive, accuracy, and prognostic studies as well as RCTs of which there were none. Overwhelming 90% of the studies were either accuracy and/or prognostic studies. Overall comparative elements like inclusion of alternative tracers or modalities, several follow-up time points, variation in parameters (SUVmax, SUVmean, SUVpaek, SUL, MTV) and summary measures (absolute or relative) were less frequent than desirable. There is a noticeable gap between the kinds of studies currently used in response evaluation with PET/CT on the one hand and those we would expect for establishing efficient response evaluation schemes based on PET/CT on the other hand.

### P25 Misrepresentation and overinterpretation in evaluations of biomarkers in ovarian cancer

#### M. Ghannad^1,2^, M. Olsen^1^, I. Boutron^2^, P. P. M. Bossuyt^1^

##### ^1^AMC, UVA, Clinical Epidemiology, Amsterdam, the Netherlands; ^2^Centre de Recherche Épidémiologie et Statistique Sorbonne Paris Cité (CRESS-UMR1153), Université Paris Descartes, France

###### **Correspondence:** M. Ghannad

**Background**: Research in cancer biomarkers has expanded in recent years. However, despite the large number of publications, very few biomarkers have been successfully implemented in the clinic. Biomarker discovery studies may suffer from weak study designs, and incomplete or biased reporting, rendering them vulnerable to exaggerated interpretation of biomarker performance. Spin is a way of reporting, conscious or unconscious, that makes the study findings appear more favourable than results justify.

**Objectives**: We aimed to (1) document and classify spin (i.e., misrepresentation and overinterpretation of study findings exaggerating the performance of the biomarker), and (2) facilitators of spin (i.e., practices that facilitate overinterpretation of results), in recent clinical studies evaluating the performance of biomarkers in ovarian cancer.

**Methods**: We searched PubMed systematically for all evaluations of biomarkers in ovarian cancer published in 2015. Studies eligible for inclusion reported the clinical performance of prognostic, predictive, or diagnostic biomarkers. Reviews, animal studies, and cell line studies were excluded. All studies were independently screened by two reviewers.

**Results**: In total, 1026 citations were retrieved by our search strategy; 326 studies met all eligibility criteria, of which the first 200 studies, when ranked according to publication date, were included in our analysis. One-third (60; 30%) of studies were free of spin, one-third (65; 32.5%) contained one type of spin, and another third (75; 38%) contained two or more forms of spin in the article. Spin was classified into two categories: (1) misrepresentation, (2) misinterpretation. The most frequent forms of spin identified (Table 1) were: (1) other purposes of biomarker claimed not investigated (65; 32.5%); (2) mismatch between intended aim and conclusion (57; 28.5%); and (3) incorrect presentation of results (40; 20%). Frequently observed facilitators of spin (Table 2) were: (1) not stating sample size calculations (200; 100%); (2) not mentioning potential harms (200; 100%); and (3) not pre-specifying a positivity threshold for a continuous biomarker (84 of 164 studies; 51.2%);

**Conclusion:** Reports of studies evaluating the clinical performance of biomarkers in ovarian cancer frequently have spin. Misinterpretation and misrepresentation of biomarker performance may account for a considerable amount of waste in the biomarker discovery process.

**Table 1 (abstract P25).** Actual forms of spin in clinical studies evaluating performance of biomarkers in ovarian cancer with examples

Category of spin	Type of spin	Criteria	Spin frequency, n = 200 n (%) [95% CI]
Misrepresentation a. 1	Incorrect presentation of results in the abstract or main text conclusion	Abstract conclusion OR main text conclusion for BM’s clinical performance is not in accordance with or is stronger than results justify. Actual spin if all the following: a. Exaggerating the performance of the BM in the conclusion despite low performance measures reported in the results; b. Claiming effect of the BM despite statistically non-significant results; c. Claiming effect despite not providing imprecision or statistical test (confidence interval or *P* values) between different biomarker models tested or patient groups (subgroups);	40 (20% [15% - 26%]) Frequency in abstract conclusion: 14 (7% [4% - 12%]) Frequency in main text conclusion: 37 (18.5% [14% - 25%])
a. 2	Mismatch between results reported in abstract and main text	Results reported in the abstract is not in accordance with results reported in main text. Actual spin if all the following: a. Results reported in the abstract contains statement in which statistical significance is claimed, despite not providing imprecision or test of significant (CI or p-values) in results reported in the main text; b. Selective reporting of statistically significant outcomes in the abstract compared to the results reported in the main text; c. Results reported in the abstract that do not match results provided in the main text;	33 (16.5% [12% - 23%])
a. 3	Mismatch between results reported and the title	The title contains wording misrepresenting BM’s clinical performance compared to results in the main text;	11 (5.5% [3% - 10%])
Misinterpretation a. 4	Other purposes of biomarker claimed not pre-specified and/or investigated	Abstract conclusion OR main text conclusion contains statement suggesting BM purposes not pre-specified and/or investigated.	Total: 65 (32.5% [26% - 40%]) Frequency in abstract conclusion: 36 (20.5% [13% - 24%]) Frequency in main text conclusion: 60 (30% [24% - 37%])
a. 5	Mismatch between intended aim and abstract or main text conclusion	Abstract conclusion OR main text conclusion for BM’s clinical performance is stronger than study design. Actual spin if all the following: a. The main text conclusion contains statement in which BM utility is claimed despite not evaluating clinical effectiveness (i.e. useful); b. The main text conclusion contains statement in which BM performance improvement is claimed despite not evaluating incremental measures (i.e. improve); c. The main text conclusion contains statement that uses causal language for BM(s) being assessed despite the use of a nonrandomized design;	Total: 57 (28.5% [23% - 35%]) Frequency in abstract conclusion: 41 (20.5% [15% - 27%]) Frequency in main text conclusion: 31 (15.5% [11% - 21%])
a. 6	Other benefits of BM claimed not pre-specified and/or investigated	The main text conclusion contains statement claiming BM benefits not pre-specified and/or investigated.	10 (5% [3% - 9%])
a. 7	Extrapolation from study participants to a larger or a different population	The main text conclusion contains statement that extrapolates BM’s clinical performance to a larger or a different population, not supported by recruited subjects.	10 (5% [3% - 9%])

^*^ All results presented in abstract and main text, excluding supplementary material.

^**^Abbreviations: BM, biomarker; HR, hazard ratio; OS, overall survival; PFS, progression-free survival.

**Table 2 (abstract P25).** Facilitators of spin in clinical studies evaluating performance of biomarkers in ovarian cancer

Potential facilitators of spin	Spin frequency, n= 200 n (%) [95% CI]
Not stating sample size calculations	200 (100% [98% - 100%])
Not mentioning potential harms	200 (100% [98% - 100%])
Not pre-specifying a positivity threshold for continuous biomarker	84/164* (51.2% [43% - 59%])
Incomplete or not reporting imprecision or statistical test for data shown	26 (13% [9% - 19%])
Study objective not reported or unclear	24 (12% [8% - 18%])

^*^ 164 articles included evaluation of continuous biomarkers

### P26 The role of local guidelines in understanding care pathways and their variability in UK: a case study

#### S. Graziadio^1^, E. Kampouraki^2^, A. Jesuthasan^2^, B. Messer^3^, J. Allen^2^, W. Jones^2^, J. Simpson^2^, M. Power^1^

##### ^1^NIHR Newcastle In Vitro Diagnostics Co-operative, Newcastle upon Tyne Hospitals Foundation Trust, UK; ^2^NIHR Newcastle In Vitro Diagnostics Co-operative, Newcastle University, UK; ^3^Newcastle upon Tyne Hospitals Foundation Trust, UK

**Background**

Adoption of a clinical test into NHS practice requires evidence on its accuracy, usability, clinical utility, affordability and cost-effectiveness. This entails evaluating the changes in clinical and economic outcomes to the care pathway (ie journey that patients make through the healthcare system) resulting from potential adoption. Methods for evaluating utility and cost-effectiveness are well established, and new methods are being developed. However, little research has been done to evaluate the processes that provide the data for these evaluations, ie care pathway analysis, modelling, implementation and evaluation.

**Objectives**

We aim to identify new methodologies for care pathway analysis. In this instance we evaluated, through a case study, the utility of collecting and analysing NHS local guidelines.

**Methods**

The pathways used to recognise patients with suspected sepsis were compared between 14 Trusts and with the NICE guidelines. Recommended symptoms and thresholds were identified and categorized.

**Results**

The recommended physiological signs to consider for early identification of patients with suspected sepsis were consistent across sites and with NICE guidelines, but thresholds were different. The number of steps that would lead to identifying patients for review and initiation of the Sepsis 6 bundle was also different across Trusts. This leads to a different number of patients treated for sepsis across UK independently of the true disease prevalence.

**Conclusion**

The analysis of local guidelines:

1. clarified the physiological signs that influence the clinical decision making during the pathway;
2. supported the development of a high-level map common to the majority of Trusts, first step for care pathway modelling;
3. provided insights about variability in patient care across different UK.

In conjunction with data from the Hospital Episode Statistics, the algorithms described in the guidelines can be powerful tools to calculate the ranges for prevalence and distributions of outcomes associated to specific diseases.

### P27 Development, evaluation and external validation of clinical prediction models to identify adult patients (aged 18 – 50) with type 1 diabetes requiring early insulin therapy

#### Anita L. Grubb^1^, Kashyap Patel^1^, Richard A. Oram^1,2^, Anita V. Hill^1^, Catherine Angwin^1^, Tim J. McDonald^1,2^, Mike N. Weedon^1^, Andrew T. Hattersley^1,2^, Katharine R. Owen^3,4^, Beverley M. Shields^1^, Angus G. Jones^1,2^

##### ^1^National Institute for Health Research Exeter Clinical Research Facility, University of Exeter Medical School, Exeter, UK; ^2^Royal Devon and Exeter NHS Foundation Trust, Exeter; ^3^Oxford Centre for Diabetes Endocrinology and Metabolism, University of Oxford, Churchill Hospital, Oxford, UK; ^4^Oxford NIHR Biomedical Centre, Churchill Hospital, Oxford, UK

###### **Correspondence:** Anita L. Grubb

**Background**

Correctly determining diabetes subtype is important to ensure optimal treatment and education, but is often difficult, particularly in young adults, where misclassification is common. We aimed to develop and externally validate two clinical prediction models combining clinical features and GAD autoantibodies (marker of Type 1 immune process) to identify patients with type 1 diabetes (T1D), requiring early insulin therapy.

**Methods**

We used 1,352 participants diagnosed with diabetes aged 18-50 years from Exeter cross-sectional cohorts to develop two logistic regression models predicting requirement for early insulin therapy:1) using clinical features (age at diagnosis and BMI); 2) clinical features and GAD (n=1036 with GAD available). Discrimination and calibration performance of the models were estimated using internal bootstrap validation. External validation of the models was performed in 701 and 657 participants taken from the Young Diabetes in Oxford study.

**Results**

Prevalence of T1D was 13.24% & 13.51% in the development samples versus 19.12% & 18.57% in the external validation data.

The model with clinical features alone was highly discriminative at development (c-statistic 0.90 [0.88, 0.93]); internal bootstrap validation showed a small optimism (0.0006). Adding GAD improved development model (c-statistic 0.96 [0.95-0.97]); internal bootstrap validation showed a small optimism (0.0009). In the external validation, both models still showed excellent discrimination (clinical features c-statistic 0.86 [0.82, 0.89]; clinical features + GAD c-statistic 0.92 [0.89, 0.95]).

Hosmer-Lemeshow test for calibration was non-significant at development in both models (p=0.95 & 0.39). However, there was evidence of overall miscalibration at external validation (p = 0.004 & 0.007), with both models over-estimating in the higher risk groups (Fig 1).

**Conclusion**

We developed highly discriminatory models for classifying patients requiring early insulin therapy. Addition of GAD improved the model performance. Further investigation is required to identify the reason for the miscalibrations at the highest probabilities in external validation.

**Fig. 1 (abstract P27).**
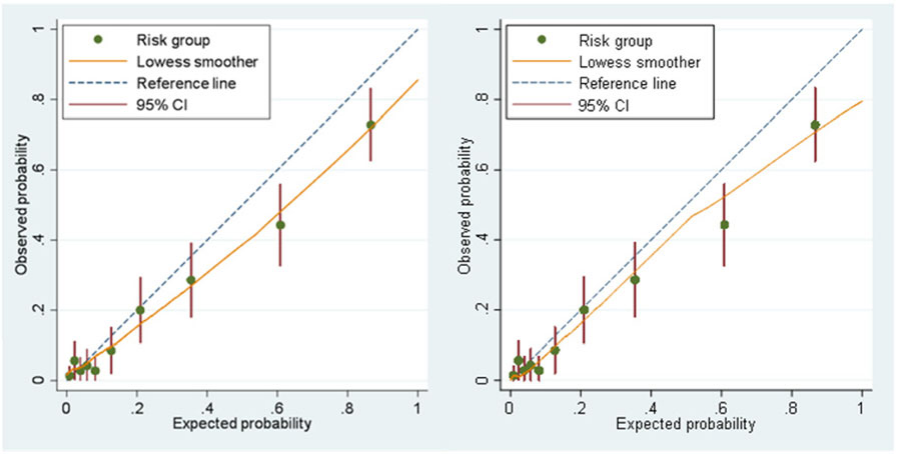
External validation calibration plots for the clinical features model (left) and clinical features + GAD model (right)

### P28 An online platform for clinical risk prediction models: Evidencio

#### T. A. Hueting, R. G. Pleijhuis, E. Verbeek, R. J. Mentink

##### Evidencio, Haaksbergen, the Netherlands

###### **Correspondence:** T. A. Hueting

**Background**: Clinical prediction models (CPMs) are widely used in healthcare to support medical decision-making based on patient-specific characteristics. Over the past decade, there has been an exponential growth of peer-reviewed articles regarding the development of CPMs. However, the majority of these models lack proper validation and transparent description. As a result, CPMs often do not reach their full potential.

**Objectives:** We aimed to develop an open online platform enabling users to create, validate, integrate, and apply CPMs in daily medical practice.

**Methods:** An innovative platform was developed to enable registered users to add CPMs and describe them with maximum transparency in concordance with published guidelines (TRIPOD statement). On the Evidencio platform, researchers and healthcare professionals can create CPMs based on pre-specified regression formulas, custom formulas, or run R code formulas. Tools were developed to facilitate validation of online CPMs (model discrimination and calibration) based on institutional patient data. Model developers maintain ownership of their data and intellectual property of created CPMs. Methods to integrate CPMs using an application programming interface (API) were explored.

**Results:** The platform is available online and currently contains over 800 CPMs covering 30 healthcare specialties. Over 350 CPMs are available as open source. A little over 230 external validations were performed so far. Models on the Evidencio platform are easily interoperable, allowing for the calculation of multiple outcomes at once (composite models) or in a sequential format (dynamic protocols). It was found feasible to integrate CPMs on Evidencio in third party applications through an API, enabling their use in digital protocols, external websites, and the electronic health records.

**Conclusion:** An online platform for CPMs was developed and is available on www.evidencio.com. Future updates should include additional features on model recalibration following validation, semi-automatic variable mapping, and development of a patient-centered graphical representation of model outcomes.

### P29 External validation of prediction models predicting the probability of lymph node involvement in prostate cancer patients

#### T. A. Hueting^1^, E. B. Cornel^2^, D. M. Somford^3^, H. Jansen^4^, J. P. A. van Basten^3^, R. G. Pleijhuis^5^, R. A. Korthorst^6^, J. A. M. van der Palen^7^, H. Koffijberg^1^

##### ^1^Faculty of behavioural, management and social sciences, department of health technology and services research, University of Twente, Enschede, The Netherlands; ^2^Department of urology, Ziekenhuisgroep Twente, Hengelo, The Netherlands; ^3^Department of urology, Canisius Wilhelmina Ziekenhuis, Nijmegen, The Netherlands; ^4^Netherlands Comprehensive Cancer Organization, Utrecht, The Netherlands; ^5^Department of internal medicine, Medisch Spectrum Twente, Enschede, The Netherlands; ^6^Department of urology, Medisch Spectrum Twente, Enschede, The Netherlands; ^7^Faculty of behavioural, management and social sciences, department of research methodology, measurement and data analysis, University of Twente, Enschede, The Netherlands. Medisch spectrum Twente, Enschede, The Netherlands

###### **Correspondence:** T. A. Hueting

**Background:** Multiple statistical models predicting lymph node involvement (LNI) in prostate cancer (PCa) patients exist to support clinical decision-making regarding pelvic lymph node dissection (PLND).

**Objectives:** We aimed to validate existing models predicting LNI in a Dutch PCa patient cohort.

**Methods:** Sixteen prediction models were validated using a Dutch patient cohort of 1,001 men who underwent extended PLND between October 2008 and May 2017. Patient characteristics included serum prostate specific antigen (PSA), clinical tumor (cT) stage, primary and secondary Gleason scores, number of biopsy cores taken, and number of positive biopsy cores. Model performance was assessed using the area under the curve (AUC) of the receiving operator characteristic (ROC) curve. Calibration plots were used to visualize over- or underestimation of the models.

**Results:** Lymph node involvement was identified in 276 (28%) patients. Patients with LNI had a higher PSA, higher primary Gleason pattern, higher Gleason score, higher number of harvested nodes, higher number of positive biopsy cores, and higher cT stage, compared to patients without LNI. Predictions generated by the 2012 Briganti nomogram (AUC = 0.76) and the MSKCC web-calculator including biopsy core information (AUC = 0.75) were found most accurate. Underestimation of LNI probability was present when looking at patients with a predicted probability below 20%.

**Conclusion:** Models predicting LNI in PCa patients were externally validated in a Dutch patient cohort. The 2012 Briganti and the MSKCC nomograms were the most accurate prediction models available.

### P30 Optimizing the risk threshold of lymph node involvement for performing pelvic lymph node dissection in prostate cancer patients: a cost-effectiveness analysis

#### T. A. Hueting^1^, E. B. Cornel^2^, R. A. Korthorst^3^, R. G. Pleijhuis^4^, D. M. Somford^5^, J. P. A. van Basten^5^, J. A. M. van der Palen^6^, H. Koffijberg^1^

##### ^1^Faculty of behavioral, management and social sciences, department of health technology and services research, University of Twente, Enschede, The Netherlands; ^2^Department of urology, Ziekenhuisgroep Twente, Hengelo; ^3^Department of urology, Medisch Spectrum Twente, Enschede; ^4^Department of internal medicine, Medisch Spectrum Twente, Enschede; ^5^Department of urology, Canisius Wilhelmina Ziekenhuis, Nijmegen; ^6^Faculty of behavioral, management and social sciences, research methodology, measurement and data analysis, University of Twente, Enschede

###### **Correspondence:** T. A. Hueting

Background: Clinical prediction models support decision making on the performance of pelvic lymph node dissection (PLND) in prostate cancer (PCa) patients. However, international guidelines recommend different risk thresholds to select patients who may benefit from PLND.

Objectives: We aimed to quantify the cost-effectiveness of using different risk thresholds for predicted lymph node involvement (LNI) in PCa patients with the Briganti nomogram (2012) to inform decision making on omitting pelvic lymph node dissection (PLND).

Methods: Four different thresholds (2%, 5%, 10% and 20%) used in practice for performing PLND were compared using a decision analytic model, using the 20% threshold as reference. Baseline characteristics for the hypothetical cohort were based on an actual Dutch patient cohort containing 925 patients who underwent extended PLND with risks of LNI predicted by the 2012 Briganti Nomogram. Compared outcomes consisted of quality adjusted life years (QALYs) and costs. The best strategy was selected based on the incremental cost effectiveness ratio (ICER) when applying a willingness to pay (WTP) threshold of €20,000 per QALY gained. Probabilistic sensitivity analysis was performed with Monte Carlo simulation to assess the robustness of the results.

Results: Costs and health outcomes were lowest (€7,207 and 6.22 QALYs) for the 20% threshold, and highest (€9,670 and 6.27 QALYs) for the 2% threshold, respectively. The ICER for the 2%, 5%, and 10% threshold compared with the first threshold above (i.e. 5%, 10%, and 20%) were €84,974/QALY, €65,306/QALY, and €28,860/QALY, respectively. Applying a WTP threshold of €20.000,- the probabilities for the 2%, 5%, 10%, and 20% strategies being cost-effective were 0%, 3%, 28%, and 69%, respectively.

Conclusion: Applying a 20% risk threshold for probable LNI to the Briganti 2012 nomogram, to inform decision making on performing PLND in PCa patients is optimal from a health economic perspective.

### P31 How often is the value proposition stated and directly addressed in NICE’s Diagnostic Guidance?

#### I. Uchegbu^1^, C. Hyde^2^

##### ^1^Imperial MIC, Imperial College, UK; ^2^Exeter Test Group, University of Exeter, UK

###### **Correspondence:** C. Hyde

**Background**: Policy making on diagnostics presents huge challenges which organisations like NICE have tried to address. High amongst these are that diagnostics have multiple ways in which they can bring benefit to patients, carers, health services and society. This makes the task of evaluating the impact and collecting evidence on those impacts complex. One suggestion how this complexity can be handled is a clear statement of value proposition, in which developers are specific not just about how and where the new test will be used, but also what they expect will be achieved. In this way attention is directed to aspects of impact which should receive priority in the evidence development process. We intend to describe the degree to which the concept of value proposition has been used in NICE’s Diagnostic Guidance. This extends previous work on the use of end-to-end studies.

**Objectives**: To explore whether value proposition has been clearly described in NICE Diagnostic Guidance and whether evidence has been found which directly demonstrates the aspects of value proposition identified.

**Methods**: We will extend the approach used in past analysis of the methodological features of NICE guidance. All NICE diagnostics guidance will be interrogated. We will abstract data on the policy question addressed and the underlying value proposition and whether evidence has been identified on these aspects of value proposition. Analysis will be qualitative.

**Results**: This work is in progress.

**Conclusion**: Value proposition is a potentially useful way for policy makers to help make sense of the many different ways in which a test might represent an effective and cost-effective addition to health care. This project will inform the degree to which the concept is already being used by one prominent policy-maker, but also offer ways in which greater use can be made of it in the future. [299]

### P32 Prediction of Postpartum Depression Using Machine Learning methods

#### S. Ioannou^1^, G. A. Holtman^2^, L. L. Peters^2,3^, H. Burger^2^, C. H. zu Eulenburg^1^

##### ^1^Department of Epidemiology, University Medical Center Groningen, University of Groningen, The Netherlands; ^2^Department of General Practice & Elderly Care Medicine, University Medical Center Groningen, University of Groningen, The Netherlands; ^3^Department of Midwifery Science AVAG, VU University Medical Center Amsterdam, Amsterdam Public Health Research Institute, The Netherlands

###### **Correspondence:** S. Ioannou

**Background**

There is limited data in the application and use of machine learning techniques to predict postpartum depression. Furthermore, there is scarce information on how machine learning techniques can be integrated in the epidemiological framework of identifying persons at risks in clinical psychology.

**Objectives**

We explore machine learning methods to develop predictive models for postpartum depression and compare them with current methods of predictive modeling.

**Methods**

Data is obtained from the Pregnancy Anxiety and Depression (PAD) prospective cohort study designed to investigate risk factors for antenatal and postnatal anxiety and depression. We use data retrieved from 6,930 participating women by questionnaires providing information on social support, anxiety and personality traits, as well as information on socio-economic status, lifestyle, and stressful life events during pregnancy. Assessments took place at baseline, 24 and 36 weeks of gestation and 6 months postnatal. We attempt to create classification and regression models using machine learning techniques such as logistic regression, decision trees, and linear discriminant analysis to predict postpartum depression as assessed by the Edinburg Postnatal Depression scale ≥ 10. We then apply cross-validation and bootstrap techniques to compare the predictive validity, assumptions and interpretability of the methods.

**Conclusions**

This exploratory study aims to investigate the potential of machine learning methods for the prediction of postpartum depression risk in comparison to established statistical techniques with respect to suitability, applicability and accuracy of the methods. As a further step we aim to compare the predictive assessment, inferential strengths and weaknesses and statistical pitfalls that may appear from the use of such methods.

### P33 Justifying inclusion of biomarkers when designing randomised controlled trials

#### D. Johnson, A. Jorgensen

##### Department of Biostatistics, University of Liverpool

###### **Correspondence:** D. Johnson

**Background**

Biomarkers are increasingly used to personalise treatment, and biomarker-guided trials are the gold standard for testing their clinical utility. A lack of trials is one of the main obstacles delaying translation of biomarker discoveries into clinic. Before a trial takes place, there must be robust evidence for the biomarker’s validity. However, the extent of evidence required, and how it should be compiled, is unclear.

**Objectives**

We have undertaken a literature review to identify biomarker-guided randomised controlled trials (RCTs) and explore what evidence has been used to justify inclusion of a biomarker, and how the evidence was compiled.

**Methods**

We conducted a systematic search of four databases. Our search yielded 11399 papers when duplicates were removed. After screening titles and abstracts, 284 papers remained for full text screening. After full-text screening, and restricting to papers published in the past five years, 119 papers were included.

**Results**

The majority of trials were in the field of oncology (55.5%) with cardiovascular disease being the second most common (17.6%).

Many trials justified use of a biomarker based on previous retrospective or pilot studies. Others were based on evidence from literature reviews, case studies, or in vitro/in vivo work. Some trials provided strong evidence for biomarker use, citing meta-analyses and previous RCTs.

**Conclusion**

To our knowledge, no prior review has systematically identified the methods used for compiling evidence for inclusion of biomarkers in previous biomarker-guided RCTs. We have identified large variations in methods, with several RCTs based on little evidence. We have also quantified how many of each biomarker-guided design have been utilised, as well as the clinical areas in which they have been used. No standard approach exists for gathering evidence to justifying inclusion of a biomarker in RCTs, and our further work will focus on optimal approaches for doing so.

### P34 Thematic analysis in R to inform care pathway analysis: exampled with a case study on *Clostridium difficile* (*C.diff*)

#### William Jones^1^, Michael Power^2^, Joy Allen^1^

##### ^1^NIHR Newcastle In Vitro Diagnostics Co-operative, Newcastle University, Newcastle upon Tyne, UK; ^2^NIHR Newcastle In Vitro Diagnostics Co-operative, Newcastle upon Tyne Hospitals Foundation Trust, Newcastle upon Tyne, UK

**Background**

The phrase “care pathway” refers to the journey a patient takes during an episode of healthcare. Mapping the care pathway for a medical condition is a vital step in the evaluation of a new diagnostic test, helping developers identify the optimal role of their test; where it leads to greatest patient and economic benefit. Care pathways can be established through interviews with relevant medical experts, which are transcribed and analyzed thematically in software packages such as NVivo or ATLAS. These packages provide a validated environment for qualitative research, but are expensive and rigid in terms of data manipulation and analysis options, thus, have limited utility.

In a recent project we utilized the R programming language and an R package called ‘RQDA ‘to thematically analyze interviews designed to elicit expert opinion on *C.diff* testing in the UK NHS. The advantage of using R is that it is free, powerful and flexible, plus there is a strong community of users continually developing and advancing packages for use in the R environment.

**Objective**

To outline a potentially novel approach to thematic analysis in R using the RQDA package. Demonstrated with interview data from an exploratory study aimed to understand the potential role of a new point of care test for C.*diff,* within the UK NHS.

**Methods**

We interviewed 10 clinicians with expertise in the diagnosis and management of *C.diff* infection in the UK NHS. These interviews were transcribed verbatim and thematically analyzed in R, using the RQDA package.

**Results & Conclusions**

This study resulted in the explication of a potentially novel approach to thematic analysis of interviews to inform care pathway analysis for new diagnostic tests. It is our view that this new approach is systematic, scientifically reproducible and widely available, thus, a useful approach to communicate to the wider diagnostic community.

### P35 Predictive performance of multinomial logistic prediction models

#### V. M. T. de Jong^1^, M. J. C. Eijkemans^1^, B. van Calster^2^, D. Timmerman^2,4^, K. G. M. Moons^1^, E. W. Steyerberg^3^, M. van Smeden^3,5^

##### ^1^Julius Center, UMC Utrecht, Utrecht University, Utrecht, the Netherlands; ^2^Department of Development and Regeneration, KU Leuven, Leuven, Belgium; ^3^Department of Biomedical Data Sciences, LUMC, Leiden, the Netherlands; ^4^Department of Obstetrics and Gynecology, University Hospitals Leuven, Leuven, Belgium; ^5^Department of Clinical Epidemiology, LUMC, Leiden, the Netherlands

###### **Correspondence:** V. M. T. de Jong

**Background**

Multinomial Logistic Regression (MLR) has been advocated for developing clinical prediction models that distinguish between three or more unordered outcomes. Which factors drive the predictive performance of MLR is still unclear.

**Objectives**

We aim to identify the key factors that influence predictive performance of MLR models. Further, we aim to give guidance on the necessary sample size for multinomial prediction model development and on the usage of penalization during model development.

**Methods**

We present a full-factorial simulation study to examine the predictive performance of MLR models in relation to the relative size of outcome categories, number of predictors and the number of events per variable. Further, we present a case study in which we illustrate the development and validation of penalized and unpenalized multinomial prediction models for predicting malignancy of ovarian cancer

**Results**

It is shown that MLR estimated by maximum likelihood yields overfitted prediction models in small to medium sized data. In most cases, the calibration and overall predictive performance of the multinomial

prediction model is improved by using penalized MLR. Events per variable, the number of predictors and the frequencies of the outcome categories affect predictive performance.

**Conclusion**

As expected, our study demonstrates the need for optimism correction of the predictive performance measures when developing the multinomial logistic prediction model. We recommend the use of penalized MLR when prediction models are developed in small data sets, or in medium sized data

sets with a small total sample size (i.e. when the sizes of the outcome categories are balanced). Our simulation study also highlights the importance of events per variable in the multinomial context as well as the total sample size.

### P36 Biomarker Guided Trial Designs (“BiGTeD”): An online tool to help develop personalised medicine

#### Andrea Jorgensen, Ruwanthi Kolamunnage-Dona, Miranta Antoniou

##### University of Liverpool, Liverpool, UK

###### **Correspondence:** Andrea Jorgensen

**Background**

Biomarker-guided treatment is a rapidly developing area of medicine. A biomarker-guided trial is the gold standard approach to testing clinical utility of such an approach, and several biomarker-guided trial designs have been proposed. Due to the complexity of some of the designs they are often difficult to understand in terms of how they should be implemented and analysed. Further, due to the large number of different designs available, it is challenging to decide which is the most appropriate in a particular situation.

**Objectives**

To develop a user-friendly online tool, informed by a comprehensive literature review, to guide and inform those embarking on biomarker-guided trials in terms of optimal choice, design, practical application and analysis.

**Methods**

We undertook a comprehensive literature review. All unique biomarker-guided trial designs were identified and their design features, analysis approach and positive and negative qualities described. Importantly, a graphical representation of each trial design was developed, standardised to allow easy comparison of features across designs. Based on our review we developed our online tool, ‘BiGTeD’ to allow easy and free access to the information gathered.

**Results**

Our literature review identified 211 papers describing biomarker-guided trials. Information gathered during the review has been incorporated into our newly developed online tool, BiGTeD, a key feature of which is a clear and interactive graphical representation of each trial design to aid interpretation and understanding.

**Conclusions**

Navigating the literature to gain understanding of which biomarker-guided trial design to choose, and the practical implications of doing so is difficult. Our online tool, BiGTeD (www.bigted.org) is aimed at improving understanding of the various biomarker-guided trial designs and provides valuable and much-needed guidance on their implementation in a user-friendly way. Knowledge on how to design, implement and analyse these trials is essential for testing the effectiveness of a biomarker-guided approach to treatment.

### P37 Evaluation of time-dependent diagnostic efficacy of a baseline marker accounting for measurement error

#### Adina Najwa Kamarudin, Ruwanthi Kolamunnage-Dona

##### Department of Biostatistics, University of Liverpool, Liverpool, UK

###### **Correspondence:** Adina Najwa Kamarudin

Background

The standard approach of ROC (receiver operating characteristic) curve analysis considers event (disease) status and marker value for an individual as fixed over time, however in practice, both the disease status and marker value can change over time. Individuals who are disease-free earlier may develop the disease later due to longer study follow-up. Our comprehensive review on time-dependent ROC curve analysis (Kamarudin et al., 2017) found that all current estimation methods directly use the observed marker measurements by ignoring the presence of possible measurement error.

Objectives

We are proposing a novel method for evaluating the diagnostic efficacy of a marker at the baseline level accounting for measurement error.

Methods

We propose a joint modelling approach to link the individual-level deviation of the baseline marker profile from the population mean and the risk of clinical endpoint. At any time *t*, we define cases as diseased individuals prior to *t* and controls as individuals survive beyond *t*. The estimated random effects at baseline are used to define the measurement error adjusted marker. We evaluate the proposed approach in several simulation studies by varying the variance of measurement error and the strength of association between marker and risk of disease, and illustrate in real data.

Results

The proposed measurement error adjusted maker performs better over the observed marker as compared to the true area under the ROC curve (AUC) with low biases and high coverage percentages.

Conclusion

An observed marker could underestimate the true diagnostic effectiveness due to measuerement error and hence useful markers might be overlooked. The proposed methodology effectively adjust for measurement error when evaluating the diagnostic effectiveness of a marker.

Reference

Kamarudin, AN et al. (2017). “Time-dependent ROC curve analysis in medical research: current methods and applications”. BMC Med Res Methodol **17**(1): 53.

### P38 Towards AliGnment in the Reporting of Economic Evaluations of Diagnostic Tests and biomarkers: the AGREEDT checklist

#### M. M. A. Kip^1^, M. J. I. Jzerman^1^, M. Henriksson^2^, T. Merlin^3^, M. C. Weinstein^4^, C. E. Phelps^5^, R. Kusters^1,6^, H. Koffijberg^1^

##### ^1^Department of Health Technology and Services Research, MIRA institute for Biomedical Technology and Technical Medicine, University of Twente, Enschede, the Netherlands; ^2^Department of Medical and Health Sciences, Linköping University, Linköping, Sweden; ^3^Adelaide Health Technology Assessment (AHTA), School of Population Health, University of Adelaide, Adelaide, South Australia, Australia; ^4^Department of Health Policy and Management Harvard T.H. Chan School of Public Health, Boston, Massachusetts, USA; ^5^Departments of Economics and Political Science, University of Rochester, Rochester, NY, USA; ^6^Laboratory for Clinical Chemistry and Haematology, Jeroen Bosch Ziekenhuis, Den Bosch, the Netherlands

###### **Correspondence:** M. M. A. Kip

**Background**

The increasing availability of diagnostic tests and biomarkers is accompanied by an increase in health economic evaluations of these tests. However, such evaluations are typically complex and model-based because tests primarily affect health outcomes indirectly and real-world data on health outcomes are often lacking. General frameworks for conducting and reporting health economic evaluations are available but not specific enough to cover the intricacies of diagnostic test evaluation. In addition, certain aspects relevant to the evaluation may be unknown, and therefore unintentionally omitted from the evaluation. This leads to a loss of transparency, replicability, and (consequently) a loss of quality of such evaluations.

**Objectives**

To address the abovementioned challenges, this study aims to develop a comprehensive reporting checklist.

**Methods**

This study consisted of three main steps: 1) the development of an initial checklist based on a scoping review; 2) review and critical appraisal of the initial checklist by four independent experts; 3) development of a final checklist. Each item from the checklist is illustrated using an example from previous research.

**Results**

The scoping review followed by critical review by the four experts resulted in a checklist containing 43 items which ideally should be considered for inclusion in a model-based health economic evaluation. The extent to which these items were included, or discussed, in the studies identified in the scoping review varied substantially, with 13 items not being mentioned in ≥47 (75%) of the included studies.

**Conclusion**

As the importance of health economic evaluations of diagnostic tests and biomarkers is increasingly recognized, methods to increase their quality are necessary. The checklist developed in this study may contribute to improved transparency and completeness of such model-based health economic evaluations. Use of this checklist is encouraged to enhance their interpretation, comparability, and – indirectly – the validity of the results.

### P39 Impact assessment of implementing the DiagnOSAS screening tool to predict the risk of obstructive sleep apnea syndrome in primary Care

#### F. A. J. Geessinck^1^, R. G. Pleijhuis^2^, R. J. Mentink^3,^ J. van der Palen^4^, H. Koffijberg^5^

##### ^1^Master program Health Sciences, University of Twente, Enschede, The Netherlands; ^2^Department of internal medicine, Medisch Spectrum Twente, Enschede, The Netherlands; ^3^Evidencio, Haaksbergen, The Netherlands; ^4^Faculty of behavioural, management and social sciences, department of research methodology, measurement and data analysis, University of Twente, Enschede. Medical School Twente, Medisch Spectrum Twente, Enschede, The Netherlands; ^5^Faculty of behavioural, management and social sciences, dept of Health Technology & Services Research, University of Twente, Enschede, The Netherlands

###### **Correspondence:** H. Koffijberg

**Background**

Obstructive sleep apnea syndrome (OSAS) is increasingly recognized as a serious health condition. Currently, OSAS is diagnosed with polysomnography (PSG) in sleep clinics or hospitals. However, increasing waiting lists for sleep tests, and many unnecessary referrals from general practice for PSG highlight the need for alternative diagnostic strategies for OSAS.

**Objectives**

To investigate the impact of using DiagnOSAS, a screening tool to predict the risk of OSAS in individuals suspected of this condition to guide PSG referral decisions. on health outcomes and costs, and to assess its cost-effectiveness in the Netherlands compared to usual care (no screening tool).

**Methods**

A Markov cohort model was constructed to assess cost-effectiveness of the prediction tool in men aged 50 years. The diagnostic process of OSAS was simulated with and without the use of the DiagnOSAS tool, taking into account the risks and consequences of the most severe OSAS effects: car accidents, myocardial infarction and stroke. Base case cost-effectiveness was based on equal time to OSAS diagnosis with and without the use of the prediction tool. In a scenario analysis cost-effectiveness was assessed assuming that the prediction tool would halve this time to diagnosis.

**Results**

Base case results show that, within a 10 year time period, DiagnOSAS saves €226/patient at a negligible decrease in health outcomes (<0.01 quality-adjusted life years; (QALYs)), resulting in an incremental cost-effectiveness ratio of €56,997/QALY. In the scenario with time-to-diagnosis halved, DiagnOSAS dominates usual care (i.e. is both cheaper and more effective). For a willingness-to-pay threshold of €20,000/QALY the probability that using DiagnOSAS is cost-effective equals 91.7% (base case) and 99.3% (time-to-diagnosis halved), respectively.

**Conclusion**

DiagnOSAS appears to be a cost saving alternative for the usual OSAS diagnostic strategy in the Netherlands. When this prediction tool succeeds in decreasing time-to-diagnosis, it could substantially improve health outcomes as well.

### P40 Diagnostic accuracy of the PHQ-9 depression screening tool compared to semi-structured versus fully structured diagnostic interviews: an individual participant data meta-analysis comparing reference standards

#### B. Levis^1,2^, A. Benedetti^2^; K. E. Riehm^1^, N. Saadat^2^, A. Levis^1^, M. Azar^1,2^, D. B. Rice^1,2^, M. J. Chiovitti^1^, T. A. Sanchez^1^, B. D. Thombs^1,2^, and the DEPRESSD Research Group

##### ^1^Lady Davis Institute for Medical Research, Jewish General Hospital, Montréal, Québec, Canada; ^2^McGill University, Montréal, Québec, Canada

###### **Correspondence:** B. Levis

Background: Existing meta-analyses of depression screening tool accuracy have treated clinician-administered semi-structured diagnostic interviews and lay-administered fully structured diagnostic interviews as equivalent reference standards for assessing major depressive disorder (MDD). Semi-structured interviews are akin to a guided diagnostic conversation. Standardized questions are asked, but interviewers may insert additional queries and use clinical judgment to decide whether symptoms are present. In contrast, fully structured interviews are fully scripted. Standardized questions are read verbatim, without additional probes. Fully structured interviews are considered potentially more reliable but possibly less valid for MDD classification.

Objectives: To compare estimates of diagnostic test accuracy of the Patient Health Questionnaire-9 (PHQ-9) depression screening tool when semi- versus fully structured interviews are used as the reference standard.

Methods: Due to selective cutoff reporting within primary studies (Levis et al, AJE, 2017), we used an individual participant data meta-analysis approach to compare accuracy of the PHQ-9 across reference standards, using accuracy results for all cutoffs for all studies rather than only published results. Electronic databases were searched for datasets that compared PHQ-9 scores to MDD diagnosis based on validated interviews. For PHQ-9 cutoffs 5-15, we estimated pooled sensitivity and specificity among studies using semi- and fully structured interviews as the reference standard separately.

Results: Data were obtained from 43 of 53 eligible studies, for a total of 14,405 participants (1,763 MDD cases). Specificity estimates were similar across reference standards (within 2%); however, sensitivity estimates were 5-22% higher (median=18%, at standard cutoff of 10) when semi-structured interviews were used as the reference standard compared to fully structured interviews (Table 1).

Conclusion: The PHQ-9 more accurately classifies patients when compared to semi- versus fully structured interviews as the reference standard. Meta-analyses of depression screening tool accuracy should take into consideration potential differences in reference standards.

**Table 1 (abstract P40).** Diagnostic accuracy of the PHQ-9 by reference standard

	Semi-structured Reference Standard N studies = 29 N participants = 6,725 N major depression = 924		Fully structured Reference Standard N studies = 14 N participants = 7,680 N major depression = 839		Difference across reference standards (Semi- Fully)	
Cutoff	Sensitivity	Specificity	Sensitivity	Specificity	Sensitivity	Specificity
5	0.98	0.55	0.93	0.54	0.05	0.01
6	0.98	0.63	0.91	0.61	0.07	0.02
7	0.98	0.69	0.86	0.69	0.12	0.00
8	0.95	0.75	0.82	0.75	0.13	0.00
9	0.91	0.80	0.74	0.79	0.17	0.01
10	0.88	0.85	0.70	0.84	0.18	0.01
11	0.84	0.89	0.62	0.87	0.22	0.02
12	0.79	0.91	0.57	0.89	0.22	0.02
13	0.70	0.93	0.49	0.92	0.21	0.01
14	0.64	0.95	0.44	0.94	0.20	0.01
15	0.56	0.96	0.35	0.96	0.21	0.00

### P41 Are semi-structured and fully structured diagnostic interviews equivalent reference standards for major depression? An individual participant data meta-analysis comparing diagnoses across diagnostic interviews

#### B. Levis^1,2^, A. Benedetti^2^, K. E. Riehm^1^, N. Saadat^2^, A. Levis^1^, M. Azar^1,2^, D. B. Rice^1,2^, M. J. Chiovitti^1^, T. A. Sanchez^1^, B. D. Thombs^1,2^, and the DEPRESSD Research Group

##### ^1^Lady Davis Institute for Medical Research, Jewish General Hospital, Montréal, Québec, Canada; ^2^McGill University, Montréal, Québec, Canada

###### **Correspondence:** B. Levis

Background: Existing meta-analyses of depression screening tool accuracy have treated clinician-administered semi-structured diagnostic interviews and lay-administered fully structured diagnostic interviews as equivalent reference standards for assessing major depressive disorder (MDD). Semi-structured interviews are akin to a guided diagnostic conversation. Standardized questions are asked, but interviewers may insert additional queries and use clinical judgment to decide whether symptoms are present. In contrast, fully structured interviews are fully scripted. Standardized questions are read verbatim, without additional probes. Fully structured interviews are considered potentially more reliable but possibly less valid for MDD classification. No studies have assessed whether semi- and fully structured interviews differ in the likelihood that MDD will be diagnosed.

Objectives: To evaluate the association between interview method and odds of MDD diagnosis, controlling for depressive symptom scores and participant characteristics.

Methods: We analysed data collected for an individual participant data meta-analysis of Patient Health Questionnaire-9 (PHQ-9) diagnostic accuracy. Binomial Generalized Linear Mixed Models with a logit link were fit. An interaction between interview method and PHQ-9 scores was assessed.

Results: 17,158 participants (2,287 MDD cases) from 57 studies were analyzed. Among fully structured interviews, the odds of MDD diagnosis were significantly higher for the Mini International Neuropsychiatric Interview (MINI) compared to the Composite International Diagnostic Interview [OR (95% CI) = 2.10 (1.15-3.87)]. Compared to semi-structured interviews, fully structured interviews (MINI excluded) were more likely to diagnose MDD among participants with low-level depressive symptoms (PHQ-9≤6) [OR (95% CI) = 3.13 (0.98-10.00)], similarly likely among those with moderate-level symptoms (PHQ-9 7-15) [OR (95% CI) = 0.96 (0.56-1.66)], and less likely among those with high-level symptoms (PHQ-9≥16) [OR (95% CI) = 0.50 (0.26-0.97)].

Conclusion: The likelihood of MDD diagnosis appears to depend on the diagnostic interview used. Meta-analyses on depression screening tool accuracy should consider methods to account for possible differential verification bias.

**Fig. 1 (abstract P41).**
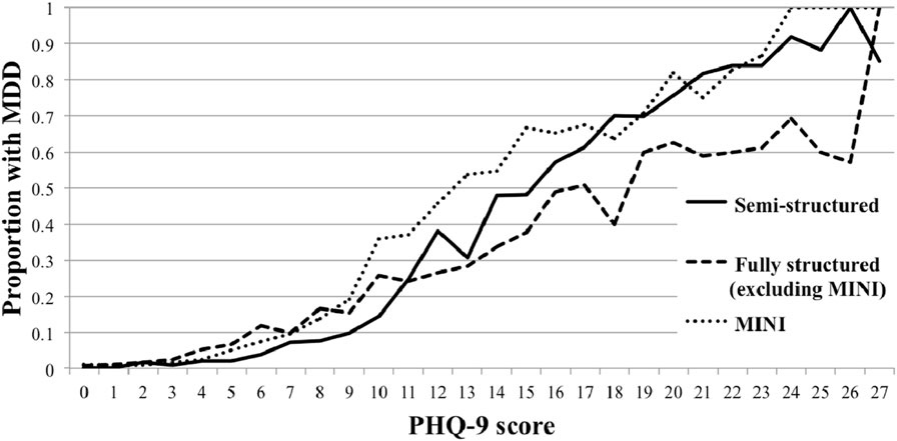
Probability of MDD diagnosis across depressive symptom levels for different diagnostic interview methods

### P42 Assessing impact of a 4th generation point of care p24 antigen HIV test

#### M. Ni^1^, S. Borsci^1^, S. Walne^1^, G. Hanna^1^

##### ^1^NIHR London IVD, Imperial College London, UK; ^2^AMC, UVA, Clinical Epidemiology, Amsterdam, the Netherlands

###### **Correspondence:** M. Ni

**Background**

HIV remains a global epidemic especially in low-to-middle income countries. The acute phase of infection is highly infectious when a patient is newly infected but the body is yet to generate sufficient amount of antibodies. Current 3^rd^ generation tests for HIV screening however miss over half of acute infections.

**Objectives**

We used stakeholder analysis to explore user scenario and carried out a cost-effectiveness analysis to explore impact of a new 4^th^ generation point of care HIV test based on detection of p24 antigen. The test is likely to be highly sensitive, and offer better linkage to care and fast time to results (under 15 min).

**Methods**

We interviewed 5 experts including 4 technology specialists and one clinician. We reviewed literature to extract evidence on early detection and treatment, quality of life and costs. We built a test-to-treatment pathway model to capture key advantages of the new test. This pathway model is then superimposed on a markov model for disease progression. We simulated a screening population of 100,000 patients over 30 years on a monthly cycle.

**Results**

Assuming a clinician-led user model in high prevalence countries, comparing to the 3^rd^-gen test, the new test averted 15 transmissions per 100,000 screening population. Its cost-effectiveness, as measured by the incremental cost-effectiveness ratio (ICER), is US$39,785 per QALY, or US$35,684 per QALY, when transmission was taken into account. The main driver of the cost-effectiveness lies with the characteristics of the screening populations. ICER reached $100,534 per QALY gained when the test population has an HIV/AIDS prevalence at 1%.

**Conclusion**

By decomposing test-to-treatment pathway, we are able to quantify the impact of test characteristics, such as better linkage to care. This new, more sensitive screening test, has the potential to achieve cost-effectiveness through target screening in a high prevalence setting.

### P43 Evaluating the impact of malaria rapid diagnostic tests on patient-important outcomes: Study design and fidelity considerations

#### E. A. Ochodo^1^, S. Naidoo^1^, S. G. Schumacher^2^, C. Nsanzabana^2^, T. Young^1,3^, S. Mallett^4^, F. C. Cobelens^5^, P. M. Bossuyt^6^, J. Deeks^4^, M. P. Nicol^7,8^

##### ^1^Centre for Evidence-based Health Care, Division of Epidemiology and Biostatistics, Faculty of Medicine and Health Sciences, Stellenbosch University, Cape Town, South Africa; ^2^Foundation for Innovative New Diagnostics, Campus Biotech, Geneva, Switzerland; ^3^Cochrane South Africa, South African Medical Research Council; ^4^Institute of Applied Health Research, Public Health Building, University of Birmingham, Edgbaston, Birmingham, UK; ^5^Amsterdam Institute for Global Health and Development, Academic Medical Centre, Amsterdam, The Netherlands; ^6^Department of Clinical Epidemiology, Biostatistics and Bioinformatics, Academic Medical Centre, Amsterdam, The Netherlands; ^7^Division of Medical Microbiology, University of Cape Town, South Africa; ^8^National Health Laboratory Service, Johannesburg, South Africa

###### **Correspondence:** E. A. Ochodo

**Introduction:** Evaluations of the impact of malaria rapid diagnostic tests (RDTs) have shown beneficial effects of RDTs on intermediate process outcomes, such as reduced time to diagnosis and treatment, but limited impact on later stage patient outcomes, such as morbidity and mortality. These unclear benefits could be partly due to shortcomings in study design and factors influencing intervention fidelity (extent to which the test-treatment intervention is delivered as designed). We aim to critically review the designs, outcome measures and intervention fidelity of studies evaluating the impact of malaria RDTs on patient-important outcomes and explore factors that may influence intervention fidelity.

**Methods:** We are conducting a systematic review of quantitative and qualitative studies. We have searched relevant electronic databases and grey literature and included studies based on predefined inclusion criteria. To evaluate the methodological quality of included studies, we are using the revised Cochrane risk of bias tool for randomized studies (ROB 2.0), the revised Cochrane risk of bias tool for non-randomised studies of interventions (ROBINS-I), the checklist to assess implementation (Ch-IMP) for assessing the quality of intervention delivery and an adaptation of the Critical Appraisal Skills Programme (CASP) tool for qualitative studies. Two authors have reviewed the search output and are currently extracting data and assessing methodological quality independently, resolving any disagreements by consensus. We will synthesize information from quantitative studies narratively and through descriptive statistics and use a thematic framework analysis approach for qualitative studies.

**Results and Discussion:** Our electronic searches yielded 2731 hits of which 123 studies (quantitative (n=72) and qualitative (n=51)) have been included for data extraction. We will present the review results, including a graphical classification of key methodological issues affecting malaria RDT impact studies (see logic framework in Fig. 1) with discussion of considerations for selecting and interpreting a particular study design.

**Fig. 1 (abstract P43).**
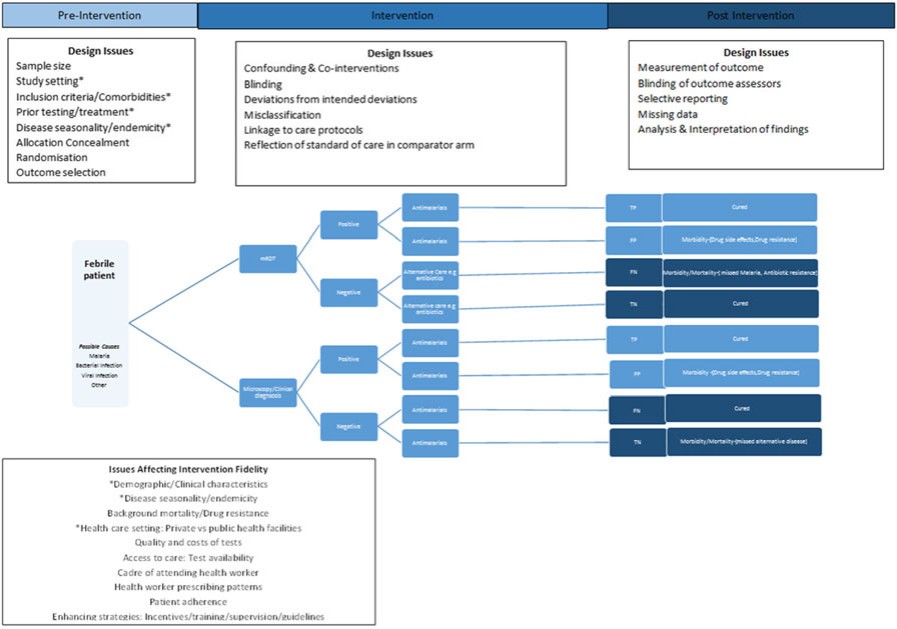
Design & Fidelity Considerations when measuring impact of malaria RDTs on Morbidity/Mortality compared to clinical or microscopy diagnosis

### P44 Investigating risk of bias: Special considerations for test-treatment interventions

#### E. A. Ochodo^1^, S. Naidoo^1^, S. G. Schumacher^2^, C. Nsanzabana^2^, T. Young^1,3^, S. Mallett^4^, F. C. Cobelens^5^, P. M. Bossuyt^6^, M. P. Nicol^7,8^, J. Deeks^4^

##### ^1^Centre for Evidence-based Health Care, Division of Epidemiology and Biostatistics, Faculty of Medicine and Health Sciences, Stellenbosch University, Cape Town, South Africa; ^2^FIND, Geneva, Switzerland; ^3^Cochrane South Africa, South African Medical Research Council; ^4^Institute of Applied Health Research, Public Health Building, University of Birmingham, Edgbaston, Birmingham, UK; ^5^Amsterdam Institute for Global Health and Development, Academic Medical Centre, Amsterdam, The Netherlands; ^6^Department of Clinical Epidemiology, Biostatistics and Bioinformatics, Academic Medical Centre, Amsterdam, The Netherlands; ^7^Division of Medical Microbiology, University of Cape Town, South Africa; ^8^National Health Laboratory Service, Johannesburg, South Africa

###### **Correspondence:** E. A. Ochodo

**Introduction:** Evaluating the impact of diagnostic tests on patients’ health is complex. Due to the multiple steps involved between the decision to administer a test and effect on patient’s health, a broad range of outcomes can be measured in studies that evaluate the impact of tests on a patient’s health, and various forms of bias can be introduced along this pathway. The revised Cochrane risk of bias tool for randomized studies (RoB 2.0), and that for non-randomised studies of interventions (ROBINS-I), focus on risk of bias (RoB) assessment in general but do not point out issues specific to test-treatment interventions which are a distinct type of complex intervention. We describe our experience in using the Cochrane RoB tools to investigate bias in primary studies evaluating the impact of malaria rapid diagnostic tests (RDTs) on patient-important outcomes.

**Methods:** We searched relevant electronic databases and grey literature and included studies based on predefined inclusion criteria. We included any primary randomized or non-randomized study that compared a malaria RDT with one or more other diagnostic tests for malaria, with an aim of measuring the impact of these tests or strategies on patient-important outcomes. We are currently extracting data and using the ROB 2.0 tool for randomized studies and the ROBINS-I for non-randomised studies of interventions to assess RoB of included test treatment studies. Two authors have reviewed the search output and are currently extracting data and assessing RoB independently, resolving any disagreements by consensus. We will present our assessment of RoB across each domain and overall RoB results for included studies narratively, graphically and by descriptive statistics.

**Results and Discussion:** Our data set contains 27 randomised studies and 22 non-randomised studies. During the conference we will present our RoB results as well as discuss special considerations for investigating the RoB in test-treatment studies.

### P45 Methods to facilitate the interpretation of pooled diagnostic test accuracy estimates by means of selecting a representative pre-test probability

#### M. S. Oerbekke^1,2^, A. van Enst^1^, K. Jenniskens^3^, R. J. P. M. Scholten^2^, L. Hooft^2^

##### ^1^Knowlegde Institute for Medical Specialists, Utrecht, The Netherlands; ^2^Cochrane Netherlands, Julius Center for Health Sciences and Primary Care, University Medical Center Utrecht, Utrecht, The Netherlands; ^3^Julius Center for Health Sciences and Primary Care, University Medical Center Utrecht, Utrecht, The Netherlands

**Background:** For a clinician, diagnostic test results alone are not informative unless they are able to estimate the prevalence in their setting. Diagnostic Test Accuracy (DTA) reviews are facilitating the interpretation of the pooled test performance using a pretest-probability in a hypothetical cohort. However, it is unknown what methods are used in DTA reviews to select the target condition’s pre-test probability.

**Objectives:** To assess what methods in Cochrane DTA reviews are used for selecting a pre-test probability to demonstrate a test’s performance using summary sensitivity and/or specificity.

**Methods:** DTA reviews were selected from the Cochrane Library on the 2^nd^ of February, 2018. Reviews were eligible when a pooled or summarized accuracy measure was provided. Data were extracted by one author and checked by a second author.

**Preliminary results:** From 81 DTA reviews 59 reviews were eligible comprising 307 meta-analyses. The following methods for selecting a pre-test probability were observed: using one point estimate from included studies (median [62 analyses], mean [11 analyses]), using a point estimate and a measure of dispersion (median and range [1 analysis], mean and range [1 analysis], median and lower/upper quartile [3 analyses], median and lower/upper quartile and range [5 analyses]), using literature (studies reporting prevalence [15 analyses], WHO suggestion [10 analyses], guideline [4 analyses]), using an assumption (27 analyses), or using an unclear or partially unclear method (12 analyses).

**Preliminary conclusions:** This is an ongoing study and updated results and conclusions will be presented during the conference. No consensus currently exists on what method should be used to select a representative pre-test probability. However, it is probably more informative to use multiple pre-test probabilities from data included for analyses (e.g. a point estimate and a measure of dispersion). Multiple pre-test probabilities could facilitate the test’s performance interpretation for clinicians in their own practice.

### P46 Bias in excess-incidence methods for estimating over- diagnosis: a framework for evaluating screening trials

#### Jason Oke, Brian D. Nicholson and Richard Stevens

##### Nuffield Department of Primary Care Health Sciences, University of Oxford, UK

**Background**

Overdiagnosis can be defined as a screen-detected cancer that would have not been detected in the absence of screening. It can be estimated by the “excess-incidence” in the screened arm of a control trial but no consensus exists on how exactly this should be done.

**Objectives**

To determine the potential biases associated with excess-incidence estimates of overdiagnosis under different scenarios in the screening and post-screening period.

**Methods**

Cancer was assumed to progress from an undetectable state, to a pre-clinical state and finally to a clini- cal state as first described by Zelen and Feinleib (1969). Screening participants were categorised on the basis on their state before screening started, whether the disease progressed to the clinical state during the study, in the period following screening (relevant cases) or not at all (overdiagnosed). Standard math- ematical manipulations were used to assess the sources bias under four different scenarios 1) excess in screening arm immediately following the end of screening 2) removing the prevalence round cases

3) Complete follow-up 4) complete follow-up but where trial participants access screening after the trial finishes.

**Results**

Excess incidence in the screening at the end of screening (scenario 1) is biased upwards for overdiag- nosis as it includes prevalent and incident round cases with clinical disease that would have arisen after screening. Scenario 2 is biased as it fails to include all relevant and overdiagnosed cancers. Scenario 3 is unbiased but only in the absence of screening in the period after the end of the trial as per scenario 4.

**Conclusion**

Estimates of overdiagnosis using the excess-incidence approach are subject to bias. Studies that follow- up both cohorts provide unbiased estimates but only if screening is not accessed in the post-screening period.

### P47 Biomarkers for monitoring testicular cancer recurrence

#### José M. Ordóñez-Mena, Thomas Fanshawe, Brian Nicholson

##### Nuffield Department of Primary Care Health Sciences, University of Oxford, UK

###### **Correspondence:** José M. Ordóñez-Mena

**Background** Human chorionic gonadotropin (HCG), α-fetoprotein (AFP) and lactate dehydrogenase (LDH) are established biomarkers for testicular cancer prognosis and monitoring. However, there is a lack of high-quality datasets to provide evidence on their optimal use in monitoring for recurrent disease.

**Methods** Routinely collected cohort data from the Oxford University Hospitals were available for 508 stage I to IV testicular cancer patients aged 16 to 83 years in whom information from 1,642 imaging tests and 2,204 tumour marker tests were available. We sought to investigate the relationship between HCG, AFP and LDH biomarkers measured during monitoring following curative surgery and testicular cancer recurrence.

**Preliminary Results** Between 2004 and 2016, 58 patients (11.4%) experienced a cancer recurrence. Tumour biomarkers were assessed mainly at post-chemotherapy (n=376) and during surveillance (n=917). Median and IQR number of tests during the study period was 4 (2-6) tests.

**Interpretation** This large dataset consisting of comprehensive longitudinal follow-up following initial treatment provides an opportunity to reassess the use of biomarkers in monitoring schemes for testicular cancer recurrence. Testicular cancers recurred in a small proportion of these patients which suggests many may be undergoing unnecessary testing.

### P48 Shortcomings in the Evaluation of Biomarkers in Ovarian Cancer: A Systematic Review

#### Maria Olsen^1^, Mona Ghannad^1,2^, Christianne Lok^3^ and Patrick M. Bossuyt^1^

##### ^1^Academic Medical Center Amsterdam, Dept. of Clinical Epidemiology, Biostastistics and Bioinformatics, Amsterdam Public Health Research Institute, Meibergdreef 9, 1105 AZ Amsterdam, The Netherlands; ^2^Centre de Recherche Épidémiologie et Statistique Sorbonne Paris Cité, Université Paris Descartes, Centre d'épidémiologie Clinique, Hôpital Hôtel-Dieu, Paris, France; ^3^Center Gynaecologic Oncology Amsterdam, Location Antoni van Leeuwenhoek/Netherlands Cancer Institute, Dept. of Gynaecologic Oncology, Plesmanlaan 121, 106CX, Amsterdam, The Netherlands

###### **Correspondence:** Maria Olsen (m.olsen@amc.nl)

**Background:** Shortcomings in study design have been hinted at as one of the possible causes of failures in translation of discovered biomarkers into clinical use, but systematic assessments of biomarker studies are scarce.

**Objective:** We wanted to document study design features of recently reported evaluations of biomarkers in ovarian cancer.

**Methods:** We performed a systematic search in PubMed (MEDLINE) for recent reports of studies evaluating the clinical performance of putative biomarkers in ovarian cancer. We extracted data on design features and study characteristics.

**Results:** Our search resulted in 1,026 studies; 329 (32%) were found eligible after screening, of which we evaluated the first 200. Of these, 93 (47%) were single center studies. The median sample size was of 156 (minimum 13 to maximum 50,078). Few studies reported eligibility criteria (17%), sampling methods (10%) or a sample size justification power calculation (3%). Studies often used disjoint groups of patients, sometimes with extreme phenotypic contrasts; 46 studies included healthy controls (23%), but only 5 (3%) had exclusively included advanced stage cases.

**Conclusions:** Our findings confirm the presence of suboptimal features in recent evaluations of the clinical performance of ovarian cancer biomarkers, and the need for a greater awareness of these issues. Accordingly, this may lead to premature claims about the clinical value of these markers or the risk of discarding other potential biomarkers that are urgently needed.

### P49 Risk prediction models to aid cancer diagnosis in primary care are available for use, but to what extent have they been evaluated?

#### B. Grigore^1^, R. Lewis^2^, S. Robinson^3^, J. Lowe^3^, J. L. Peters^1^, A. Spencer^4^, W. Hamilton^5^, S. Price^5^, A. Medina-Lara^4^, C. Hyde^1^

##### ^1^Exeter Test Group, University of Exeter, UK; ^2^North Wales Centre for Primary Care Research, Bangor University, Wrexham, UK; ^3^PenTAG, University of Exeter, UK; ^4^Health Economics Group, University of Exeter, UK; ^5^DISCO (Diagnosis of Symptomatic Cancer Optimally), University of Exeter, UK

###### **Correspondence:** C. Hyde

**Background**: Prediction models for cancer have been developed into tools to aid GP decision-making on referral of symptomatic patients in primary care. This includes mouse-mats, flip-charts, an electronic system for the Risk Assessment Tool (RAT) and an electronic system for Qcancer. Although these tools are available to GPs in the UK, an exploration of their effectiveness, and any validation of the underlying prediction models is lacking.

**Objectives**: To discuss the impact of available evidence on informing decisions on when prediction models are ready for use in practice, using examples from our recent systematic review of the clinical effectives of cancer risk prediction tools to aid decision making in primary care.

**Methods**: We conducted two systematic reviews to assess: 1) the effectiveness of tools, 2) the validation of the prediction models. The systematic reviews identified evidence on any tool/prediction model that met our inclusion criteria, but here we focus on just the two models (and associated tools) already available to GPs: Qcancer and RATs. Electronic databases were searched, hits double-screened, data extracted and risk of bias of included studies was assessed.

**Results**: 2 studies investigated the effectiveness of the RATs tool, one suggesting an increase in rapid referrals and investigations with the tool, the other suggesting no impact on time to diagnosis compared to no use of the tool. We found no studies investigating the impact of the Qcancer tool.

The majority of Qcancer prediction models had been validated externally, by researchers not involved in the development of the models, and showed good performance. We did not find any external validation of the RATs models.

**Conclusion**: Prediction models for cancer diagnosis in primary care are available for GPs to use, but neither has been fully evaluated or validated. We will highlight these gaps and discuss implications for further work and policy-making.

### P50 Graphical representation of interobserver variability study results

#### L. Quinn^1^, S. Mallett^1^, S. A. Taylor^2^, G. Bhatnagar^2^

##### ^1^Institute of Applied Health Sciences, University of Birmingham, Edgbaston, Birmingham B15 2TT, United Kingdom; ^2^Centre for Medical Imaging, UCL, 3rd Floor East, 250 Euston Road, London, NW1 2PG, United Kingdom

**Background**

Interobserver variability studies estimate variation between results in which more than one observer interprets the same data. Agreement in medical imaging interpretation is very important, particularly whether radiologists agree on the presence or absence of disease in an imaging dataset. Generally, interobserver variability study results are shown in a table, using statistical measures such as kappa and percentage agreement. A table format is however very limiting when presenting data from multiple observations made in the same patient especially where data includes disease location.

**Objectives**

We propose two graphical representations to better encapsulate the results of complex interobserver variability studies, and improve data accessibility

**Method**

We performed a preliminary analysis of data from an interobserver variably study of small bowel ultrasound in diagnosing and staging Crohn’s disease, performed as part of a larger diagnostic accuracy study (the METRIC trial). A subset of recruited patients underwent two ultrasound examinations performed and interpreted by two different radiologists. Radiologists documented the presence or absence of disease in 10 pre-defined bowel segments. For the reference standard, an expert consensus panel decided the patient disease status based on all clinical data collated during six months patient follow up. We developed novel graphical methods to present the interobserver data.

**Results**

We will present two different graphical presentations from the analysis we have completed. One shows where observers agreed and disagreed on disease location with the consensus panel results. The other shows agreement and disagreement by disease location separately for disease positive and disease negative locations. We also give examples of how this method could be extended to other similar scenarios.

**Conclusion**

Graphical representation of interobserver variability could improve understanding of the results and may provide more informative results than current summary statistics alone.

### P51 Prediction of Delayed Cerebral Ischemia in Patients With Subarachnoid Hemorrhage using Auto-encoders and Machine Learning Algorithms

#### L. A. Ramos^1,3^, W. E. van der Steen^1,2,4,5^, R. Sales Barros^1,3^, C. B. Majoie^2^, R. van den Berg^2^, D. Verbaan^3^, W. P. Vandertop ^4^, I. J. A. Zijlstra^2^, A. H. Zwinderman^3^, G. J. Strijkers^1,2^, S. Delgado Olabarriaga^3^, H. A. Marquering^1,2^

##### ^1^Department of Biomedical Engineering & Physics, Academic Medical Center, Amsterdam, The Netherlands; ^2^Department of Radiology and Nuclear Medicine, Academic Medical Center, Amsterdam, The Netherlands; ^3^Department of Clinical Epidemiology, Biostatistics and Bioinformatics, Academic Medical Center, Amsterdam, The Netherlands; ^4^Neurosurgical Center Amsterdam, Academic Medical Center, Amsterdam, The Netherlands; ^5^Department of Neurology, Academic Medical Center, Amsterdam, The Netherlands

###### **Correspondence:** L. A. Ramos

This abstract has been previously published in European Stroke Journal, 2018; 3(1): 3-204. DOI:10.1177/2396987318770127 http://journals.sagepub.com/doi/10.1177/2396987318770127

### P52 Few promising multivariable prognostic models for recovery of people with nonspecific neck pain in musculoskeletal primary care: a systematic review

#### Roel W. Wingbermühle^1,2^, Emiel van Trijffel^1,3^, Paul M. Nelissen^1^, Bart Koes^2^, Arianne P. Verhagen^2^

##### ^1^SOMT University of Physiotherapy, The Netherlands; ^2^Erasmus University Medical Centre, The Netherlands; ^3^Vrije Universiteit Brussels, Belgium

###### **Correspondence:** Roel W. Wingbermühle

This abstract has been previously published in Journal of Physiotherapy 2018; 64:18-23. DOI: 10.1016/j.jphys.2017.11.013 http://linkinghub.elsevier.com/retrieve/pii/S1836955317301443

### P53 Use of the MODY probability calculator in a real world setting

#### B. M. Shields^1^, T. J. McDonald^2^, M. H. Shepherd^1^, M. Hudson^1^, K. Colclough^3^, S. Ellard^3^, A. T. Hattersley^1^

##### ^1^NIHR Clinical Research Facility, University of Exeter Medical School, Exeter, UK; ^2^Blood Sciences, Royal Devon and Exeter NHS Foundation Trust, Exeter, UK; ^3^Molecular Genetics Laboratory, Royal Devon and Exeter NHS Foundation Trust, Exeter, UK

###### **Correspondence:** B. M. Shields

**Background**: MODY is a rare, young-onset, genetic form of diabetes. Diagnosing MODY is important to ensure appropriate treatment, but identifying MODY patients is challenging. Diagnostic testing is expensive, prohibiting universal testing. We developed the MODY probability calculator (https://www.diabetesgenes.org/mody-probability-calculator/, >34000 visitors to date), a validated model that calculates probability of MODY based on clinical features, to help clinicians prioritise which patients to refer for diagnostic testing.

**Objectives/Methods**: To assess the use of the MODY calculator in the real world setting: 1) its performance in a population cohort of patients diagnosed <30y (n=1407), 2) its utility in clinical referrals sent to the Exeter molecular genetics diagnostic laboratory for MODY testing (n=1285) between 1/8/14 and 31/12/17.

**Results**: 1) In the population cohort, 51/1407 (3.6%) were diagnosed with MODY; 1293 (45 MODY) had sufficient data to calculate their MODY probability. The model performed well (ROC AUC=0.9) and showed good calibration (Hosmer-Lemeshow p=0.24). 39/397 (10%) individuals with probabilities >3.6% had MODY (87% sensitivity, 69% specificity for this cutoff). 14/21 (67%) individuals with >75% probability had MODY (31% sensitivity, 99% specificity).

2) In the diagnostic laboratory, 621/1285 (48%) referrals reported use of the calculator. Referrals that stated they had used the calculator had a higher pick-up rate of MODY than those that did not (33% v 25%, p=0.002). MODY probability could be calculated on 425/664 referrals that did not use the calculator. The mean probability was lower compared with referrals that had used the calculator (16.5% v 42.6%, p<0.001).

**Conclusion**: The MODY model appears to work well in a population setting, although analysis was limited by small numbers of MODY patients. The MODY model is frequently being used prior to sending referrals for MODY testing. Referrals that use the calculator appear to be more appropriate, with higher probabilities and a higher pick up rate of MODY.

### P54 Sample size guidance and justification for studies of biological variability (BV)

#### A. Sitch^1^, S. Mallett^1,2^, J. Deeks^1,2^

##### ^1^Test Evaluation Research Group, Institute of Applied Health Research, University of Birmingham, Birmingham, UK; ^2^National Institute for Health Research (NIHR) Birmingham Biomedical Research Centre

###### **Correspondence:** A. Sitch

**Background**

Biological variability (BV) studies aim to measure variability in a biomarker between and within individuals. Knowledge of BV allows the potential for a biomarker to diagnose and monitor disease to be assessed. Sample sizes for BV studies involve stating numbers of participants (*n*_1_), observations per participant (*n*_2_) and repeat assessments of each observation (*n*_3_). Little guidance exists to compute these values.

**Objectives**

To assess the precision of estimates at different sample sizes to provide guidance when planning studies.

**Methods**

Data were simulated following the model *y*_*ijk*_ = *μ* + *α*_*i*_ + *β*_*ij*_ + *ε*_*ijk*_, with mean *μ*, ${\alpha}_i\sim N\left(0,{\sigma}_G^2\right)$, ${\beta}_{ij}\sim N\left(0,{\sigma}_I^2\right)$, ${\varepsilon}_{ijk}\sim N\left(0,{\sigma}_A^2\right)$ and *i* = 1, . . , *n*_1_, *j* = 1, . . , *n*_2_ and *k* = 1, . . , *n*_3_.

Data were simulated varying sample sizes, and random effects models estimated between-individual (*σ*_*G*_), within-individual (*σ*_*I*_) and analytical variation (*σ*_*A*_), along with coefficients of variation (CVs), index of individuality (II) and reference change values (RCV). After 1,000 simulations results were analysed to assess the variation and accuracy of the estimates.

**Results**

Increasing participants decreases the range of estimates for *σ*_*A*_, *σ*_*I*_ and *σ*_*G*_; increasing observations decreases the range of *σ*_*A*_ and *σ*_*I*_, however the range of estimates of *σ*_*G*_ appeared constant. Increasing assessments decreases the range of *σ*_*A*_ with the range of *σ*_*I*_ and *σ*_*G*_ unchanged.

Increasing participants and observations decreases the range of estimates of II and RCV. II was overestimated with few participants. Increases in assessments made little change in the range of estimates of II and RCV.

We have produced a shiny app which allows precision of estimates to be estimated for given parameter values: https://alicesitch.shinyapps.io/bvs_simulation/.

**Conclusion**

Sample size decisions for BV studies can use a precision based approach. Changing numbers of participants, assessments and observations impacts on the precision of different estimates. Increasing the number of participants increases precision for all estimates. Simulation of the range of results obtained for a given sample size can guide planning of studies.

### P55 Assessing the impact of measurement uncertainty on test outcomes

#### A. F. Smith^1,2^, M. P. Messenger^1,2^, P. S. Hall^3^, B. Shinkins^1,2^, C. T. Hulme^1,2^

##### ^1^Academic Unit of Health Economics, University of Leeds, UK; ^2^NIHR Leeds In Vitro Diagnostic Co-operative, UK; ^3^Cancer Research UK Edinburgh Centre, UK

###### **Correspondence:** A. F. Smith

**Background**:

Systematic and/or random errors in test measurement (collectively ‘measurement uncertainty’) can result from various factors along the testing pathway, from the time of day a test sample is taken to the specific platform used for sample analysis. The consequence of this uncertainty is that any observed test value may differ to the underlying ‘true’ target value. Crucially, although this uncertainty can significantly affect clinical accuracy and utility, it is rarely considered in test outcome/impact studies.

**Objectives**:

To identify current methodology utilized in studies assessing the impact of measurement uncertainty on test outcomes (including clinical accuracy, clinical utility and cost-effectiveness).

**Methods**:

A literature review – using MEDLINE, Embase, Web of Science and Biosis – was used to identify relevant studies published in the last 10 years. Subsequent citation tracking was conducted to identify additional material (published any date). Ongoing data extraction is focused on identifying study aims, methods (in particular the components of measurement uncertainty addressed, data sources, input values and distributional assumptions) and the impact of measurement uncertainty on baseline results.

**Results**:

Based on interim findings, 45 studies conducted across a range of settings and indications have been identified. The majority utilize simulation techniques to explore the impact of measurement bias (systematic error) and/or imprecision (random error) on clinical accuracy or utility. Typically these draw on an ‘error model’ in which bias is assumed fixed and imprecision normally distributed, e.g.:

$$ {\mathrm{Test}}_{\mathrm{simulated}}={\mathrm{Test}}_{\mathrm{true}}+\left[{\mathrm{Test}}_{\mathrm{true}}\times \mathrm{CV}\times \mathrm{N}\left(0,1\right)\right]+\mathrm{Bias} $$

[where CV = coefficient of variation and N(0,1) = a random draw from a normal distribution (mean 0, standard deviation 1)]. Both bias and imprecision have been reported to have a significant impact on test outcomes within these studies.

**Conclusions**: Analysis of the final results will enable identification of key methodological considerations for future applications and research in this field.

### P56 Assessment of the clinical performance of adding high-sensitivity cardiac troponin to risk assessment tools for primary prevention of cardiovascular disease

#### L. P. Staub^1^, A. St John^2^, S. Lord^1^

##### ^1^NHMRC Clinical Trials Centre, University of Sydney, Sydney, Australia; ^2^ARC Consulting, Perth, Australia

###### **Correspondence:** L. P. Staub

**Background**:

Clinical guidelines recommend cardiovascular disease (CVD) risk assessment to identify patients who will benefit from lifestyle advice +/- drug therapy. As current risk tools are not perfect, high-sensitivity cardiac troponin (hs-cTn), an independent predictor of CVD risk, has been suggested to improve risk classification for individuals at average risk.

**Objectives**:

To assess whether the clinical performance of risk assessment tools including hs-cTn supports further investigation for clinical use.

**Methods**:

We searched MEDLINE to identify studies comparing the performance of validated CVD risk assessment tools in the adult general population when adding hs-cTn. We extracted data on troponin, risk tools, risk categories, risk of bias, and performance measures: discrimination, calibration and reclassification. We summarised the proportion more correctly up (TP) or downgraded (TN), falsely up (FP) or downgraded (FN) with the addition of hs-cTn. We used the treatment threshold of 10% 10-year CVD risk to dichotomise low versus high risk. We calculated the number needed to screen (NNS) to avoid one additional cardiovascular event. We defined the minimum acceptable troponin model performance to support further investigation as a higher TP rate and/or a higher TN rate. We considered the potential benefits of an additional TP higher than an additional TN. In case of a trade-off we considered < 10 FP:1 TP acceptable.

**Results**:

Two studies reported adequate data for our analysis. Both reported a net improvement in TP and TN rate and modest reduction in NNS (Table 1). Neither reported troponin model performance in an external validation population.

**Conclusion**:

Two studies provide consistent evidence that including troponin in two different CVD risk assessment tools improves discrimination of patients who will/will not develop CVD to guide management at the risk threshold of 10% 10-year risk. These findings warrant further investigation, including external validation, for assessment of clinical benefits, harm and cost-effectiveness.

**Table 1 (abstract P56).** See text for description

Study	N	Comparator tool	Index test	Additional TP FP TN FN	NNS
Zeller 2014	12,650 CVD: 853 No CVD: 11,797	ASSIGN SCORE variables	ASSIGN + hs-cTn	TP 83 FN 42 TN 564 FP 537 Increase in TP rate 41/853 = 4.8% (3.37–6.23) Increase in TN rate 27/11797 = 0.2% (0.12–0.28) TP:FP = 1:6.5 TN:FN = 13.4:1	ASSIGN 218 ASSIGN + hs-cTn 198
Blanken-berg 2016	60,444 CVD: 3178 No CVD: 57,266	ESC SCORE variables	ESC SCORE + hs-cTn	TP 64 FN 31 TN 461 FP 362 Increase in TP rate 33/3178 = 1.0% (0.65–1.35) Increase in TN rate 99/57266 = 0.2% (0.16–0.24) TP:FP = 1:5.7 TN:FN = 14.9:1	ESC SCORE 237 ESC SCORE + hs-cTn 233

### P57 Using clinical registry data to develop outcome prediction models in spine surgery

#### L. P. Staub^1^, A. F. Mannion^2^, E. Aghayev^2^, Veronika Skrivankova^3^, S. Lord^1^

##### ^1^NHMRC Clinical Trials Centre, University of Sydney, Sydney, Australia; ^2^Spine Center Division, Schulthess Klinik, Zurich, Switzerland; ^3^Institute of Social and Preventive Medicine, University of Bern, Bern, Switzerland

###### **Correspondence:** L. P. Staub

**Background**:

Spine surgeons need to be able to make evidence-based predictions on the outcome of surgery. The risks (e.g. complications) and benefits (e.g. pain alleviation) of treatment modalities have to be adequately communicated to their patients. There is a lack of validated prognostic tools to support spine surgeon and patient decisions in daily practice. Evidence on predictors for surgical outcomes is available; however, to date no studies have developed comprehensive clinical prediction models for spine surgery.

**Objectives**:

To use data from a spine unit collected within a large clinical spine registry to develop outcome prediction models for patients undergoing surgery after lumber disc herniation.

**Methods**:

We built lasso regression models to identify relevant predictors and estimate the parameters for 12-month-outcome prediction of a quality of life (QoL) score, back and leg pain scores, surgical complications, and patient satisfaction. A freely available online prognostic tool was developed to present the predicted outcomes for individual patients, based on their pre-operative characteristics.

**Results**:

Data from 1127 patients (mean age 49yrs, 42% female) was used for model development. Number of previous spine surgeries, insurance class (private vs general), body-mass index, and preoperative leg pain were the strongest outcome predictors in most models. The R^2^ of the models ranged from 0.16 to 0.21. A preliminary online tool was programmed for QoL and pain scores (Fig. 1).

**Conclusion**:

Clinical use of the tool requires further validation. Temporal validation of the models in the same spine unit is underway. Prospective collection of additional factors is planned to improve prediction precision. The main challenges include how and when to update models in ongoing data collection; generalisability of models to other clinics; and the limitation of this observational single-arm cohort study, which does not allow treatment comparisons.

**Fig. 1 (abstract P57).**
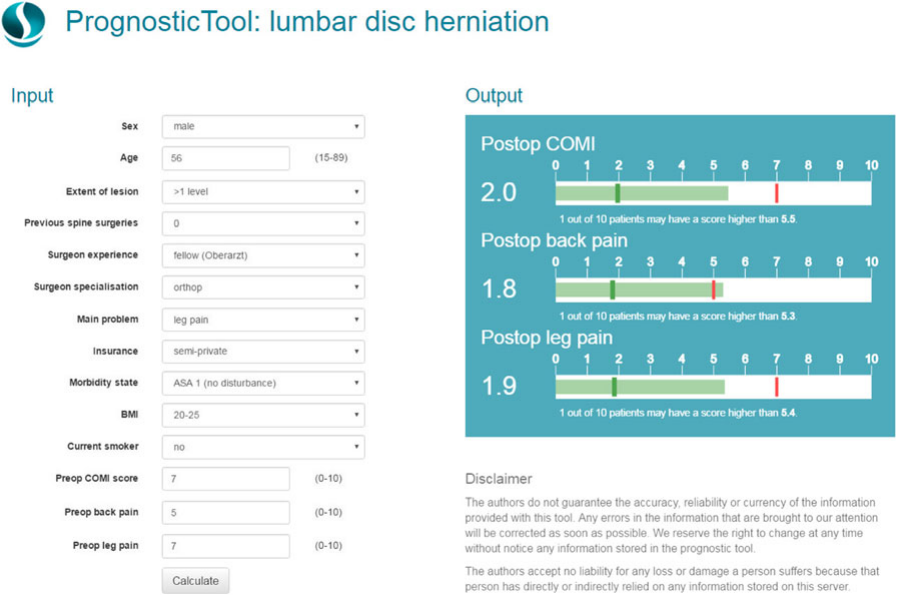
See text for description

### P58 Latent class meta-analysis in a Cochrane review for improving accuracy estimates in the absence of a perfect reference standard

#### Karen R. Steingart^1^, Mikashmi Kohli^2^, Ian Schiller^3^, Samuel G. Schumacher^4^, Nandini Dendukuri^3^

##### ^1^Cochrane Infectious Diseases Group, Liverpool School of Tropical Medicine, Liverpool, UK; ^2^Department of Epidemiology, Biostatistics and Occupational Health, McGill University, Montreal, Canada; ^3^Division of Clinical Epidemiology, McGill University Health Centre - Research Institute, Montreal, Canada; ^4^FIND, Geneva, Switzerland

###### **Correspondence:** Karen R Steingart

**Background:** Extrapulmonary tuberculosis (TB in parts of the body other than the lungs), accounts for around 20% of TB burden worldwide. In 2013, informed by a non-Cochrane review, WHO recommended Xpert MTB/RIF (Xpert), a rapid nucleic acid amplification assay, for certain forms of extrapulmonary TB. We performed a Cochrane review to update the literature and address previously noted limitations, including the adequacy of culture as reference standard, given the paucibacillary nature of extrapulmonary TB. We provide an example of our approach for TB meningitis.

**Methods:** To estimate Xpert accuracy, we performed bivariate meta-analysis (standard model) with culture reference standard and then fit a latent class meta-analysis (LCMA) model adding parameters for culture sensitivity and specificity and possible conditional dependence (shared errors) by Xpert and culture. All analyses used Bayesian inference.

**Results:** Xpert pooled sensitivity and specificity were 71.1% and 98.0% (standard model) and 63.1% and 99.6% (LCMA). Culture pooled sensitivity was 68.6% (LCMA). Between-study heterogeneity in Xpert accuracy decreased after adjustment for heterogeneity in culture accuracy.

**Conclusions:** Xpert specificity for TB meningitis was high in all analyses. Xpert sensitivity was close to that of culture. Adjustment in Xpert accuracy by LCMA underscored the relatively low sensitivity of culture and its limitations as a reference standard. Guidance is needed for summarizing results in the Summary of Findings Table when the reference standard appears inadequate.

**Table 1 (abstract P58).** Xpert for TB meningitis, 29 studies (3774 specimens, 433 culture-confirmed TB)

Pooled sensitivity (95% CrI)	Pooled specificity (95% CrI)	Predicted sensitivity (95% CrI)	Predicted specificity (95% CrI)
Xpert accuracy against culture reference, bivariate			
71.1% (60.9, 80.4)	98.0% (97.0, 98.8)	71.1% (27.8, 94.8)	98.0% (88.1, 99.7)
Xpert accuracy, latent class			
63.2% (53.8, 73.6)	99.6% (98.5, 99.9)	63.1% (39.9, 83.0)	99.6% (98.3, 99.9)
Culture accuracy, latent class			
68.6% (59.0, 78.0)	99.3% (98.1, 99.8)	68.5% (44.9, 86.5)	99.3% (97.7, 99.8)

### P59 The calibration slope does not necessarily measure calibration

#### R. J. Stevens^1^ and K. K. Poppe^2,3^

##### ^1^Nuffield Department of Primary Care Health Sciences, University of Oxford; ^2^School of Population Health, University of Auckland; ^3^Department of Medicine, University of Auckland

###### **Correspondence:** R. J. Stevens

**Background**

The slope of a plot of observed against estimated outcomes is a useful validation statistic for prediction models, for example when used as part of a four-part ABCD approach to validation. The slope is often referred to as a calibration slope. While some authors have used “calibration” to mean overall calibration, others have reserved the term “calibration in the large” for overall calibration and used calibration to mean the accuracy of a prediction rule at a more detailed level.

**Objectives**

To review current use and interpretation of the calibration slope, and compare it to behaviour of the calibration slope in practice.

**Methods**

1. We searched for papers published in 2016 and 2017 using the calibration slope, and analysed the text to determine whether authors interpreted it as a measure of calibration, discrimination, or not explicitly as either. 2. We studied the behaviour of the calibration slope first in artificial, examples, and secondly in a re-analysis of a previously published paper.

**Results**

In 40 papers using calibration slope, 30 (75%) interpreted it explicitly as a measure of calibration, 1 interpreted it explicitly as a measure of discrimination, and 9 as neither. Proof-of-concept examples show that the calibration slope can remain constant as calibration varies. In a real example the calibration slope correlates with the c-statistic (r=0.95; p<0.001) but not calibration-in-the-large (r=0.016, p>0.9).

**Conclusions**

Although the calibration slope is useful when used in combination with the intercept, it does not in itself quantify calibration. Many authors inadvertently fail to quantify calibration, by depending on the calibration slope alone. To prevent misunderstanding, and to promote the use of better strategies for prediction model validation, the term “calibration slope” should be retired in favour of a less misleading alternative.

### P60 Sharing and use of biomarker discovery data and computations for research and education; Using R to improve reproducibility of data analyses

#### Marc A. T. Teunis^1^, Jan Willem Lankhaar^2,3^, Eric Schoen^4^, Shirley Kartaram^1^, Raymond Pieters^1,5^

##### ^1^Research Group Innovative Testing in Life Sciences & Chemistry, Research Center for Healthy and Sustainable Living, HU University of Applied Sciences Utrecht, the Netherlands; ^2^Digital Smart Services Research Group, HU University of Applied Sciences Utrecht, the Netherlands; ^3^Institute for ICT, HU University of Applied Sciences Utrecht, the Netherlands; ^4^TNO, Zeist, the Netherlands; ^5^Institute for Risk Assessment Sciences, Utrecht University, the Netherlands

###### **Correspondence:** Marc A. T. Teunis

**Introduction**

Working with big(ger) datasets has become an essential part of Life Sciences research. Due to the existence of a large amount of open data in this field it has become pivotal to use workflows that support the data analysis process in a reproducible way. Here we demonstrate such a workflow. We conclude that the combination of using literate programming, a self-written R-package to contain all data and analyses and the use of Git/Github.com greatly enhances reproducibility and the ability to share and publish the project work.

**Methods**

For our analytics workflow we used the Statistical Programming Language R [1]. In this research project, young adult men were requested to cycle four different training protocols on a bike-ergometer. Rest conditions were used as a control. Before, during and after exercise blood and saliva were collected from the volunteers. Before and after cycling, the volunteers also donated a urine sample. Biological samples were analyzed for a range of biomarkers including hormones, cytokines and blood cells. Furthermore, metabolome and the transcriptome analysis were performed. The main research question addressed in this study was whether we can use a subset of these biomarkers to classify the amount of exercise that was delivered. To answer this question we analyzed the data with multi-level statistical models and supervised machine learning.

**Results**

Due to the data intensive nature of the project and the fact that many laboratories were involved, R was used in all phases of the analytics cycle. We implemented the 7 Guerilla Analytics principles posed by Edna Ridge [2]. These principles help maintaining a link between the original data and the data in a curated and combined dataset and ensure reproducibility of analysis and visualizations.

**Conclusions**

Here we demonstrate that these principles can be implemented in a R-package, thereby contributing to the reproducibility of this research. We demonstrate our machine-learning experiments to illustrate the package.

References

1) R Core Team (2017). R: A language and environment for statistical computing. R Foundation for Statistical Computing, Vienna, Austria. URL https://www.R-project.org/

2) Guerrilla Analytics: A Practical Approach to Working with Data Paperback – 23 Sep 2014. **ISBN-13:** 978-0128002186

3) Hadley Wickham (2017). tidyverse: Easily Install and Load the ‘Tidyverse’. R package version 1.2.1. https://CRAN.R-project.org/package=tidyverse

### P61 A systematic review on *Diagnostic Test Evaluation Methodology*: The evaluation of diagnostic tests in the absence of gold standard

#### Chinyereugo Umemneku^1^, Joy Allen^2^, Kevin Wilson^3^, Luke Vale^1^

##### ^1^Institute of Health & Society, Newcastle University, Newcastle, UK; ^2^NIHR Newcastle In Vitro Diagnostics Co-operative, Newcastle University and Newcastle upon Tyne NHS Foundation Trust, Newcastle, UK; ^3^School of Mathematics, Statistics and Physics, Newcastle University, Newcastle, UK

**Background**:

Diagnostic accuracy studies typically estimate the performance of a new clinical test by comparing it’s results with the best available reference standard. Provided the reference standard used is, or is assumed to be, a “gold standard” and is applicable to all participants of the study, the seemingly unbiased estimates of the accuracy measures, such as sensitivity and specificity can be obtained using the conventional method. However, if the reference standard is imperfect or cannot be applied to all study participants; other methods can be used to estimate diagnostic accuracy to overcome these shortcomings.

**Aim**:

This systematic review seeks to describe methods that have been proposed or applied in evaluations of diagnostic tests in the absence of gold standard. It aims to update the review undertaken 11 years ago by Rutjes et al. (2007) entitled “*Evaluation of diagnostic tests when there is no gold standard. A review of methods”*.

**Methodology**:

A peer reviewed protocol was developed and registered in PROSPERO. Databases related to medical research such as Medline, Embase, Scopus, Web of Knowledge, HMIC, Wiley Online database, CINAHL, PROSPERO were searched. Articles that met the eligibility criteria were included in the review. A PRISMA chart was used to depict the number of articles searched and included in the review.

**Data Analysis:**

Data will be collected from eligible articles using the data collection form developed by the authors. Data extracted from the articles will include: the method applied or proposed, important assumptions, case-studies used, and the strengths and weaknesses of each method. The information obtained will be synthesised qualitatively.

**Conclusion**:

The review will describe novel methods using case studies, articulate the strengths and weaknesses of the methods and develop recommendations for their use.

### P62 Systematic reviews on the prognostic role of biomarkers in heart failure

#### M. Vazquez-Montes^2^, N. Kadoglou^1^, N. Roberts^5^, G. Collins^1^, R. Perera^2^, D. Altman^1^, R. Hobbs^2^, C. Taylor^2^, P. Dhiman^1^, K. Moons^3^, T. DeBray^3^, J. Parissis^4^, M. H. Trivella^1^

##### ^1^Centre for Statistics in Medicine, University of Oxford, Oxford, United Kingdom; ^2^Nuffield Department of Primary Care Health Sciences, University of Oxford, Oxford, United Kingdom; ^3^University Medical Centre (UMC), Utrecht, the Netherlands; ^4^Attikon University General Hospital, Athens, Greece; ^5^Bodleian Health Care Libraries, University of Oxford, Oxford, United Kingdom

###### **Correspondence:** M. Vazquez-Montes

**Background**

Heart failure (HF) is a major public health problem with rising prevalence, especially in elderly. Survival rates for advanced HF patients are worse than those for breast or prostate cancer. Two decades of biomarker research highlighted the prognostic ability of certain markers, and informed the development of new or updated prognostic models. Despite numerous published models and NICE’s recognition of the need for prognosis information, no risk stratification models have been adequately established, nor has the quality of the models and the evidence they present being systematically brought together and tested.

**Objectives**

We hypothesise that HF–related biomarkers may offer an added value to the traditional prognostic factors for HF clinical outcomes, independent of other present co-morbidities. We aim to test this hypothesis through a systematic reviews series assessing the evidence of HF prognostic models using novel meta-analysis (MA) methodology and relevant critical appraisal tools.

**Methods**

We follow Cochrane methodology. Published search filters were combined for a sensitive literature search. Prognostic models including at least one HF-related biomarker were eligible. Independent pairs of co-authors carried out screening and data extraction. Based on the CHARMS and PROBAST checklists we considered model development studies with and without external validation in independent data, and model updating studies. MA will be carried out using recently published novel methodology.

**Results**

Searches yielded over 40,000 titles, highlighting the need for tighter, updated prognostic search filters. A pilot screening of 10% of these (ie 4000) returned only a 2% for full text screening, with an ultimate estimate of 150 included models for evaluation.

**Conclusions**

This is a complex time constrained project with potential to advise on future HF prognostic model design; contribute to improved HF clinical management; apply recently developed MA methodology for combining prognostic model data, and inform the project for developing Cochrane methodology standards of prognostic model reviews.

**Acknowledgements**

Project funded by the British Heart Foundation (grant no. PG/17/49/33099)

**Appendix**

**Planned systematic reviews**

SR1: Characteristics and methodological quality of prognostic models in HF

SR2: Characteristics and methodological quality of studies exploring the added prognostic value of the biomarkers

SR3: Model validation quality and prediction accuracy of prognostic models in HF

SR4: Meta-analysis of the performance of prognostic models externally validated

SR5: Impact assessments of prognostic models in HF

### P63 The incremental value of biomarkers to the Revised Cardiac Risk Index to predict major cardiac events and overall mortality after noncardiac surgery: a systematic review and meta-analysis

#### Lisette M. Vernooij^1,2^, Johanna A. A. G. Damen^2^, Wilton A. van Klei^1^, Karel G. M. Moons^2^, Linda M. Peelen^1,2^

##### ^1^Department of Anesthesiology, University Medical Center Utrecht, Utrecht; ^2^Department of Epidemiology, Julius Center for Health Sciences and Primary Care, University Medical Center Utrecht, Utrecht

###### **Correspondence:** Lisette M. Vernooij

Background

The Revised Cardiac Risk Index (RCRI) is a predictive tool to estimate the postoperative probability of major adverse cardiac events (MACE) in patients undergoing noncardiac surgery. Although commonly used, external validation reveals only moderate performance. Addition of biomarker(s) to the RCRI seems promising.

Objective

In this systematic review, we aim to quantify the added predictive value of several biomarkers to the RCRI, using and developing new methodology for systematic reviews of incremental value studies.

Methods

A systematic search has been conducted in Web of Science for articles citing the original development paper. Inclusion criteria are original research papers reporting external validation of the RCRI with model updating using biomarkers. Updating of the model consists of adding one or multiple biomarkers to the RCRI. The predictive performance of the extended RCRI model is compared to the RCRI alone.

Results

The original RCRI publication has been cited 1,383 times. After screening, 74 articles externally validated the RCRI and of these, 23 articles reported the added value of a biomarker(s) to the RCRI. Blood based biomarkers, including NT-proBNP, BNP, CRP and troponin, were the most frequently studied (n=16). In most articles, only one biomarker was added to the RCRI (n=16). External validation of the RCRI reported in the selected articles resulted in moderate discrimination (c-statistics; median [range]: 0.65 [0.58 - 0.79]), which improved after addition of a biomarker (c-statistics 0.73 [0.59 - 0.88], Table 1). All models improved with addition of a biomarker. CRP and NT-proBNP/BNP yielded the largest increments in terms of model performance (Δ c-statistics were 0.12 and 0.13 respectively). Pooled results will be available at the presentation.

Conclusion

Addition of biomarker(s) to the RCRI resulted in better preoperative risk prediction. Meta-analysis of incremental value studies requires novel methodology.

**Table 1 (abstract P63).** Predictive performance using c-statistic for RCRI alone versus RCRI models with biomarkers

	# of selected articles	RCRI	RCRI + biomarker
*Blood based biomarker*			
NT-proBNP/BNP	6	0.61 [0.59-0.68]	0.74 [0.65-0.77]
Troponin	4	0.66 [0.59-0.72]	0.72 [0.67-0.83]
CRP	3	0.63 [0.59-0.67]	0.75[0.69-0.77]
Other	3	0.69 [0.58-0.79]	0.61 [0.59-0.79]
*Imaging based biomarker*			
Coronary computed tomographic angiography	3	0.63 [0.62-0.65]	0.73 [0.66-0.77]
Other	3	0.65 [0.57-0.78]	0.70 [0.65-0.79]

### P64 The paradox of the normal test in primary care; risk of cancer in patients with normal platelets and inflammatory markers

#### Jessica Watson^1,2^, Sarah Bailey^3^, Luke Mounce^3^, Penny Whiting^2^, Willie Hamilton^3^

##### ^1^Centre for Academic Primary Care, Bristol Medical School, University of Bristol; ^2^CLAHRC West; ^3^University of Exeter Medical School

###### **Correspondence:** Jessica Watson

**Background**

Diagnosis in primary care can be challenging; many early symptoms of cancer are non-specific and low risk. Clinicians may use ‘routine’ blood tests in such patients for reassurance, assuming negative tests represent absence of disease. Diagnosis is a two-step process; the first Bayesian step is the clinicians’ decision to perform a test; the second is the test result itself.

**Objectives**

To determine incidence of cancer in primary care populations with inflammatory marker (C-reactive protein, ESR and plasma viscosity), or platelet tests using primary care records.

**Methods**

Two independent prospective cohort studies of 40,000 and 200,000 UK Primary Care patients using Clinical Practice Research Datalink (CPRD). The primary outcome for both studies was 1-year cancer incidence.

**Results**

For context, NICE recommends urgent cancer investigations or referral for patients with cancer risk of 3% or above. For inflammatory markers those with positive tests had a 1-year cancer incidence (PPV) of 2.80%, test negatives 1.28% and untested 0.84%, the last of these being marginally below expected figures from National Cancer Registry and Analysis Service (NCRAS). For platelets those with positive tests had 1-year cancer incidence of 7.84%, test negatives 2.82%, and population baseline from NCRAS 1.41%. For both tests a significant gender difference was demonstrated; men with normal inflammatory markers have 1.75% 1-year cancer incidence, compared to 0.98% for women; men with normal platelet count have 4.1% 1-year cancer incidence, compared to 2.2% in women.

**Conclusions**

These results demonstrate a clear Bayesian phenomenon in selection of patients for simple testing in primary care. The selection process identifies a group at significantly higher risk, with this additional risk not wholly eliminated by a negative result. This phenomenon demonstrates the need for clinical vigilance with negative test results. We anticipate a similar phenomenon occurs with other test results, and may occur in secondary care.

### P65 PROBAST – A risk of bias tool for prediction modelling studies

#### R. F. Wolff^1^, K. G. M. Moons^2,3^, R. D. Riley^4^, P. F. Whiting^5,6^, M. Westwood^1^, G. S. Collins^7^, J. B. Reitsma^2,3^, J. Kleijnen^1,8^, S. Mallett^9^

##### ^1^Kleijnen Systematic Reviews Ltd, York, United Kingdom; ^2^Julius Center for Health Sciences and Primary Care, University Medical Center Utrecht, Utrecht, The Netherlands; ^3^Cochrane Netherlands, University Medical Center Utrecht, Utrecht, The Netherlands; ^4^Research Institute for Primary Care and Health Sciences, Keele University, Keele, Staffordshire, United Kingdom; ^5^School of Social and Community Medicine, University of Bristol, Bristol, United Kingdom; ^6^The National Institute for Health Research Collaboration for Leadership in Applied Health Research and Care West at University Hospitals, Bristol NHS Foundation Trust, Bristol, United Kingdom; ^7^Centre for Statistics in Medicine, Nuffield Department of Orthopaedics, Rheumatology and Musculoskeletal Diseases, University of Oxford, Oxford, United Kingdom; ^8^School for Public Health and Primary Care (CAPHRI) Maastricht University, Maastricht, The Netherlands; ^9^Institute of Applied Health Sciences, University of Birmingham, United Kingdom

###### **Correspondence:** R. F. Wolff

Background: Quality assessment of included studies is a crucial step in any systematic review (SR). Review and synthesis of prediction modelling studies is an evolving area and a tool facilitating quality assessment for prognostic and diagnostic prediction modelling studies is needed.

Objectives: To introduce PROBAST, a tool for assessing the risk of bias and applicability of prediction modelling studies in a SR.

Methods: A Delphi process, involving 40 experts in the field of prediction research, was used until agreement on the content of the final tool was reached. Existing initiatives in the field of prediction research such as the REMARK and TRIPOD reporting guidelines formed part of the evidence base for the tool development. The scope of PROBAST was determined with consideration of existing tools, such as QUIPS and QUADAS‑2.

Results: After six rounds of the Delphi procedure, a final tool was developed which utilises a domain-based structure supported by signalling questions similar to QUADAS‑2. PROBAST assesses the risk of bias and applicability of prediction modelling studies. Risk of bias refers to any shortcomings in the study design, conduct or analysis leading to systematically distorted estimates of predictive performance or an inadequate model to address the research question. The predictive performance is typically evaluated using calibration, discrimination and sometimes classification measures. Assessment of applicability examines whether the prediction model development or validation study matches the systematic review question in terms of the target population, predictors, or outcomes of interest

PROBAST comprises four domains (Participant selection; Predictors; Outcome; Analysis) and 20 signalling questions grouped within these domains.

Conclusion: PROBAST can be used to assess the quality of prediction modelling studies included in a SR. The presentation will give an overview of the development process and introduce the final tool.

### P66 Assessing the clinical utility of tests and prediction models in diverse settings: a method that recognizes heterogeneity in Net Benefit

#### L. Wynants^1^, R. D. Riley^2^, D. Timmerman^3^, B. Van Calster^1,4^

##### ^1^Department of Development and Regeneration, KU Leuven, Leuven, Belgium; ^2^Research Institute for Primary Care and Health Sciences, Keele University, Keele, UK; ^3^Department of Obstetrics and Gynecology, University Hospitals Leuven, Leuven, Belgium; ^4^Department of Biomedical Data Sciences, Leiden University Medical Center, Leiden, the Netherlands

###### **Correspondence:** L. Wynants

**Background** It is well understood that a test or multivariable prediction model should be validated in multiple studies or centers before introduction to clinical practice. Recently, meta-analytic techniques have been proposed to summarize discrimination and calibration across populations. Although measures of clinical utility are advocated to go beyond discrimination and calibration and into account the harms of false positives and benefits of true positives, methods that recognize heterogeneity do not yet exist.

**Objectives** To propose a suitable meta-analysis method for Net Benefit (NB).

**Methods** A Bayesian trivariate random-effects meta-analysis of sensitivity, specificity, and prevalence. Across a range of harm-to-benefit ratios, this provides a summary measure of NB, a prediction interval, and an estimate of the probability that the test/model is clinically useful in a new setting. In addition, statistics can be calculated conditional on the known prevalence in a new setting. The proposed methods are illustrated by two case studies: one on the meta-analysis of studies on ear thermometry to diagnose fever, and one on the validation of the LR2 risk model for the diagnosis of ovarian cancer in a multicenter dataset.

**Results** In both case studies the clinical utility of the test/model was heterogeneous, limiting usefulness in practice. Clinical utility depended on the harm-to-benefit ratio dictated by the clinical context. E.g., the probability of usefulness of ear thermometer in a new setting varied from nearly 0 to >.9, depending on the harm-to-benefit ratio. For a given harm-to-benefit ratio, added clinical usefulness of the model over default strategies ‘treat all’ and ‘treat none’ varied with prevalence, reflecting miscalibration and spectrum bias. E.g., the probability of utility of LR2 was .75 in a new setting with prevalence .35 and 1.00 in a new setting with prevalence .15.

**Conclusion** Heterogeneity in clinical utility should be assessed before a test/model is routinely implemented.

### P67 An overview of methods for early health technology assessment

#### Yaling Yang, Lucy Abel, Gail Hayward, Rafael Perera

##### Nuffield Department of Primary Care Health Science, University of Oxford. Radcliffe Primary Care building, Radcliffe Observatory Quarter, Oxford, Ox2 5GG

###### **Correspondence:** Yaling Yang (yaling.yang@phc.ox.ac.uk)

**Background**: Recent years have witnessed the development of Health Technology Assessment (HTA) methods for use by developers and public bodies to assess potential cost-effectiveness at the early stages of device development.

**Objectives:** 1) To provide an overview of current methods used; and 2) To identify issues and needs for future key methodological development in early health technology assessment.

**Methods:** Rapid review methods will be used to identify published methods papers and literature reviews related to early HTA by searching relevant electronic databases including MEDLINE, EMBASE, The National Health Services Economic evaluation database (NHS EED), the Cochrane library, and Econlit. Contacts will be made with research groups who have published early HTA work in both the UK and the Netherlands to identify relevant unpublished papers. Inclusion criteria will be research and review papers that report early HTA methods, as well as commentaries describing or discussing early HTA methods, published in English.

The overview will extract data from papers to answer the below questions:

1. How ‘early HTA’ was defined, especially what stage of device development means ‘early’?
2. What are the proposed aims of early HTA?
3. What frameworks have been developed to evaluate early HTA?
4. What methods have been proposed/identified/applied in early HTA?
5. What are the extant methodological issues in need of further examination and development?

**Results:** The literature review is currently ongoing and results will be ready at the time of conference in early July 2018. Initial search found four literature reviews, and five methodology development papers. Preliminary findings include that papers discussed differences between early and late stage HTA and associated methods applied. These include headroom analysis, decision analytical modelling, sensitivity analysis, Bayesian modelling, and probabilistic risk analysis. Discussions were limited on methods to identify clinical care pathways.

### P68 Using joint models to include repeated measurements of HbA1c for the prediction of nephropathy in people with type 2 diabetes

#### J. S. Yauw^1^, A. A. van der Heijden^2^, J. W. Beulens^1,3^, P. J. M. Elders^2^, T. L. Feenstra^4,5^, R. M. C. Herings^6^, K. G. M. Moons^1^, G. Nijpels^2^, L. M. Peelen^1^

##### ^1^UMC Utrecht Julius Center, Utrecht University, Utrecht, the Netherlands; ^2^Department of General Practice and Elderly Care Medicine, Amsterdam Public Health research institute, VU University Medical Centre, Amsterdam, The Netherlands; ^3^Department of Epidemiology & Biostatistics, VU University Medical Centre, Amsterdam, The Netherlands; ^4^National Institute for Public Health and the Environment, Department for Prevention and Health Services Research, The Netherlands; ^5^Groningen University, University Medical Center, Groningen, The Netherlands; ^6^PHARMO Institute for Drug outcomes Research, Utrecht, The Netherlands

###### **Correspondence:** J. S. Yauw

**Background**

Prediction models for people with type 2 diabetes are often static models, using only a person’s risk profile at a single time point. As the cumulative amount of HbA1c (‘glycemic burden’) was found to be associated with various diabetes outcomes, including information on dynamics of HbA1c over time may increase the accuracy of predicting nephropathy over using a single HbA1c measurement only.

**Objectives**

To compare a ’static’ prediction model based on Cox regression analysis to a joint modelling approach for the prediction of diabetic nephropathy, using a single or repeated HbA1c measurements respectively.

**Methods**

This study included 7616 people with type 2 diabetes from the Hoorn Diabetes Care System cohort, who were followed annually from 1998 onwards. Nephropathy was defined as macroalbuminuria. For the Cox regression only the baseline HbA1c value was taken into account. For the joint model, repeated measurements of HbA1c were used. In both models, baseline variables sex, age, diabetes duration, systolic blood pressure, BMI, triglycerides and total cholesterol were included as other predictors. All variables were standardized before joint model analysis. Results were expressed in terms of hazard ratios and Harrell’s C statistic for discrimination.

**Results**

In total, 394 (5.3%) people developed nephropathy during a mean follow-up of 6.3 (±4.9) years. In both models, sex, age, systolic blood pressure, BMI, triglycerides and HbA1c were independent predictors of diabetic nephropathy. Specifically, the hazard ratio for HbA1c was equal to 1.14 (95% CI 1.07-1.22) in the Cox model and 1.84 (1.61-2.10) using joint modelling. The joint model showed an AUC of 0.70 and a prediction error of 0.06. The discriminative ability of the Cox model was slightly lower, with an AUC of 0.68.

**Conclusion**

Based on model discrimination, including repeated HbA1c measurements seems to predict the risk of diabetic nephropathy slightly better compared to the Cox model.

### P69 Adaptive designs for diagnostic accuracy studies

#### Antonia Zapf

##### Department of Medical Biometry and Epidemiology, University Medical Center Hamburg-Eppendorf, Hamburg, Germany

Planning a clinical trial is always tainted with uncertainty. On the one hand for the sample size calculation assumptions are necessary, which prove to be false during the ongoing trial. On the other hand it can be of interest to modify design aspects during the study (whereas these modifications have to be pre-specified in the study protocol). While for treatment studies there are plenty of methods for such adaptive study designs, for diagnostic accuracy studies there are almost no methods for adaptive designs. Accordingly, no diagnostic accuracy trials with an adaptive design could be found in the literature. Since diagnostic trials fail very often because of wrong assumptions, it is highly necessary to develop methods for adaptive designs in the field of diagnostic trials. An example is the recently published diagnostic trial from Waugh et al. [1].

In the talk I will present different settings where adaptations would be helpful in diagnostic trials and I will distinguish between blinded and unblinded sample size re-estimation. Furthermore, drawing from the literature on adaptive designs in the field of treatment studies, I will show where existing methods can be transferred to diagnostic trials [2,3]. Regarding the remaining blind spots, I will present existing and new methods specific for diagnostic trials [4].

**References**

1. Waugh et al. (2017). Spot protein–creatinine ratio and spot albumin–creatinine ratio in the assessment of pre-eclampsia. Health Technology Assessment, 21(61):1-90.

2. Asakura et al. (2017). Interim evaluation of futility in clinical trials with co‐primary endpoints. Talk on the CEN‐ISBS, Vienna.

3. Sander et al. (2017). Blinded sample size recalculation in clinical trials with binary composite endpoints. J Biopharm Stat. 2017;27(4):705‐715.

4. McCray et al. (2017). Sample size re‐estimation in paired comparative diagnostic accuracy studies with a binary response. BMC Med Res Metthodol, 17:102.

### P70 Confronting uncertainties in prognosis: Statistics should support an approach to clinical decision-making based on “Prepare for the worst. Hope for the best. Bet according to the odds”

#### M Power^1^, BC Lendrem^2^, AJ Allen^2^, T Fanshawe^3^, J Simpson^2^

##### ^1^NIHR Newcastle In Vitro Diagnostics Co-operative, Newcastle upon Tyne Hospitals Foundation Trust, Newcastle, UK; ^2^NIHR Newcastle In Vitro Diagnostics Co-operative, Newcastle University, Newcastle, UK; ^3^NIHR Community Healthcare MIC, University of Oxford, Oxford, UK

###### **Correspondence:** BC Lendrem

**Background**

Statisticians tend to provide, clinicians to communicate, and patients to understand prognosis in terms of a single number, as if it were a date in a calendar. Patients find information about prognosis difficult to understand and of little use for decision making.

**Objectives**

To develop an evidence-based approach to improving the utility of prognostic information.

**Methods**

We synthesized key literature on presenting and using prognosis information, and developed a prototype for visualizing prognostic statistics and confronting the inherent uncertainties, using ovarian cancer survival data from SEER as an example.

**Results**

Quantifying prognosis

Can be expressed as:

1. Life expectancy (e.g. one year).

2. Chance of living one year (e.g. 50%).

Uses for prognostic statistics:

1. For groups: e.g. to compare outcomes and choose the most likely outcome.

2. For individuals: supporting an approach of planning for the worst, hoping for the best, and acting on the best bet.

Quantifying uncertainty

Uncertainty in prognosis is due to:

1. Stochastic processes – can be quantified by confidence intervals and inter-centile ranges, and visualized with histograms and probability distributions.

2. Changing clinical information (for example surviving one year increases your probability of survival) – can be quantified and visualized by conditional prognosis plots. Prognostic information intended for individuals seldom includes information on uncertainties. Some statisticians have recommended routinely providing inter-centile ranges, and others conditional prognosis plots. However, reporting guidelines for prognosis studies have not adopted these recommendations.

**Conclusion**

We propose a programme of work:

1. Studies of how patients would like the uncertainties in prognosis to be presented, and how they would use the information.

2. Updates of reporting guidelines for prognosis studies to include information on uncertainties and conditional prognosis (where practical).

3. Development of a database-building toolkit for conditions with prognostic information, to help patients and physicians.

### P71 Accounting for time-varying treatments when developing a prognostic model: a case study in patients treated with beta-blockers

#### R Pajouheshnia^1^, NA Schuster^1^, KGM Moons^1,2^, FH Rutten^1^, RHH Groenwold^3^, LM Peelen^1^

##### ^1^Julius Center for Health Sciences and Primary Care, University Medical Center Utrecht, the Netherlands; ^2^Cochrane Netherlands, University Medical Center Utrecht, the Netherlands; ^3^Department of Clinical Epidemiology, Leiden University Medical Centre, Leiden, the Netherlands

###### **Correspondence:** R Pajouheshnia

**Background**

For a prognostic model to be used to guide treatment decisions, predictions should represent a patient’s outcome risk if they were to remain untreated. Clinical data used for model development typically include some individuals who were treated during follow-up. Ignoring this can lead to a model that underestimates the true untreated outcome risk.

**Objectives**

To compare methods to account for treatment use during follow-up when developing a prognostic model.

**Methods**

A prognostic (Cox) model to predict 5-year mortality risk without using selective beta-blockers was developed using the electronic health record data of 1905 patients (585 deaths). We compared 5 methods to account for selective-beta blocker use during follow-up: (i) excluding treated individuals, (ii) censoring treated individuals, (iii) inverse probability of censoring weighting after censoring treated individuals, (iv) including treatment as a binary covariate in the model and (v) including treatment use as a time-varying covariate in the model. The comparisons were repeated in a highly-treated patient subset and with a simplified prognostic model.

**Results**

324 (17%) patients began using selective beta-blockers during follow-up. The coefficients of the prediction models varied according to each modelling method. Excluding treated individuals resulted in a model that provided, on average, slightly higher predictions compared to a model that ignored treatment. However, these differences did not translate to substantial differences in predictive performance (c-statistic, calibration slope, Brier score) in any of the analyses.

**Conclusion**

Treatment hardly affected predictive performance in our case study. Despite theoretical advantages of certain methods to account for treatment use, in practice the actual benefit of applying these methods may be small. Further case studies and simulations are needed to investigate when it is necessary to take into account the effect of treatment when developing a prognostic model.

### P72 Clinical prediction models for patients with early rheumatoid arthritis: A systematic review and evidence synthesis

#### J Hamilton, R. Archer, JW Stevens, M Stevenson, E Hock, M Clowes^1^, M Essat, E Poku, A Pandor

##### Health Economics and Decision Science (HEDS), School of Health and Related Research (ScHARR), University of Sheffield, United Kingdom

###### **Correspondence:** J Hamilton

**Objectives**

To systematically review the evidence and assess the relative performance of clinical prediction models in the evaluation of patients with early rheumatoid arthritis.

**Methods**

A systematic review of studies describing the development, external validation and impact of eligible clinical prediction models was conducted in accordance with PRISMA guidelines and current best practice for undertaking prognostic reviews. Data on predictive performance were described in a narrative synthesis, presented separately for internal and external validation studies. Evidence synthesis using meta-analysis was considered for external validation studies.

**Results**

Twenty-two model development studies and one combined development/external validation study reporting 39 clinical prediction models for three relevant outcomes were included. Five external validation studies evaluating eight models for radiographic joint damage were also included. C statistics for radiographic progression outcomes (different definitions) ranged between 0.63 and 0.87 (n=8) and between 0.78 and 0.82 for Health Assessment Questionnaire (HAQ) outcomes (n=2). For models that had been externally validated, predictive performance varied considerably, suggesting unexplained heterogeneity in the populations in which the models are being tested. Three models (ASPIRE-CRP, ASPIRE-ESR, BeSt) were validated using the same outcome definition in two external populations. The random effects meta-analysis suggested the most favourable performance across external validations was for BeSt (C statistic 0.72, 95% CI: 0.20, 0.96). However, for all models, there is substantial uncertainty in the expected predictive performance in a new sample of patients, indicating that we cannot be confident that the performance of the models is better than would be expected by chance.

**Conclusion**

Meta-analysis was limited by the small number of external validation studies and the results do not provide a definitive conclusion about performance of the models in future studies. Reasons for the heterogeneity in performance could not be explored. Uncertainty remains over the optimal prediction model(s) for use in clinical practice.

## Oral Presentations

### O1 Pragmatic versus explanatory diagnostic accuracy studies

#### Patrick M. Bossuyt^1^, Maria Olsen^1^, Chris Hyde^2^, Jérémie F. Cohen^3^

##### ^1^Department of Clinical Epidemiology, Biostatistics and Bioinformatics, Amsterdam Public Health, Academic Medical Center, Amsterdam; ^2^Peninsula Technology Assessment Group, University of Exeter, Exeter; ^3^Inserm UMR 1153 Obstetrical Perinatal and Pediatric Epidemiology Research Team, Center for Epidemiology and Statistics Sorbonne Paris Cité, Paris Descartes University, Paris

###### **Correspondence:** Patrick M. Bossuyt

Some fifty years ago, Schwartz and Lelouch discussed how therapeutic trials can try to resolve two very different problems. The first set of so-called *explanatory trials* aims at understanding a treatment, seeking to discover whether it causes benefit and/or if difference exists between two treatments. The second set of *pragmatic trials* aims at decision-making: these trials try to answer the question which treatment is preferable under usual clinical circumstances. The difference affects the definition of the treatments, the choice of study participants, and the way in which the treatments are compared. The PRagmatic Explanatory Continuum Indicator Summary (PRECIS-2) was later developed to further clarify this distinction.

Diagnostic accuracy studies evaluate the performance of a test in correctly identifying those with and without the correct target condition, by comparing index test results with those of the clinical reference standard.

We argue that, like therapeutic trials, diagnostic accuracy studies can also try to answer two different questions. One set, *explanatory accuracy studie,* aims at understanding how different conditions affect the distribution of test results. A second set, aimed at decision-making, evaluates the consequences of relying on the test’s results for clinical management: *pragmatic accuracy studies*. Confusingly, both types of trials often present their findings in terms of sensitivity and specificity or the area under the ROC curve.

The difference between *pragmatic* and *explanatory* diagnostic accuracy studies cannot be simplified as a matter of design-related bias and applicability. It has implications for the definition of the index test, the eligibility criteria and recruitment of study participants, the choice of the study outcomes, the analysis of results, and the interpretation. We present the building blocks for PRECIS-DTA, at tool that can be used in the design, analysis, interpretation, reporting and communication of diagnostic accuracy studies.

### O2 Empirical evidence on the impact of study characteristics on the performance of prognostic models: a meta-epidemiological study

#### J. A. A. G. Damen^1,2^, T. P. A. Debray^1,2^, R. Pajouheshnia^1^, J. B. Reitsma^1,2^, R. J. P. M. Scholten^1,2^, K. G. M. Moons^1,2^, L. Hooft^1,2^

##### ^1^Julius Center for Health Sciences and Primary Care, University Medical Center Utrecht, Utrecht University, Utrecht, The Netherlands; ^2^Cochrane Netherlands, Utrecht, The Netherlands

###### **Correspondence:** J. A. A. G. Damen

**Background:** Meta-epidemiological studies have shown that shortcomings in study design can lead to biased estimates of treatment effects and diagnostic test accuracy. It remains unclear to what extent study characteristics may affect estimates of prognostic model performance.

**Objectives:** To assess the relation between study characteristics and the results of external validation studies of prognostic models.

**Methods:** We searched electronic databases for systematic reviews of prognostic models. Reviews from non-overlapping clinical fields were selected if they reported common performance measures (concordance (c)-statistic or ratio of observed over expected number of events (OE ratio)) from ten or more validations of the same model. From the included validation studies we extracted study design features, population characteristics, methods of predictor and outcome assessment, and the aforementioned performance measures. Random effects meta-regression was used to quantify the association between study characteristics and model performance.

**Results:** We included ten reviews, describing a total of 224 validations. Associations between study characteristics and model performance were heterogeneous across reviews. C-statistics were most associated with population characteristics and measurement of predictors and outcomes, e.g. validation in a continent different from the development study resulted in a higher c-statistic, compared to validation in the same continent (difference in logit c-statistic 0.10 [95% CI 0.04, 0.16]), and validations with eligibility criteria comparable to the development study were associated with higher c-statistics compared to narrower criteria (difference in logit c-statistic 0.21 [95% CI 0.07, 0.35]). Using a case-control design was associated with higher OE ratios, compared to using cohort data (difference in log OE ratio 0.97 [95% CI 0.38, 1.55]).

**Conclusion:** Variation in performance of prognostic models appears mainly associated with variation in case-mix, study design, and predictor and outcome measurement methods. Researchers validating prognostic models should carefully take these study characteristics into account when interpreting the achieved performance of prognostic models.

### O3 A framework for meta-analysis of prediction model studies with binary and time-to-event outcomes

#### T. P. A. Debray^1^, J. A. A. G. Damen^1^, R. D. Riley^2^, K. Snell^2^, J. B. Reitsma^1^, L. Hooft^1^, G. S. Collins^3^, K. G. M. Moons^1^

##### ^1^Julius Center for Health Sciences and Primary Care, Cochrane Netherlands, University Medical Center Utrecht, Utrecht University, Utrecht, the Netherlands; ^2^Research Institute for Primary Care and Health Sciences, Keele University, Staffordshire, United Kingdom; ^3^ University of Oxford, Oxford, United Kingdom

###### **Correspondence:** T. P. A. Debray

Background: It is widely recommended that any developed - diagnostic or prognostic - prediction model is externally validated in terms of its predictive performance measured by calibration and discrimination. When multiple validations have been performed, a systematic review followed by a formal meta-analysis helps to summarize overall performance across multiple settings, and reveals under which circumstances the model performs suboptimal (alternative poorer) and may need adjustment.

Objectives: To discuss how to undertake meta-analysis of the performance of prediction models with either a binary or a time-to-event outcome.

Methods: We address how to deal with incomplete availability of study-specific results (performance estimates and their precision), and how to produce summary estimates of the c-statistic, the observed:expected ratio and the calibration slope. Furthermore, we discuss the implementation of frequentist and Bayesian meta-analysis methods, and propose novel empirically based prior distributions to improve estimation of between-study heterogeneity in small samples. Finally, we illustrate all methods using two examples: meta-analysis of the predictive performance of EuroSCORE II and of the Framingham Risk Score. All examples and meta-analysis models have been implemented in our newly developed R package *metamisc*.

Results: Information on model discrimination and calibration was often incomplete, but could be restored for most studies. Although the proposed meta-analysis models yielded similar summary estimates, the Bayesian approach allows for more accurate estimation of between-study heterogeneity when few studies are included in the meta-analysis.

Conclusion: Meta-analysis of prediction models is a feasible strategy despite the complex nature of corresponding studies. As developed prediction models are being validated increasingly often, and as the reporting quality is steadily improving, we anticipate that evidence synthesis of prediction model studies will become more commonplace in the near future. The R package *metamisc* is designed to facilitate this endeavor, and will be updated as new methods become available.

### O4 Are Cochrane reviews of diagnostic test accuracy informing clinical guidelines?

#### B. Harris^1^, S. Beese^1^, J. O’Rourke^1^, E. Carter^2^, C. Davenport^1,3^, S. Mallett^1,3^, Y. Takwoingi^1,3^, A. Eisinga^2^, J. Deeks ^1,3^

##### ^1^Test Evaluation Research Group, Institute of Applied Health Sciences, University of Birmingham, Edgbaston, Birmingham B15 2TT, UK; ^2^Cochrane UK, Summertown Pavilion, 18-24 Middle Way, Oxford, OX2 7LG, UK; ^3^NIHR Birmingham Inflammation Biomedical Research Centre, University of Birmingham, Edgbaston, Birmingham B15 2TT, UK

###### **Correspondence:** B. Harris; J. Deeks

**Background:** Cochrane has been publishing Diagnostic Test Accuracy (DTA) reviews for 10 years, and close to publishing their 100^th^ DTA review. The methods and reporting of Cochrane DTA reviews were designed to ensure they address patient management questions by providing evidence summaries suitable for incorporation in clinical guidelines.

**Objectives:** To assess the extent to which Cochrane DTA reviews have been incorporated in clinical guidelines; identify which guideline developers and topics are most likely to make use of Cochrane DTA evidence; note key features of reviews most cited.

**Methods**: Cochrane UK tracks citations of Cochrane Reviews in clinical guidelines published worldwide by searching online, open access sources of accredited guidelines (free at the point of use) for the word “Cochrane”. We analysed citations of Cochrane DTA reviews, identified up until the end of March 2018, by guideline developer, topic and key characteristics of reviews, including: (a) focus on comparative accuracy questions; (b) number of studies included; (c) clear positioning of the test in the clinical pathway; (d) presentation of consequences of testing; (e) clear clinical recommendations.

**Results:** As of 2nd March 2018, 41 of 92 published DTA reviews (45%) from 17 Cochrane Review Groups have been used to inform 56 clinical guidelines, 14 have been used in more than one guideline. DTA reviews have been cited in UK guidelines (30 times, 19 by NICE); German (8); US (5); WHO (4); Canada (3); Europe (3); World, Australia and Belgian (2); Ireland, Poland, Spain, India, South Africa, Latin America, Italy, and France (1).

**Conclusions:** Many Cochrane DTA reviews are impacting on clinical guidelines. Cochrane DTA reviews are resource intensive and it is important that the topics chosen, and methods used optimise

### O5 Defining methods to evaluate IVDs for WHO’s new Essential Diagnostics List

#### J. Deeks^1,2^, Y. Takwoingi^1,2^, S. Mallett^1,2^

##### ^1^Test Evaluation Research Group, Institute of Applied Health Sciences, University of Birmingham, Edgbaston, Birmingham B15 2TT, United Kingdom; ^2^NIHR Birmingham Inflammation Biomedical Research Centre, University of Birmingham, Edgbaston, Birmingham B15 2TT, United Kingdom

###### **Correspondence:** J. Deeks

**Background**

In April 2018 WHO held the first meeting of the Strategic Advisory Group of Experts in In Vitro Diagnostics (SAGE IVD) to define the methods that will be used to create a Model List of Essential in vitro Diagnostics (EDL). The EDL intends to provide evidence-based guidance, and set a reference for the development of national lists of essential IVDs. The initial EDL meeting looked at existing WHO guideline recommendations for tests for TB, HIV, hepatitis B and C, malaria and syphilis.

**Objective**

To identify key methodological challenges for WHO evidence review methods to support the EDL process.

**Methods**

Full reports for tests for TB, HIV, hepatitis B and C, malaria and syphilis were identified and methods and reporting compared against a 20-item framework developed from PRISMA-DTA, the Cochrane DTA Handbook, GRADE guidance and discussions of experts. One of three DTA experts reviewed guidance and noted both good practice and notable differences.

**Results**

Nine evaluations for TB, 8 for hepatitis B and C, 1 each for malaria and syphilis, and 16 for HIV were identified and reviewed. Methods and themes identified where harmonisation is required include: abandoning the PICO question to one suited to test accuracy; emphasis on comparative accuracy; the value of protocols; the role of indirect evidence; assessment of risk of bias and applicability; use of existing systematic review evidence; statistical methods; reporting consequences of test use for accuracy evidence; evaluation of evidence beyond accuracy; grading and assessing the strength of evidence. We will illustrate key issues with examples.

**Discussion**

Standardisation of WHO evidence review methods for tests is needed to support development of the EDL. We will report the progress of the SAGE IVD in deciding on a methodological approach for, and report on the key outstanding methodological areas which require further development and research.

### O6 Harnessing individual participant trial data alongside electronic health records to evaluate the potential of precision medicine: application to type 2 diabetes drug therapy

#### John Dennis^1^, William Henley^1^, Angus Jones^2^, Andrew Hattersley^2^, Beverley Shields^2^ on behalf of the MASTERMIND Consortium

##### ^1^Health Statistics Group, University of Exeter Medical School, Exeter, UK; ^2^National Institute for Health Research Exeter Clinical Research Facility, University of Exeter Medical School, Exeter, UK

###### **Correspondence:** John Dennis

**Background:** Individual participant data from randomised trials are increasingly available for researchers to answer secondary research questions. Repositories include YODA and Clinical Study Data Request. There may be great potential to harness these data to evaluate potential precision medicine approaches. We propose a framework involving discovery analysis in routine clinical data followed by validation in trials, and apply this to evaluate a precision medicine approach to predict good response to type 2 diabetes drug therapy.

**Methods:** Discovery analysis: We included 30,511 patients with type 2 diabetes starting either a SGLT-2 inhibitor(SGLT2i) or DPP4-inhibitor(DPP4i) in routine clinical data from the UK (CPRD). Associations between clinical measures and glycaemic response (6 month HbA1c–baseline HbA1c) to each drug were evaluated individually using linear regression. Validation: From YODA, we pooled individual participant data from 6 randomised drug efficacy trials of SGLT2i, 2 had a DPP4i comparator arm (n=3929). In the pooled trial data we tested clinically relevant associations observed in CPRD using multivariable three-level (trial-patient-study visit) linear mixed-effects models.

**Results:** In CPRD, we identified key clinical features associated with differential response to the two drugs (Table 1). Higher baseline HbA1c was associated with greater response (a reduction in HbA1c) to both drugs, but to a greater extent with SGLT2i. Greater SGLT2i response was associated with higher eGFR and lower HDL. Greater DPP4i response was associated with lower triglycerides and lower BMI. All associations replicated in trial data (Table 1).

**Conclusion:** The availability of individual trial data from repositories such as YODA and Clinical Study Data Request provides a tremendous opportunity to evaluate potential precision medicine approaches. Discovery in routine data followed by validation in trial data provides a principled framework to utilise trial data without data-mining. Our findings using this framework suggest there may be potential to develop prediction models for drug response in type 2 diabetes.

**Table 1 (abstract O6).** Change in HbA1c in mmol/mol at 6 months (with 95% confidence intervals) per standard deviation greater baseline value of predictor. A negative value represents a reduction (improvement) in HbA1c

	a) SGLT-inhibitors			b) DPP4-inhibitors		
	Baseline HbA1c	eGFR	HDL	Baseline HbA1c	BMI	Triglycerides
CPRD	-9.5 (-9.8;-9.0)	-1.75 (-2.2;-1.3)	2.2 (1.5;2.9)	-8.4 (-8.6;-8.2)	1.0 (0.8;1.1)	0.9 (0.6;1.1)
Trials	-5.8 (-6.1;-5.5)	-1 (-1.3;0.7)	0.6 (0.3;0.8)	-4.7 (-5.5;-3.9)	0.4 (0.0;0.9)	0.5 (0.1;1.0)

### O7 Tailoring prediction models for use in new settings: Individual participant data meta-analysis for ranking model recalibration methods

#### J. Ensor^1^, E. C. Martin^2^, K. I. E. Snell^1^, T. P. A. Debray^3,4^, M. A. Mamas^1,5^, K. G. M. Moons^3,4^, R. D. Riley^1^

##### ^1^Centre for Prognosis Research, Research Institute for Primary Care and Health Sciences, Keele University, Keele, UK; ^2^Biostatistics Research Group, Centre for Medicine, University of Leicester, Leicester, UK; ^3^Julius Center for Health Sciences and Primary Care, University Medical Center Utrecht, Utrecht, The Netherlands; ^4^Cochrane Netherlands, Julius Center for Health Sciences and Primary Care, University Medical Center Utrecht, Utrecht, The Netherlands; ^5^Department of Cardiology, Royal Stoke University Hospital, Stoke-on-Trent, UK

###### **Correspondence:** J. Ensor

**Background:** The availability of individual participant data (IPD) from multiple sources allows the external validation of a prediction model across multiple settings and populations. When applying an existing prediction model in a new population it is likely that it will suffer from some over or under fitting, potentially causing poor predictive performance. However, rather than discarding the model outright, it may be possible to modify components of the model to improve its performance using model recalibration methods. Here, we consider how IPD meta-analysis methods can be used to compare and select the most appropriate recalibration method, or whether a completely new model is warranted in a particular setting.

**Methods:** We examine four methods for recalibrating an existing logistic prediction model in cardiovascular disease across multiple centres: (i) re-estimation of the intercept, (ii) adjustment of the linear predictor as a whole (calibration slope), (iii) adjustment of individual heterogeneous predictor effects, and finally (iv) re-estimation of all model parameters. We use multivariate IPD meta-analysis to jointly synthesise calibration and discrimination performance across centres for each of the methods. The most appropriate recalibration method can then be evaluated based on the joint probability of achieving a given model performance in a new setting, using this to rank recalibration methods.

**Results:** We present a new Stata package allowing estimation of the joint probability of achieving a set level of model performance in a new setting for each recalibration method, therefore easily identifying the method with the highest probability. We show that the best recalibration method is case specific and promote the use of recalibration as opposed to developing new models unnecessarily when the probability of improved performance through recalibration is high.

**Conclusions:** Multivariate meta-analysis allows quantification of the most appropriate recalibration methods to improve the performance of an existing prediction model in new settings.

### O8 Risk model based stratified patient management of cardiac chest pain versus uniform “non-invasive first” strategies: A summary of short term findings from the CE-MARC2 randomised trial

#### Colin C. Everett^1^, Julia M. Brown^1^, Catherine Reynolds^1^, Catherine Fernandez^1^, Gerry P. McCann^2^, Colin Berry^3^, Petra Bijsterveld^4^, Deborah D. Stocken^1^, Linda D. Sharples^5^, Sven Plein^4^, John P. Greenwood^4^

##### ^1^Clinical Trials Research Unit, Leeds Institute for Clinical Trials Research, University of Leeds, UK; ^2^Department of Cardiovascular Studies, University of Leicester, Clinical Sciences Wing, Glenfield General Hospital Leicester, UK; ^3^Institute of Cardiovascular & Medical Science, University of Glasgow, Glasgow, UK; ^4^Multidisciplinary Cardiovascular Research Centre (MCRC) & Leeds Institute of Cardiovascular and Metabolic Medicine, University of Leeds, Leeds, UK; ^5^London School of Hygiene and Tropical Medicine, London, UK

###### **Correspondence:** Colin C. Everett

**Background**: Many non-invasive diagnostic imaging methods are available to manage secondary care of patients presenting with stable (suspected cardiac) chest pain, though confirmatory diagnosis is often by invasive coronary angiography (ICA). The 2010 NICE guideline for managing chest pain (CG95) recommended risk-stratified management of these patients in Rapid Access Chest Pain clinics. Stratification was by computed pre-test likelihood (PTL) of coronary artery disease (CAD), based on the Duke Clinical Risk (1993) PTL model: patients with PTL>60% were recommended for immediate ICA; patients with PTL 10-60% were recommended for non-invasive imaging with abnormal findings being a “gatekeeper” to ICA referral. Concerns were raised that this PTL-stratified management would lead to an excess of patients undergoing ICA when CAD was not present.

**Objectives:** The UK CE-MARC2 trial (2016) aimed to determine the difference in rates of unnecessary (CAD absent) ICA within 12 months between a NICE CG95 (2010) based risk-stratified management algorithm, or a uniform non-invasive imaging “gatekeeping” strategy either by Cardiac Magnetic Resonance (CMR) or Myocardial Perfusion Scintigraphy (MPS).

**Methods**: 3-arm, parallel group, six-centre Randomised Controlled Trial, 481:481:240 patients were allocated to CMR:MPS:CG95. Endpoints were (primary) unnecessary angiography 12 months post-randomisation and (secondary) major adverse cardiovascular event (MACE) within 12 and 36 months and CAD detection within 12 months.

**Results**: Unnecessary angiography rates in the non-invasive-first strategies were one quarter that in the risk-based CG95 strategy (7.5 and 7.1% vs 28.8%). This difference (21.5%) was due to PTL over-estimation by the Duke model in CG95 patients: only 28% of CG95 mandatory ICA (PTL>60%) detected CAD. There were no significant differences in rates of CAD detection or MACE at 12 months.

**Conclusion**: CE-MARC2 warns that while risk-stratification is a reasonable approach to management, the clinical benefits may be lost if the underlying risk model is poorly calibrated: contemporary external validation is essential.

### O9 An interactive web application to aid diagnostic test accuracy meta-analysis

#### Suzanne Freeman^1^, Clareece Kerby^2^, Nicola Cooper^1^ and Alex Sutton^1^

##### ^1^NIHR Complex Reviews Support Unit, Biostatistics Research Group, Department of Health Sciences, University of Leicester, Leicester, UK; ^2^Biostatistics Research Group, Department of Health Sciences, University of Leicester, Leicester, UK

###### **Correspondence:** Suzanne Freeman

**Background**: A meta-analysis of diagnostic test accuracy (DTA) studies synthesises multiple studies to evaluate the performance of a diagnostic test. Often there is variation between studies surrounding the threshold used to determine whether a patient is healthy. DTA meta-analysis can be performed using either the bivariate or hierarchical summary receiver operating curve (ROC) models, and the results presented either around a mean point or as a summary ROC curve. Conventionally, summary ROC curves are published as static graphs. An alternative is to consider interactive graphs which can allow multiple perspectives to be displayed, information to be tailored to the user’s preference and provide a useful tool to aid sensitivity analyses.

**Objectives**: (i) To develop a freely-available web-based “point and click” interactive tool which allows users to input their data and conduct meta-analyses, including sensitivity analyses. (ii) To illustrate the benefits of the interactive application using an existing DTA meta-analysis (1).

**Methods**: To create our online freely-available interactive application we used the existing R packages mada and Shiny to analyse the data and create an interactive user interface.

**Results**: An interactive online application was created for conducting meta-analysis of DTA studies. The user interface was designed to be easy to navigate. Benefits include the ability for users to enter their own data, exploration of a range of different threshold values and what this means in terms of true and false positive rates, and the ability to conduct sensitivity analyses.

**Conclusion**: We built a freely-available interactive online application, available at https://crsu.shinyapps.io/dta_ma/, which meta-analyses DTA studies, plots the summary ROC curve and allows for sensitivity analyses to be conducted in a timely manner. This application will allow a wide range of users to carry out specialised analyses without needing software beyond an internet browser.

References

1. Kriston L et al *Ann Intern Med* 2008;149:879

### O10 Methodologies for evaluation of clinical tests in their early stages of development

#### S. Graziadio^1^, A. J. Allen^2^, K. Wilson^3^

##### ^1^NIHR Newcastle In Vitro Diagnostics Co-operative, Newcastle upon Tyne Hospitals Foundation Trust, Newcastle upon Tyne, UK; ^2^NIHR Newcastle In Vitro Diagnostics Co-operative, Newcastle University, Newcastle upon Tyne, UK; ^3^Mathematics, Statistics and Physics, Newcastle University

***Background***

In early diagnostic test evaluations the potential benefits of the introduction of a new technology in the current healthcare system are assessed in the challenging situation of limited empirical data. These evaluations provide tools to evaluate which technologies should progress to the next stage of evaluation.

***Objectives***

We aim to identify new approaches within the Bayesian framework for care pathway analysis for early test evaluations.

***Methods***

In this study a diagnostic test for patients suffering from Chronic Obstructive Pulmonary Disease (COPD) was evaluated with Bayesian networks, which provide a compact visualization of probabilistic dependencies and interdependencies. The structure of the network was inferred from the care pathway, a schematic representation of the journey of a patient in the healthcare system. After the network was inferred and reduced with arc reversal techniques, it was populated using expert judgement elicitation. The Bayesian network was then queried to evaluate whether the introduction of the test could reduce unnecessary hospital admissions. Uncertainty analyses were used to determine credible intervals for the comparison between the current and new pathway, and to identify influential parameters of the decision problem.

***Results***

We found that the adoption of the diagnostic test had the potential to reduce the number of missed COPD exacerbations of symptoms that could lead to late hospital admissions, and of unnecessary visits to A&E. The model inputs that most influenced the posterior distribution were identified as the probability that a patient would go to A&E if an exacerbation was suspected, the probability that the healthcare professionals in primary care refer patients to the hospital, and the sensitivity of the test.

***Conclusion***

These results are useful to companies to inform the choice of the target population, of potential early adopters and the identification of the technological focus to guide development of the test.

### O11 Practical recommendations for diagnostic accuracy studies in low prevalence situations

#### G. A. Holtman^1,2^, M. Y. Berger^2^, H. Burger^2^, J. J. Deeks^3^, N. Donner-Banzhoff^4^, T. R. Fanshawe^1^, C. Koshiaris^1^, M. M. Leeflang^5^, J. Oke^1^, R. Perera^1^, J. B. Reitsma^6^, A. van den Bruel^1^

##### ^1^Nuffield Department of Primary Care Health Sciences, Radcliffe Observatory Quarter, University of Oxford, Oxford OX2 6GG, UK; ^2^Department of General Practice and Elderly Care Medicine, University Medical Centre Groningen, University of Groningen, PO Box 196, 9700 AD Groningen, the Netherlands; ^3^Institute of Applied Health Research, Public Health Building, University of Birmingham, Birmingham B15 2TT, UK; ^4^Department of General Practice and Family Medicine, Faculty of Medicine, Philipps University of Marburg, Karl-von-Str. 4, Marburg 35037, Germany; ^5^Department of Clinical Epidemiology, Biostatistics and Bioinformatics, Academic Medical Center, University of Amsterdam, PO Box 22700, 1100 DE Amsterdam, the Netherlands; ^6^Julius Center for Health Sciences and Primary Care, University Medical Center Utrecht, PO Box 85500, 3508 GA Utrecht, the Netherlands

###### **Correspondence:** G. A. Holtman

**Background**

The methodological challenge in low prevalence situations is that a classical diagnostic accuracy design requires large sample sizes to estimate sensitivity with adequate precision. Reducing sample sizes without introducing risk of bias is challenging.

**Objectives**

To collate and discuss designs and methods of diagnostic accuracy studies which can be used in low prevalence situations.

**Methods**

We performed a literature search in four electronic databases (Cochrane Library, Embase, Medline, Web of Science), used backward citation tracking, and invited experts to identify studies with relevant designs or methods. Two reviewers independently included studies describing a study design or method for estimating diagnostic accuracy in a low prevalence situation. Studies on prognostic tests or impact studies of diagnostic tests were excluded. During a one-day meeting with the expert group, the list of methods was discussed and recommendations were formulated.

**Results**

We identified four designs for single binary tests, one design for multiple conditions, and one design for comparing two tests without verification of double negatives. The four designs for single binary tests were stratification design, two-phase design, case-control design, and nested case-control design. Figure 1 shows the classical diagnostic accuracy design and the six designs that could reduce the total number of patients or the number of patients undergoing the reference standard or index test. Table 1 provides a ranking of the most suitable designs including potential pitfalls. Additionally, we formulate recommendations for sample size calculation, logistic regression analysis in small datasets, use of big datasets and population weighting.

**Conclusion**

This overview of designs and methods could help researchers design a diagnostic accuracy study in low prevalence situations. Researchers may consider this in future studies and understand the advantages and limitations.

**Fig. 1 (abstract O11).**
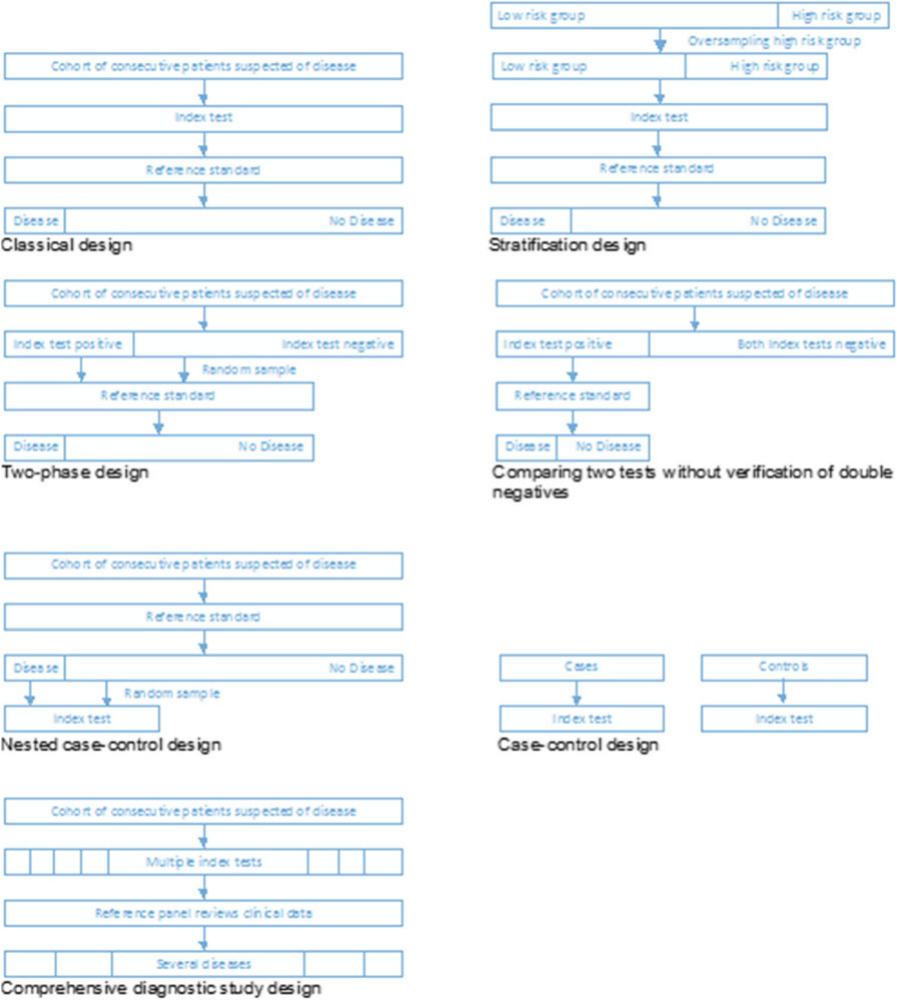
Study designs for diagnostic accuracy studies

**Table 1 (abstract O11).** Summary of design ranking in different methodological situations

	Stratification design	Two-phase design	Case-control design	Nested case-control design	Comprehensive diagnostic study design	Comparing two tests without verification of double negatives
Methodological situation:						
*Population*						
Different strata in patient population	Only option					
*Index test*						
Stored data available on index test				Only option		
Costly or invasive index test	Second option		Third option	First option		
Two index tests	Third option	Second option				First option
Multiple index tests					Only option	
*Reference standard*						
Costly or invasive reference standard	Third option	First option				Second option
*Target condition*						
Extremely low prevalence (<0.001%)			Only option			
Multiple target conditions					Only option	
Risk of Bias QUADAS-2	Higher risk of bias if there is more difference between prevalence strata	Higher risk of bias if the selection of reference standard is not random	Higher risk of bias if cases or controls do not represent the same population	Higher risk of bias if the selection of index test is not random	Higher risk of bias if there is no reference panel to determine the diagnosis	Higher risk of bias if both index tests are not blinded from each other

If there were more than one design applicable, the first three options were ranked on suitability based on sample size and risk of bias. Other designs might be possible as well in specific situations.

### O12 Quantifying overdiagnosis. Lessons learnt from a health technology assessment of low dose CT screening for lung cancer

#### H. Yang^1^, T. Snowsill^2^, Peters J.^3^, C. Hyde^3^

##### ^1^PenTAG, University of Exeter, UK; ^2^Health Economics Group, University of Exeter, UK; ^3^ Exeter Test Group, University of Exeter, UK

###### **Correspondence:** C. Hyde

**Background**: Diagnosis of lung cancer frequently occurs in its later stages. Low-dose computed tomography (LDCT) could detect lung cancer early but like all screening there is risk of overdiagnosis.

**Objective**: To estimate the effectiveness of LDCT in high risk populations to inform the UK National Screening Committee.

**Methods**: A systematic review of randomised controlled trials (RCTs) was conducted, comparing LDCT screening programmes with usual care (no screening) or other imaging screening programmes (such as chest X-ray). Meta-analyses were performed. We focus on the findings relating to overdiagnosis in this presentation.

**Results**: Twelve RCTs were included in the review, only three of which currently contribute evidence on lung cancer numbers. Compared with controls (usual care/best available care), LDCT screening was associated with a statistically significant increase (pooled RR 1.38, 95% CI 1.02 to 1.86) with at least five years follow-up. Our findings further demonstrated a shift due to LDCT screening on the stage distribution towards earlier stages for detection of lung cancers. LDCT screening was associated with a statistically significant increase in early stage (I and II) cancer detection (pooled RR 1.73, 95% CI 1.27 to 2.37) with a corresponding statistically significant decrease in late stage (III and IV) cancer. There was a statistically significant reduction in the absolute risk of late stage lung cancer, indicating that there is an element of actual stage shift (pooled RR 0.85, 95% CI 0.73 to 1.00).

**Conclusion**: There was clear evidence of overdiagnosis, but the degree to which we could quantify this was constrained. We will reflect on whether we could improve on this in future up-dates of the systematic review and what data would be required in order to do this. We will also consider how claims from screening advocates that overdiagnosis can be easily mitigated by improved radiological techniques can be tested.

### O13 Use of test accuracy study design labels in NICE’s Diagnostic Guidance

#### M. Olsen^2^, J. L. Peters^1^, Z. Zhelev^1^, H. Hunt^1^, B. Grigore^1^, P. Bossuyt^2^, C. Hyde^1^

##### ^1^Exeter Test Group, University of Exeter, UK; ^2^AMC, University of Amsterdam, Netherlands

###### **Correspondence:** C. Hyde

**Background**: Although there are a variety of approaches to evaluating the accuracy of tests, the terms used to describe these approaches are limited and lack standardization. In parallel with ongoing research to develop a more rational and informative set of study design labels for test accuracy, we are investigating the use made of study design labels in the diagnostic guidance of one national policy making body, NICE.

**Objectives**: To describe the range of study design terms used and to investigate whether different weight is given to different study designs in the final guidance.

**Methods**: We will extend the approach used in past analysis of the methodological features of NICE guidance. All NICE Diagnostics Guidance and underpinning summaries of the evidence will be interrogated, focusing on tests used for diagnosis. We will abstract data on: the policy question addressed; the accuracy evidence found and the inclusion criteria for the reviews of it; the study design terms used to describe the evidence; the quality assessment process; whether the evidence was sub-divided by different study designs; and whether the final guidance recognized any differences in study design. Analysis will be qualitative.

**Results**: Earlier investigations suggest little use of study design terms to recognize differences in accuracy study design. We will extend these initial observations.

**Conclusion**: The lack of a series of study design terms which quickly and reliably convey study designs which have different levels of intrinsic bias is an important barrier to good reporting of accuracy studies. However it is also critical for good secondary research. Without such terms all accuracy studies may be considered equal with quality assessment tools being the only means to recognize varying threat to validity arising from different study designs. These tools have not usually been designed for this purpose.

### O14 Dichotomization of the reference standard: Do we force expert panels into wrong decisions?

#### K. Jenniskens^1^, C. A. Naaktgeboren^1^, J. B. Reitsma^1,3^, K. G. M. Moons^1,3^, M. van Smeden^2^

##### ^1^Julius Center for Health Sciences and Primary Care, University Medical Center Utrecht, Utrecht, The Netherlands; ^2^Department of Clinical Epidemiology, Leiden University Medical Center (LUMC), Leiden, the Netherlands; ^3^Dutch Cochrane Centre, University Medical Center Utrecht, Utrecht, The Netherlands

###### **Correspondence:** K. Jenniskens

***Background***

When there is no one single perfect reference standard to which an index test can be compared in a diagnostic test accuracy (DTA) study, an alternative option can be to have a panel of clinicians evaluate the presence of disease based on a combination of tests. Expert panels are typically forced to make black and white decisions, ignoring any uncertainty that may still be present in individual patients. This dichotomization of disease can lead to biased DTA estimates of the test of interest (index test).

***Objective***

To demonstrate how forcing dichotomization of disease can lead to biased diagnostic accuracy estimates of an index test.

***Methods***

In this simulation study the following parameters were varied: the number and accuracy of tests used in the panel, the accuracy of the index test, and disease prevalence. For each possible combination of test results the probability of obtaining that test pattern and corresponding likelihood of disease were calculated. Individuals with test patterns with a disease probability of >0.5 were classified diseased, or otherwise as non-diseased. This dichotomization was used to calculate bias in index test DTA estimates.

***Results***

The amount of bias in index test DTA estimates depends on the magnitude and combination of number and accuracy of component tests, disease prevalence, as well as the true value of the index test. The Fig. 1 shows the bias of index test sensitivity and specificity in our base-case scenario (four component tests, with sensitivity and specificity of 70%, and a prevalence of 0.2). Although prevalence does not affect reference standard performance, it can have significant and unpredictable impact on bias in index test DTA estimates.

***Conclusion***

Forcing dichotomization of probabilistic estimates of a reference standard can lead to significantly biased estimates of index test sensitivity and specificity. Researchers should consider implementing probability estimates when working with expert panels.

**Fig. 1 (abstract O14).**
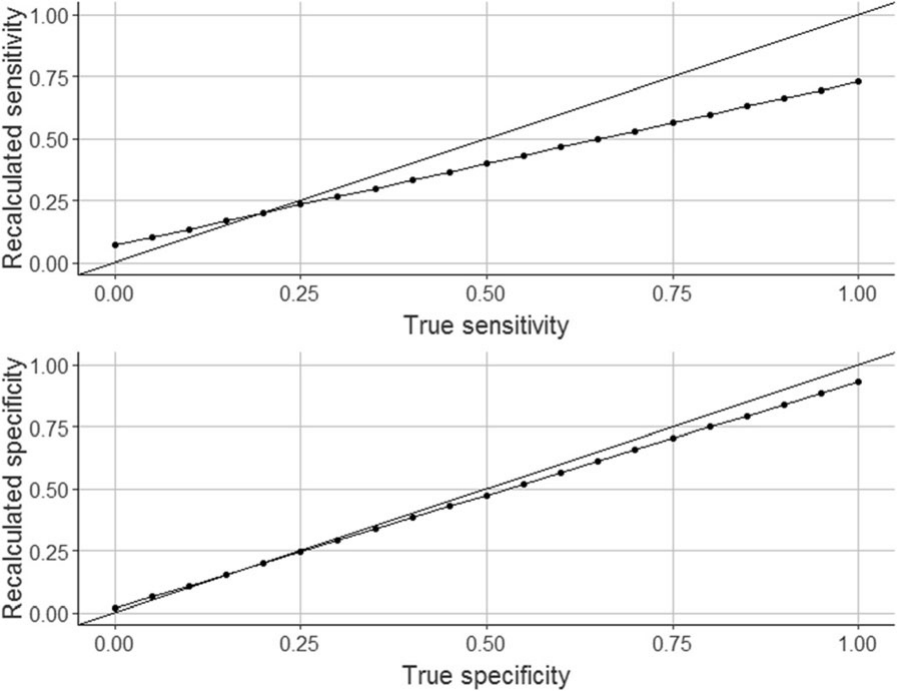
Bias in estimates of index test sensitivity and specificity for a range of true values as result of dichotomization of the reference standard

### O15 Quantifying how diagnostic test accuracy depends on threshold in a meta-analysis

#### H. E. Jones^1^, C. A. Gatsonis^2,3^, T. A. Trikalinos^3^, N. J. Welton^1^, A. E. Ades^1^

##### ^1^Population Health Sciences, Bristol Medical School, Bristol, UK; ^2^Center for Statistical Sciences, School of Public Health, Brown University, USA; ^3^Center for Evidence Synthesis in Health, School of Public Health, Brown University, USA

###### **Correspondence:** H. E. Jones

**Background**:

Since the most appropriate threshold at which to operate a test is usually a key clinical question, there is a need to move beyond standard meta-analysis methods which: (i) do not provide summary estimates of accuracy at each threshold and (ii) can only synthesise a single pair of sensitivity and specificity from each study, despite studies often reporting data at more than one threshold. Some more advanced methods have recently been proposed, notably that of Steinhauser *et al* (2016), but a limitation is the need to pre-specify the distributional form of test results in the diseased and disease-free populations.

**Objectives**:

To develop a meta-analysis model which (i) provides estimates of the sensitivity and specificity of a test across all thresholds, (ii) makes use of all available data, (iii) makes less restrictive assumptions about the distributional form of test results than recently proposed approaches, (iv) works directly with count data (numbers of patients with test results above each threshold), rather than requiring normal approximations.

**Methods**:

We describe a multivariate meta-analysis model for count data, that can take any number of counts from each study and explicitly quantifies how accuracy depends on threshold. The model allows for a flexible range of distributions of underlying test results by estimating a transformation parameter as part of the model. We fit the model in Bayesian statistical software such as WinBUGS or JAGS.

**Results**:

We demonstrate with a case study meta-analysis, quantifying the accuracy of B type natriuretic peptide in diagnosing acute heart failure.

**Conclusion**:

Our new meta-analysis model estimates the sensitivity and specificity of a continuous test at all thresholds, and does not require the analyst to pre-specify the distributional form of underlying test results. Further, the model does not require normal approximations, which can perform poorly in the presence of small counts.

### O16 Developing and updating prediction models in large clustered data sets

#### V. M. T. de Jong^1^, K. G. M. Moons^1^, M. J. C. Eijkemans^1^, T. P. A. Debray^12^

##### ^1^Julius Center, UMC Utrecht, Utrecht University, Utrecht, the Netherlands; ^2^Cochrane Netherlands, Julius Center, UMC Utrecht, Utrecht University, Utrecht, the Netherlands

###### **Correspondence:** V. M. T. de Jong

**Background**

Prediction models are often developed on a single data set, therefore performance in different settings and populations is frequently poor. If so, one may validate and update or tailor the model to the validation situation at hand, but this is not always feasible if performance is too poor and the validation data set is too small. We propose to use measures of generalizability in the development process of prediction models already, in the case of using large clustered development data sets.

**Objectives**

The aim of our methodology is to produce developed models that are more robust when applied across different settings and populations, and to prevent the need for constant validation and tailoring to local settings.

**Methods**

We apply several measures, namely existing measures such as the coefficient of variation, GINI’s mean difference and the pooled variance as well as newly developed measures, in a variable selection procedure for developing a prediction model until it attains optimal performance within and across different settings and populations.

**Results**

We illustrate our proposed approach by modelling 30-day mortality of patients in critical care units. Using independent validation samples for the developed models, we assess the Brier score, calibration slope and c-statistic of the models. We perform a meta-analysis of these performance statistics to assess generalizability of the prediction model (e.g. as quantified by the between-cluster heterogeneity).

**Conclusion**

Our new approaches can be used for prediction model development in large clustered data sets, to develop better generalizable prediction models.

### O17 Understanding the adoption and use of new tests using Multi-Criteria Decision Analysis: a case study on point-of-care tests in Dutch general practices

#### M. M. A. Kip^1^, J. M. Hummel^1^, E. B. Eppink^1^, H. Koffijberg^1^, R. M. Hopstaken^2^, M. J. I. Jzerman^1^, R. Kusters^1,3^

##### ^1^University of Twente, MIRA institute for Biomedical Technology and Technical Medicine, Department of Health Technology and Services Research, Enschede, The Netherlands; ^2^Saltro Diagnostic Centre, Utrecht, The Netherlands; ^3^Jeroen Bosch Ziekenhuis, Laboratory for Clinical Chemistry and Haematology, Den Bosch, the Netherlands

###### **Correspondence:** M. M. A. Kip

**Background**

After impact evaluation has demonstrated the added benefits of a new test, actual implementation is crucial to achieve these benefits in practice. Although point-of-care (POC) tests commonly offer benefits in terms of low turn-around-time, and improved patient’s satisfaction and health outcomes, only few are implemented in practice.

**Objectives**

This study aims to identify which criteria are, in general, important in the decision to implement and use a POC test, and to determine their weight. Two POC tests available for use in Dutch general practices (i.e. CRP and HbA_1c_) serve as case studies.

**Methods**

Relevant criteria were identified based on a literature review and semi-structured interviews with twelve experts in the field. Subsequently, the criteria were clustered in four groups (i.e. user, organization, clinical value, and socio-political context). The relative importance of each criterion was determined using Multi-Criteria Decision Analysis (MCDA), with the Analytic Hierarchy Process. During this group session, priorities of ten experts regarding both POC tests were elicited, as compared with central laboratory testing.

**Results**

Of 20 criteria in four clusters, the test’s clinical utility, its technical performance, and risks associated with the test-based treatment decision were considered most important for using a POC test, with relative weights of 22.2%, 12.6% and 8.5%, respectively. Overall, the experts preferred POC CRP over its laboratory equivalent, whereas they did not prefer POC HbA_1c_. This difference was mainly explained by their strong preference for POC CRP regarding the subcriterion clinical utility.

**Conclusion**

MCDA can be a valuable tool to identify criteria, and their relative impact, affecting test implementation in practice. Insight into the criteria and their weights identified in the case studies may facilitate implementation of existing POC tests. Having experts score new POC tests on these criteria provides developers with recommendations on how to increase the probability of successful implementation.

### O18 From test impact assessment to optimizing test impact: maximizing colorectal screening benefits using a meta-model including capacity-constraints

#### H. Koffijberg^1^, V. M. H. Coupé^2^, M. J. I. Jzerman^1^, K. Degeling^1^, M. J. E. Greuter^2^

##### ^1^Department of Health Technology & Services Research, MIRA Institute for biomedical technology and technical medicine, University of Twente, Enschede, the Netherlands; ^2^Department of Epidemiology and Biostatistics, VU University Medical Center, Amsterdam, the Netherlands

###### **Correspondence:** H. Koffijberg

**Background**

Model-based impact analyses are typically useful for assessing the cost-effectiveness of a limited, pre-defined, number of alternative testing strategies, none of which may be optimal.

**Objectives**

To illustrate how using a meta-model allows optimization of test use/impact, in a case study on screening for colorectal cancer, while accounting for colonoscopy capacity constraints.

**Methods**

Screening strategies were defined by starting age, screening interval, number of screening rounds, and FIT test positivity threshold (>50,000 unique strategies). A limited sample of predefined strategies (n=150) was evaluated with the validated ASCCA simulation model to identify the best screening strategy therein, in terms of life-years gained (LYG), compared with no screening. A Gaussian Process meta-model was fitted to this sample and discrete evolutionary programming was applied to determine the optimal screening strategy (GP-DEP approach) for different colonoscopy capacity constraints. Sample size of predefined strategies was varied (n=25-200) to assess GP-DEP performance using bootstrapping, brute force exhaustive search, and comparison with ASCCA outcomes.

**Results**

GP-DEP provided stable optimal screening strategies for sample sizes n>=100. Compared with ASCCA, LYG and costs of the optimal strategies from GP-DEP were accurate and slightly too high, respectively. However, performance ranking of strategies was similar according to ASCCA and GP-DEP. GP‑DEP resulted in better screening strategies (higher number of LYG) compared to just evaluating predefined strategies, for different capacity constraints (see Fig. 1). For sample size n=100 average predicted benefit of the optimal strategy identified by GP-DEP compared to the best strategy identified by ASCCA equalled 0.028 LYG (95%CI 0.013-0.043) per individual.

**Conclusion**

It is feasible and beneficial to optimize rather than evaluate test impact. Optimization using a meta-model of the ASCCA model allowed fast identification of the optimal screening strategy, even when constraints apply, and outperformed the best screening strategy as typically identified from a limited sample of predefined strategies.

**Fig. 1 (abstract O18).**
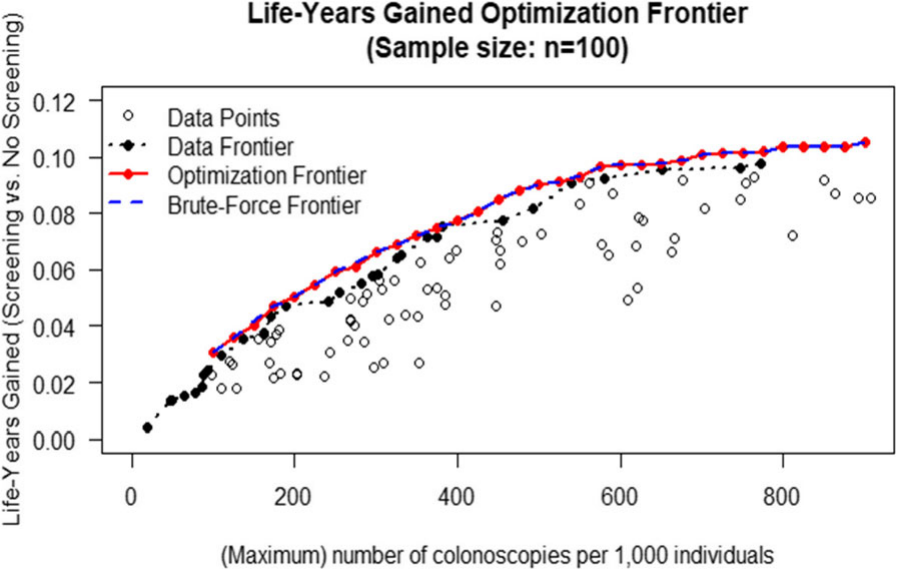
See text for description

### O19 Correction for confounding in comparative accuracy reviews

#### M. M. G. Leeflang

##### Academic Medical Center, University of Amsterdam; Clinical Epidemiology, Biostatistics and Boinformatics; Amsterdam, the Netherlands

**Background:** The credibility of comparative diagnostic accuracy reviews threatened by the lack of studies directly comparing the accuracy of one test versus that of another test. If a single test performs well in one study, and a second test less so in a different study, it is unclear whether this difference in performance is genuine, or due to differences in study design. Comparative accuracy reviews based on single test studies therefore lead to invalid conclusions and there is not yet a solution to this problem.

**Objectives:** To investigate whether a causality research approach provide opportunities to adjust for confounding in comparative accuracy research.

**Methods:** When answering the question which test has the highest accuracy, then a causal inference framework may become relevant. If test A was investigated in a different patient population than test B, then differences between the tests may be caused by population rather than test characteristics. As confounding may play a role in the comparison between tests, the concepts from causality research may be used to remove bias in these meta-analyses. A pilot study was done with a seven-step approach for choosing covariates from a directed acyclic graph to adjust for.

**Results:** We re-analyzed a systematic review of over 150 studies on rapid tests for influenza, 10 studies evaluated the Directigen test and 21 evaluated the Quickvue test. Before adjustment, the sensitivity of the Directigen test was 77% and that of the Quickvie test 49%; after adjustment, the sensitivity was 79% for Directigen and 69% for Quickvue. However, this is only one small pilot and there is no gold standard for unconfounded comparisons in this review.

**Conclusion:** This presentation explains why and how causal inference may have a place in (comparative) diagnostic test accuracy research and will discuss the pitfalls when translating these concepts to diagnostic accuracy.

### O20 Estimating diagnostic test accuracy in the context of incomplete reporting across cutoff thresholds: A comparison of conventional meta-analysis of published data, two modelling approaches using published data, and individual participant data meta-analysis

#### B. Levis^1,2^, G. Rücker^3^, H. E. Jones^4^, B. D. Thombs^1,2^, A. Benedetti^2^, and the DEPRESSD Research Group

##### ^1^Lady Davis Institute for Medical Research, Jewish General Hospital, Montréal, Québec, Canada; ^2^McGill University, Montréal, Québec, Canada; ^3^Medical Faculty and Medical Center - University of Freiburg, Freiburg, Germany; ^4^University of Bristol, Bristol, United Kingdom

###### **Correspondence:** B. Levis

Background: In studies of diagnostic test accuracy of ordinal tests, results are sometimes only reported for cutoff thresholds that generate desired results in a given study (e.g., high combined sensitivity and specificity). When combining results in meta-analyses, selective cutoff reporting may result in biased accuracy estimates. One way to overcome this bias is via individual participant data meta-analysis (IPDMA). Another approach is to use published results, but to model missing cutoff data using statistical techniques.

Objectives: To compare IPDMA of data from all studies and cutoffs to three approaches for estimating diagnostic test accuracy using published data in the context of missing cutoff data: (1) conventional meta-analysis using bivariate random-effects meta-analysis, and modeling missing cutoff data using multiple cutoff models developed by (2) Steinhauser et al. and (3) Jones et al.

Methods: We analyzed data collected for an IPDMA of Patient Health Questionnaire-9 depression screening tool accuracy. We compared sensitivity and specificity estimates from conventional meta-analysis of published results, the two modelling approaches, and IPDMA. The modeling approaches were applied to the published dataset blind to IPDMA results.

Results: 15,020 participants (1,972 cases) from 45 studies were analyzed. All methods produced similar specificity estimates. Compared to IPDMA, conventional bivariate meta-analysis underestimated sensitivity for cutoffs <10 and overestimated sensitivity for cutoffs >10 (mean absolute difference: 6%). For both modeling approaches, sensitivity was slightly underestimated for all cutoffs (mean underestimation: 2%).

Conclusion: IPDMAs are the gold standard for evidence synthesis, but are labor intensive. In the context of missing cutoff data, applying modeling approaches to published data is more efficient than IPDMA and produces accuracy estimates that more closely resemble IPDMA than not modeling. However, applying modeling approaches to published data resulted in a slight underestimation of sensitivity in our case study and precludes the possibility of assessing accuracy in participant subgroups.

### O21 Impact of non-transportable diverse measurement of predictors on performance of prediction models: a measurement error perspective

#### K. Luijken, R. H. H. Groenwold, M. van Smeden

##### Department of Clinical Epidemiology, Leiden University Medical Center - LUMC, the Netherlands

###### **Correspondence:** K. Luijken

**Background:** Transportability of prediction models can be hampered when predictors are measured differently at development and (external) validation. This may occur, for instance, when predictors are measured using different cut-off points or when tests are produced by different manufacturers. While such heterogeneity in predictor measurement across development and validation seems very common, little is known about the impact it may have on the performance of prediction models at external validation.

**Objectives:** To define effects of predictor measurement heterogeneity on external performance of prediction models, by taking a measurement error perspective to describe measurement heterogeneity.

**Methods:** Using analytical and simulation approaches, we examined the external predictive performance of a clinical prediction model under different scenarios of heterogeneous predictor measurement, using a well-known taxonomy of measurement error models to recreate heterogeneity in measurement procedures.

**Results:** Heterogeneity in measurements of predictors can have a large impact on the external predictive performance of a prediction model, often leading to worse but possibly to improved external predictive performance. This may result in either overfitted or underfitted prediction models, to extents that the prediction model may no longer be clinically useful. Furthermore, our simulation study showed that commonly recommended shrinkage strategies (e.g. Ridge regression) may both improve or worsen the impact of heterogeneity in measurement procedures on the external predictive performance.

**Conclusion:** Our work highlights measurement heterogeneity as an important explanation of unanticipated out-of-sample performance of clinical prediction models, as dissimilarities in the measurements of tests and markers between development and validation deteriorate the actual predictive power of the model at external validation.

### O22 Biomarkers to detect active tuberculosis: a systematic review of the evidence, quality, and progress from 2010-2016

#### Emily MacLean^1^, Tobias Broger^2^, Seda Yerlikaya^2^, Madhukar Pai^1^, Claudia Denkinger^2^

##### ^1^Department of Epidemiology, Biostatistics, and Occupational Health, McGill International TB Centre; ^2^FIND, Geneva, Switzerland

###### **Correspondence:** Emily MacLean

**Background:** As 1.8 million of the 10.4 million people with new TB cases die each year, timely diagnosis and treatment initiation is critical. However, traditional diagnostic methods, such as culture or smear microscopy, are slow or low in sensitivity; more modern techniques, such as sequencing or GeneXpert MTB/RIF, are inaccessible to populations at greatest risk of contracting TB. The WHO has proposed biomarkers as the bases for needed new diagnostic assays to detect active TB.

**Objectives:** To assemble the existing biomarkers and biosignatures used to identify active TB and evaluate the quality and level of evidence around them.

**Methods:** In collaboration with FIND and in relation to WHO’s high-priority Target Product Profiles (TPPs), we conducted a systematic review of biomarkers for the detection of active TB. A comprehensive search term was composed and used in multiple scientific databases.

**Results:** Initially, 6543 publications from 2010 to 2016 were identified. After deduplication, 3970 records were screened by title and abstract. Finally, 374 publications fulfilled the inclusion criteria. Types of biomarkers identified included antibodies, cytokines, metabolic activity markers, Mycobacterial antigenic proteins, RNA, and volatile organic compounds. Only 51% of studies reported a culture-based reference standard and diagnostic performance data beyond p-values. Risk of bias due to study design was generally high.

Only 8% of studies were considered high-quality and met TPP minimum criteria for sensitivity and specificity. Frequently, publications repeated the findings of other discovery-phase studies without moving the biomarker to further developmental stages.

**Conclusions:** Validation studies that incorporate intended diagnostic use-cases are needed. The extracted data are currently being used by FIND as the foundation of a dynamic database where biomarker data and developmental status will be presented. Ultimately, this database will enable developers and researchers to populate the TB biomarker pipeline, accelerating diagnostic test development.

**Fig. 1 (abstract O22).**
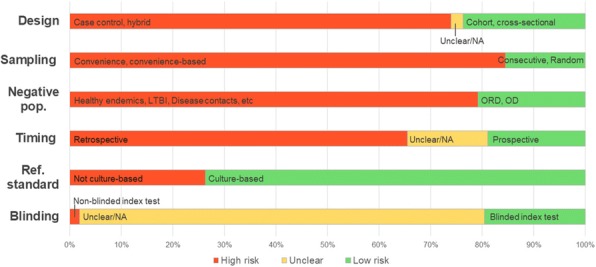
Risk of bias; percentages are of total included studies

### O23 Challenges in the management of trials of medical tests

#### S. Mallett^1,2^, C. Rick^3^, J. Brown^3^, W. McKinnon^3^, R. Ottridge^3^, A. Palmer^3^, V. Parker^3^, L. Priest^3^ and J. Deeks^1,2,3^

##### ^1^Test Evaluation Research Group, Institute of Applied Health Sciences, University of Birmingham, Edgbaston, Birmingham B15 2TT, United Kingdom; ^2^NIHR Birmingham Inflammation Biomedical Research Centre, University of Birmingham, Edgbaston, Birmingham B15 2TT, United Kingdom; ^3^Birmingham Clinical Trials Unit, Institute of Applied Health Sciences, University of Birmingham, Edgbaston, Birmingham B15 2TT, United Kingdom

###### **Correspondence:** S. Mallett

**Background:** Trials of medical tests present a series of challenges in their set-up and management that differ from randomised controlled trials (RCTs) of interventions. Birmingham Clinical Trials Unit (BCTU) manages and provides statistical support for a wide range of test evaluation trials as well as RCTs of interventions.

**Objective:** To identify unique challenges in the set-up and management of trials of tests in order to improve future trial design and management.

**Method:** Within the CTU we set up a working group to review experience of ten trials of tests for diagnosis, staging, screening and monitoring. We identified themes where particular challenges were noted which did not occur or were different for RCTs of interventions.

**Results:** The ten studies covered bladder overactivity, chronic kidney disease, thyroid nodules, neoplasia in chronic colitis, maternal group B streptococcal colonisation, causes of pelvic pain, ovarian cancer, extent and activity of Crohn’s disease, staging of lung & colorectal cancer, and staging and management in ovarian cancer. Tests included: PET-CT, CT, MRI and ultrasound, biomarker measurements, development and evaluation of biomarker panels and near patient and laboratory based IVDs.

Ten topics were identified that appear unique or to have higher impact on test studies than intervention RCTs including specific issues in: ethics and governance, patient selection, recruitment, uncertainty of diagnostic results, test processes and pathways, sample preparation and measurements, reference standards, follow up, adverse effects and diagnostic impact.

**Discussion:** While some of these themes also occur in RCTs, the relative importance or risks differ from those in test studies. These themes will be presented in more depth using examples from the ten trials and strategies used to resolve or minimise the impact in specific trials will be reviewed. Identifying challenges in these studies is important to enhance the design and conduct of future test studies.

### O24 Development of Medical Device Key Evidence Tool (‘MEDKET’): an evidence-based framework to explain Medical Device (MD) success in selected European and US companies

#### S. Manetti^1^, M. Ni^2^, G. Turchetti^1^

##### ^1^Institute of Management, Scuola Universitaria Superiore Sant’Anna, Pisa, Italy; ^2^NIHR-London IVD Co-operative, Department of Surgery and Cancer, Imperial College, London, UK

###### **Correspondence:** S. Manetti

**Background**

The incorporation of early Health Technology Assessment (HTA) might be beneficial for Medical Device (MD) industry; however, evidence that industry is conducting early HTA remains scarce.

**Objectives**

This study aims to develop an evidence-based framework to understand whether, and to which extent, early HTA might drive product success of small and large enterprises (SEs and LEs).

**Methods**

This research encompassed four stages (Fig. 1). We conducted a key-informant process (stage 1) where 25 international experts identified a list of emergent HTA themes that they believed were important to company success. A sample of 22 European and US selected companies then reached consensus on a list of key themes through a robust Delphi process (stage 2). Finally, in stage 3, we constructed the ‘MEDKET’ checklist for SEs and LEs by defining and prioritizing key themes using comments and ratings from stage 1 and 2.

**Results**

We found out that SEs perceived success as business continuity, whereas LEs identified success as large-scale utilization and patient/user value. ‘MEDKET’ for SEs and for LEs included, respectively, 21 and 15 items, with 9 overlapping themes. In both groups, success was driven by three item categories: (i) R&D processes (e.g. starting time of assessment activities); (ii) device outcome-measures (e.g. economic sustainability); (iii) structural peculiarities of company (e.g. business model) and of MD market (e.g. being an incumbent in the reference market segment).

**Conclusion**

Our results showed that early HTA plays a pivotal role in MD industry success and it could have different implications based on enterprises size. On one hand, the adoption of early HTA in SEs setting could be vital for the companies’ survival. On the other hand, investing the proper amount of funds in early HTA by LEs can increase the overall R&D efficiency; however, the allocation of additional funds after a certain threshold does not guarantee MD success.

**Fig. 1 (abstract O24).**
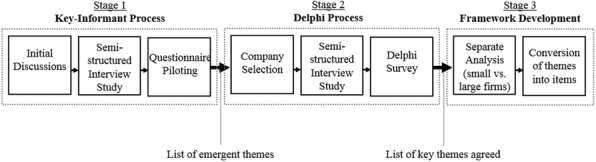
Study outline

### O25 Implementation and effects of risk-dependent obstetric care in the Netherlands: a clinical impact study (Expect Study II)

#### Pim van Montfort^1^, Hubertina C. J. Scheepers^2^, Luc J. M. Smits^1^, On behalf of the Expect Study group

##### ^1^Department of Epidemiology, Care and Public Health Research Institute (CAPHRI), Maastricht University, The Netherlands; ^2^Department of Obstetrics and Gynecology, School for Oncology and Developmental Biology (GROW), Maastricht University Medical Centre, The Netherlands

###### **Correspondence:** Pim van Montfort

**Background:** This study compares former obstetric care as usual (Expect I) with risk-dependent care using a prediction tool (Expect II). The Expect I study externally validated 39 prediction models using data of 2,614 women prospectively included from 2013 to 2015. Clinically useful models were embedded in a web-based prediction tool. Additionally, risk-dependent care paths were developed, resulting in antenatal care tailored to the outcomes of individual risk assessments. Risk-dependent care was embraced by a consortium of obstetric healthcare professionals in the Dutch province of Limburg.

**Methods:** Women receiving risk-dependent care are being enrolled in a prospective multicenter cohort (Expect II). Primary outcomes are adherence of healthcare professionals and compliance of women to key recommendations; e.g. adequate calcium intake in all women (Expect I, adequate calcium intake in 34% of women) and low-dose aspirin treatment to women at increased risk of preeclampsia (Expect I, actual use in the high-risk group: 1.5%).

**Preliminary results:** Ten months after introduction our prediction tool is being used in an estimated 24-40% of pregnant women (Fig. 1). Currently, 435 women have been enrolled. Recommendations regarding calcium intake were discussed with 351 women (81%), of which 285 (81%) reported the intention to comply. In case of an elevated preeclampsia risk (n=223) preventive aspirin treatment was discussed with 180 women (76%), of which 52 (29%) intended to comply.

**Conclusion:** The preliminary results indicate risk-dependent care has been implemented by a reasonable proportion of healthcare professionals. Furthermore, usage of the prediction tool appears to increase recommendation of preventive interventions. Implementing new guidelines asks an additional effort of caregivers, especially if implementation requires a reorganization of their routine and includes novel strategies such as a prediction tool. Future research should focus on barriers that hamper the adherence of healthcare professionals to risk-dependent care and on reasons for non-compliance of women.

**Keywords:**

Risk-dependent care, prediction, adherence, compliance

**Fig. 1 (abstract O25).**
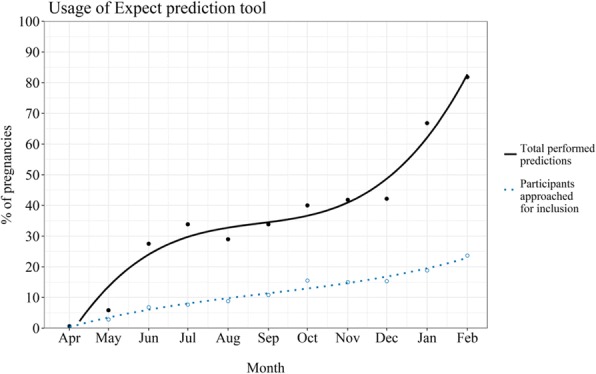
See text for description

### O26 Challenges in evaluating biomarker tests to determine eligibility for immunotherapy that has pan-tumour activity: one sized evaluation does not fit all

#### Judy K. Morona, Tracy Merlin

##### Adelaide Health Technology Assessment (AHTA), School of Public Health, The University of Adelaide, Australia

###### **Correspondence:** Judy K. Morona

**Background:**

The United States Food and Drug Administration (FDA) granted accelerated approval for the check-point inhibitor, pembrolizumab, to treat patients with locally-advanced or metastatic solid tumours of any origin that are mismatch-repair deficient (dMMR) or microsatellite instability-high who have progressed after prior treatment and have no satisfactory alternative treatment options. The FDA has referred to this indication as “tissue/site agnostic”, whereas in Australia, the Medical Services Advisory Committee has referred to this as a “pan-tumour” approach.

This pan-tumour approach is new for health technology assessment groups. To date, evaluation of the (cost-)effectiveness and safety of both the targeted cancer drug and the companion diagnostic test have been assessed for specific biomarkers, such as HER2, EGFR, and BRAF, in patients with common cancer types, such as melanoma, breast, colorectal or lung cancer. For these applications, the evidence base would generally include at least one randomised trial comparing the effectiveness of the targeted treatment in either the test-positive population (including falsely-positive patients), or the whole cohort (including patients with either false-positive or false-negative results).

**Objective:**

To provide guidance on the evidence needed to evaluate pan-tumour applications.

**Method:**

We examined the effectiveness of dMMR testing for access to pembrolizumab in tumours of diverse origin.

**Results and conclusion:**

Pan-tumour populations include rare tumour types that are supported by minimal clinical evidence, such as single arm studies. There are differences in the standard of care for tumours arising from diverse sites of origin, and there are limited data for determining the accuracy of the diagnostic test in these different tumour types. Furthermore, the prevalence of dMMR was highly variable across tumour types, greatly affecting the clinical validity (PPV and/or NPV) of the test. We caution that the proportion of patients with false-positive and false-negative test results – and consequent adverse treatment outcomes - *per cancer* must be considered.

### O27 Network meta-analysis of diagnostic test accuracy studies allowing for multiple tests at multiple thresholds

#### R. K. Owen^1^, N. J. Cooper^1^, T. J. Quinn^2^, A. J. Sutton^1^

##### ^1^Department of Health Sciences, University of Leicester, Leicester, UK; ^2^Institute of Cardiovascular and Medical Sciences, University of Glasgow, Glasgow, UK

###### **Correspondence:** R. K. Owen

**Background:** Network meta-analyses have extensively been used to compare the effectiveness of multiple interventions for healthcare policy and decision-making. However, methods for evaluating the performance of multiple diagnostic tests are less established. In a decision-making context, we are often interested in comparing and ranking the performance of multiple diagnostic tests, at varying levels of test thresholds.

**Objective:** To develop a framework for evaluating multiple diagnostic tests, at varying test thresholds in one simultaneous analysis.

**Methods:** Motivated by an example of cognitive impairment diagnosis following stroke, we synthesized data from 13 studies assessing the efficiency of two diagnostic tests: Mini-Mental State Examination (MMSE) and Montreal Cognitive Assessment (MoCA), at two test thresholds: MMSE <25/30 and <27/30, and MoCA <22/30 and <26/30. Using Markov Chain Monte Carlo (MCMC) methods, we fitted a bivariate network meta-analysis model, accounting for the correlations between multiple test accuracy measures from the same study, and incorporating constraints on increasing test thresholds assuming that higher test thresholds had an increased sensitivity but decreased specificity.

**Results:** We developed and successfully fitted a model comparing multiple tests/threshold combinations while imposing threshold constraints. Applying constraints on increasing test thresholds reduced the within-study variability and increased the precision in estimates of sensitivity and specificity. Using this model, we found that MoCA at threshold <26/30 appeared to have the best true positive rate (estimated sensitivity: 0.98; 95% credible interval (CrI): 0.94,0.99), whilst MMSE at threshold <25/30 appeared to have the best true negative rate (estimated specificity: 0.84, 95%CrI: 0.79,0.88).

**Conclusions:** In a health technology assessment setting, there is an increasing need to compare the efficiency of multiple diagnostics tests. The combined analysis of multiple tests at multiple thresholds allowed for more rigorous comparisons between competing diagnostics tests for decision-making.

### O28 Variation in the measurement of predictors affects the discriminative ability and transportability of a prediction model

#### R. Pajouheshnia^1^, M. van Smeden^2^, L. M. Peelen^1^, R. H. H. Groenwold^2^

##### ^1^UMC Utrecht Julius Center, Utrecht University, Utrecht, the Netherlands; ^2^Department of Clinical Epidemiology, Leiden University Medical Centre, Leiden, the Netherlands

###### **Correspondence:** R. Pajouheshnia

**Background**: Diagnostic and prognostic prediction models often perform poorly when externally validated. The reasons for variation in performance across data samples are not fully understood.

**Objectives**: We investigate how differences in the measurement of predictors across settings affect the discriminative power and transportability of a prediction model.

**Methods**: Differences in predictor measurement between data sets can be described formally using a “measurement error” taxonomy. Using this taxonomy, we derive an expression relating variation in the measurement of a continuous predictor to the area under the curve (AUC) of a logistic regression prediction model. This expression is then used to demonstrate how variation in measurements across samples affects the out-of-sample discriminative ability of a prediction model. We illustrate these findings with a diagnostic model using example data of patients suspected of having deep vein thrombosis.

**Results**: When a predictor, such as D-dimer, is measured with more noise in one setting compared to another, which we conceptualize as a difference in “classical measurement error”, the AUC decreases (Fig. 1a). In contrast, constant, “structural”, error does not impact on the AUC of a logistic regression model, providing the magnitude of the error is the same among cases and non-cases (Fig. 1b). As the differences in measurement methods (and in turn differences in measurement error) become more complex, it becomes increasingly difficult to predict how the AUC will be affected.

**Conclusion**: When a prediction model is applied to a new sample, its discriminative ability can change if the magnitude or structure of the measurement error is not exchangeable between the two settings. This provides an important starting point for researchers to better understand how differences in measurement methods can affect the performance of a prediction model when externally validating or implementing it in practice.

**Fig. 1 (abstract O28).**
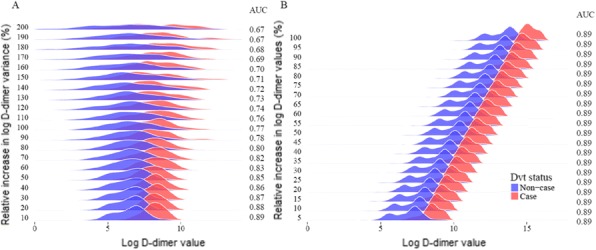
See text for description

### O29 Sample size formulae for developing a multivariable prediction model based on expected shrinkage

#### Richard D. Riley^1^, Kym I. E. Snell^1^, Joie Ensor^1^, Danielle L. Burke^1^, Karel G. M. Moons^2^, Gary S. Collins^3^

##### ^1^Centre for Prognosis Research, Research Institute for Primary Care and Health Sciences, Keele University, Staffordshire, UK. ST5 5BG; ^2^Julius Center for Health Sciences and Primary Care, University Medical Center Utrecht, Utrecht, The Netherlands; ^3^Centre for Statistics in Medicine, Nuffield Department of Orthopaedics, Rheumatology and Musculoskeletal Sciences, University of Oxford, Oxford, UK. OX3 7LD

###### **Correspondence:** Richard D. Riley (+44 (0) 1782 733905, r.riley@keele.ac.uk)

**Background**: When designing a study to develop a new risk prediction model, researchers should ensure their sample size is adequate in terms of the number of participants (*n*) and events (*E*) relative to the number of predictor parameters (*p*) considered for inclusion in the model. Current sample size calculations are based on “rules of thumb”, such as at least 10 events per predictor parameter (*EPP*), which receive much debate and criticism.

**Objectives**: To produce a new sample size formula for studies developing a prediction model with either binary or time-to-event outcomes. Specifically, to identify in advance of data collection, the sample size needed to minimize the expected optimism in predictor effect estimates, and thus the expected shrinkage required after model development.

**Methods**: We derive a closed-form sample size formula, based on utilizing the heuristic uniform shrinkage factor of Van Houwlingen and Le Cessie. The formula allows researchers to identify *n*, *p* and *EPP* that correspond to an expected shrinkage factor close to 1, such as 0.9, that reflects low overfitting. It requires researchers to pre-specify the anticipated Cox-Snell *R*^2^ of the model, and we show how to identify realistic values of *R*^2^ based on published information (e.g. C statistic) for existing models in the same field. A suitable margin of error in other relevant estimates (e.g. overall risks) is also recommended.

**Results**: We illustrate the approach using examples of diagnostic and prognostic prediction models. This shows that, to target an expected shrinkage factor of 0.9, a new diagnostic model for Chagas disease requires an *EPP* of 3.9 and a new prognostic model for recurrent venous thromboembolism requires an *EPP* of 23.

**Conclusion**: Blanket rules of thumb for sample size are inappropriate, and our alternative proposal allows sample size and *EPP* to be tailored to the particular model and setting of interest.

### O30 Meta-analysis of diagnostic test accuracy studies with multiple cutoffs: The R package diagmeta

#### G. Rücker^1^, S. Kolampally^1^, G. Schwarzer^1^, S. Steinhauser^2^

##### ^1^Institute of Medical Biometry and Statistics, Faculty of Medicine and Medical Center - University of Freiburg, Germany; ^2^Institute of Medical Statistics and Computational Biology, University of Cologne, Germany

###### **Correspondence:** G. Rücker

**Background:** We developed an R package *diagmeta* that implements our model for meta-analysis of diagnostic test accuracy (DTA) studies allowing for multiple cutoffs (Steinhauser 2016).

**Objectives:** To make this statistical method accessible to users with a background in statistics, psychology, medicine, or public health.

**Methods:** The parametric model assumes that the values of the underlying biomarker follow two correlated distributions for individuals with/without the target condition. Data can be entered either as study label, cutoff, TP (true positive), TN (true negative), FP (false positive), FN (false negative), or as individual participant data (study label, individual's measurement, status). Users can choose between several mixed linear models and specify the type of distribution (logistic or normal), and the weighting method for studies (e.g., inverse variance weighting). For determination of an optimal cutoff, weights for sensitivity and specificity can be specified.

**Results:** The output of *diagmeta* includes basic information such as the number of studies and cutoffs, the empirical distribution of cutoffs, the optimal cutoff, sensitivity and specificity at this cutoff, and the area under the summary ROC curve. For given cutoffs, pairs of sensitivity and specificity with confidence intervals can be tabulated. If a prevalence is specified, predicted values are calculated. In addition, a flexible plot function is provided to produce cumulative distribution plots, density plots, Youden index curves, study-specific ROC curves, the summary ROC curve, and the summary operating point, optionally with a corresponding confidence region.

**Conclusion:** The R package *diagmeta* implements one of few available statistical methods for meta-analysis of DTA studies with multiple cutoffs and is now readily accessible. We plan to continuously extend and update *diagmeta*, and possibly to include competing methods.

References:

Steinhauser S et al.: Modelling multiple thresholds in meta-analysis of diagnostic test accuracy studies. BMC Med Res Methodol. 2016;16(1):97.doi:10.1186/s12874-016-0196-1.

### O31 Incremental value of a new risk predictor: does the analysis method match the research question?

#### E. Schuit, C. A. Naaktgeboren, K. G. M. Moons, L. M. Peelen

##### ^1^Julius Center for Health Sciences and Primary Care, University Medical Center Utrecht, Utrecht University, Utrecht, the Netherlands

###### **Correspondence:** E. Schuit

**Background** New predictors (e.g. biomarkers) are often assessed for their incremental value on top of existing prediction models in a new dataset, but methods to assess this incremental value differ, and may actually answer different research questions.

**Objectives** To describe various approaches to assess the incremental value of a new predictor and show that they differ in the research questions they address, and the (magnitude of the) estimated incremental value they identify.

**Methods** We distinguish three approaches: assessment of incremental value with respect to 1) an existing model (“existing model”); 2) individual predictors of an existing model (“model revision”); and 3) a selection of individual predictors which may be part of an existing model (“new model development”). Using these three approaches we assessed the incremental value of the D-dimer test to a deep venous thrombosis prediction model.

**Results** The approach influences the research question that is actually addressed and influences the (magnitude of the) estimated incremental value (Table 1). The incremental value of the D-dimer test decreased with increasing adjustments of the existing model to the new dataset. In the “existing model” approach, the misfit of the existing model in the new dataset allows room for the apparent incremental value of a new predictor. The “model revision” approach solves this and has been recommended as the preferred way to assess the incremental value of a new predictor in a new dataset. In the “new model development” approach, the primary interest is not incremental value, but rather which combination of existing predictors and a new predictor best predicts the outcome.

**Conclusion** We advise investigators in incremental value studies to more explicitly consider using an approach that is in line with the research question they aim to answer, and to be aware that the approach influences the incremental value that can be identified.

**Table 1 (abstract O31).** Overview of the three approaches we distinguish to assess the incremental value of a new predictor and the incremental value of the D-dimer test for the prediction of deep venous thrombosis

Approach	Research question: “What is the incremental value of a new predictor...”	regression model with and without the new predictor	Modeling approach	Incremental value of D-dimer test (95%CI)	
1. Existing model	“...when the original model is used in the new dataset as originally developed?”	α + β_1_lp (a) vs. α + β_1_lp + β_2_NP (b)	Refitting all coefficients of the model is not an option. An alternative is to improve the discrimination and calibration of the original model in your dataset using fractional polynomials, splines (‘), or simple recalibration for the linear predictor (lp) of the original model. Note that α + β_1_ in model (a) may differ from α + β_1_ in model (b) due to adjustment for the new predictor.	ΔAUC NRIe NRIc	0.085 (-0.012 to 0.18) 0.084 (0.013 to 0.15) 0.64 (0.56 to 0.72)
2. Model revision	“...when the original model is optimally fit to the new dataset?”	α + β_1_X_1_ + β_2_X_2_ (a) vs. α + β_1_X_1_ + β_2_X_2_ + β_3_NP (b)	Refit entire model with the same predictors (a) and add the new predictor to a model with the same predictors as in the original model (b). Note that α + β_1,2,3_ in model (a) may differ from α + β_1,2,3_ in model (b) due to adjustment for the new predictor.	ΔAUC NRIe NRIc	0.082 (-0.012 to 0.18) 0.083 (0.0074 to 0.16) 0.61 (0.52 to 0.70)
3. New model development^*^	“...when the new predictor is incorporated in the original model in the new dataset?”	α + β_1_X_1_ + β_3_X_3_ (a) vs. α + β_1_X_1_ + β_4_NP (b) Here β_2_X_2_ was removed from the existing model (a). After adding the new predictor (b) β_3_X_3_ was replaced by β_4_NP	Refit entire model with the same predictors, but now allow predictor selection (a). Repeat this step, but now after adding the new predictor to the list of candidate predictors (b). Note that model (b) may include different predictors than model (a), due to replacement of predictors in model (a) by the new predictor in model (b)	ΔAUC NRIe NRIc	0.080 (-0.015 to 0.17) 0.077 (0.0023 to 0.15) 0.59 (0.48 to 0.70)

α = intercept as estimated in the new dataset; β_i_ = the association of predictor *i* with the outcome in the new dataset; lp = the linear predictor by applying the existing model to individuals in the new dataset; NP = the new predictor*;* AUC = Area Under the Receiver Operating Characteristic; NRIe = net reclassification improvement (NRI), event-based: risk categories: 0 – 15.9%, 15.9% – 100%; NRIc = net reclassification improvement (NRI), continuous

an “existing model” can be referred to as one that we don’t want to modify the coefficients of because it is so well established in clinical practice. With “model revision” and “new model development” we generally deal with a model that is open for modification and one that is not established in clinical practice and for which the coefficients or even the included predictors can be modified

### O32 The impact of outlier detection and removal on studies of biological variability (BV)

#### A. Sitch^1^, S. Mallett^1,2^, J. Deeks^1,2^

##### ^1^Test Evaluation Research Group, Institute of Applied Health Research, University of Birmingham, Birmingham, UK; ^2^National Institute for Health Research (NIHR) Birmingham Biomedical Research Centre

###### **Correspondence:** A. Sitch

**Background**

Biological variability (BV) studies measure the natural variability in test results occurring between and within individuals. BV estimates can guide appropriate use of tests for monitoring and diagnosis. Analysis of these studies routinely involves detecting and eliminating outliers. The risk of outlier removal inappropriately reducing estimates of variability is not known.

**Objectives**

To estimate the impact of commonly used methods to remove outliers in BV studies.

**Methods**

As biomarker data typically have a skewed distribution, measurements *y*_*ijk*_were simulated following a log-normal distribution according to the model ln(*y*_*ijk*_) = ln(*μ*) + ln(*α*_*i*_) + ln(*β*_*ij*_) + ln(*ε*_*ijk*_) where, $\ln \left({\alpha}_i\right)\sim N\left(0,{\sigma}_G^2\right)$, $\ln \left({\beta}_{ij}\right)\sim N\left(0,{\sigma}_I^2\right)$, $\ln \left({\varepsilon}_{ijk}\right)\sim N\left(0,{\sigma}_A^2\right)$ for patients *i* = 1, . . , 20, observations per patient *j* = 1, . . , 4 and assessments per observation *k* = 1, 2. Analytical, within-individual and between-individual standard deviations used were 0.5, 1 and 2 respectively. We randomly introduced outliers mimicking missed digit or laboratory errors, changing values by a factor of 10 or 2.

Outlier detection was performed using Cochran C test, Reed’s Criterion, Tukey IQR rule, Dixon’s Q test, Grubb’s test and ±3SD. 5,000 simulations were run and results compared with the simulation parameters.

**Results**

With outlier detection and removal used, in the absence of outliers, analytical, within-individual and between-individual variability are underestimated. Unnecessarily removal of measures varied between methods; median(Q1,Q3)[min,max] removed for 5,000 simulations using Cochran C test 2(0,4)[0,30] and Dixon’s Q test 0(0,0)[0,0]. Cochran C test and Tukey's IQR rule created the greatest bias ( − 10.6 × 10^−4^, −15.5 × 10^−4^ and −85.5 × 10^−4^ for analytical, within-individual and between-individual standard deviations respectively).

There were differences in the ability of outlier detection methods to detect real outliers dependent on the number present. Outliers correctly identified and removed ranged from a median of 0% to 100%.

**Conclusion**

Identification of outliers in BV studies should lead to data checking and correction where necessary. However, outlier detection methods should be used as sensitivity analyses as they may lead to underestimation of measures of variation.

### O33 Sample size for binary logistic prediction models: beyond events per variable criteria

#### M. van Smeden^1,2^, K. G. M. Moons^1^, J. A. H. de Groot^1^, G. S. Collins^3^, D. G. Altman^3^, M. J. C. Eijkemans^1^, J. B. Reitsma^1^

##### ^1^UMC Utrecht Julius Center, Utrecht University, Utrecht, the Netherlands; ^2^Clinical Epidemiology, Leiden University Medical Center, Leiden, the Netherlands; ^3^Centre for Statistics in Medicine, Botnar Research Centre, University of Oxford, United Kingdom

###### **Correspondence:** M. van Smeden

**Background**

Binary logistic regression is one of the most frequently applied statistical models for developing clinical prediction models. Developers of such models often rely on an Events Per Variable criterion (EPV), notably EPV ≥ 10, to determine the minimal sample size required and/or the maximum number of candidate predictors that can be examined.

**Objectives**

To improve upon the existing sample size guidance for binary logistic prediction models.

**Methods**

I present an extensive simulation study in which the influence of: EPV, events fraction, number of candidate predictors, the correlations and distributions of candidate predictor variables, area under the ROC curve, and predictor effects on out-of-sample predictive performance of prediction models were studied. The out-of-sample performance (calibration, discrimination and probability prediction error) of developed prediction models was evaluated before and after regression shrinkage and variable selection.

**Results**

The results indicate that EPV fails to have a strong relation with metrics of predictive performance, and is not an appropriate criterion for (binary) prediction model development studies. Out-of-sample predictive performance can better be approximated by considering the number of predictors, the total sample size and the events fraction.

**Conclusion**

Prediction modeling studies should not only consider EPV to determine sample size. Instead, new sample size criteria for prediction models should be developed that take into account: the number of candidate predictors, the total sample size and the events fraction. A simple-to-apply formula for such sample size calculations is presented.

### O34 Markers for targeted therapy: evaluation and implementation of a prognostic genomic test for individualized decision-making in breast cancer

#### Ewout W. Steyerberg^1,2^, Patrick M. Bossuyt^3^

##### ^1^Department of Biomedical Data Sciences, Leiden University Medical Center, Leiden, the Netherlands; ^2^Department of Public Health, Erasmus MC, Rotterdam, the Netherlands; ^3^Department of Clinical Epidemiology, AMC, Amsterdam, the Netherlands

**Background**

In precision medicine, new biomarkers and genomic tests may contribute to decision-making and improve outcomes by targeting therapy who benefit most. There is discussion about the evidence base required to make recommendations about their use. Do we need trials, observational studies, models or a combination?

**Objectives**

To explore how evidence on the prognostic strength of a genomic signature can contribute to individualized decision making on starting adjuvant chemotherapy for women with breast cancer.

**Methods**

The MINDACT trial was a randomized trial that enrolled 6,693 women with early-stage breast cancer. A 70-gene signature (Mammaprint) was used to estimate genomic risk, and clinical risk was estimated by a dichotomized version of the Adjuvant!Online risk calculator. 2,187 women with discordant risk results were randomly allocated to chemotherapy or no chemotherapy. We simulated the full risk distribution of these women and estimated individual benefit, assuming a constant relative effect of chemotherapy.

**Results**

The trial showed a prognostic effect of the signature (adjusted Hazard Ratio 2.4 for distant metastasis free survival, DMFS). Chemotherapy led to a 1.5% higher 5-year DMFS in 1,550 women (23%) with high clinical risk and low genomic risk. A decision-analytic modeling approach showed wide variability in DMFS within the high clinical risk group as defined in the trial, identified far fewer women who could benefit from genetic testing (4% rather than 50%), and fewer candidates for chemotherapy (3% rather than 27%). These proportions depended strongly on the required minimum benefit from chemotherapy and the anticipated relative effect of chemotherapy.

**Conclusions**: A high-quality pragmatic trial may be insufficient to directly inform clinical practice on the utility of a biomarker or genomic test for individual women. This study illustrates that more detailed risk estimation and further decision-analytic modeling may be required to support optimal clinical implementation.

### O35 ROC curves and classification plots for clinical prediction models: from waste of ink towards useful insight

#### J. Y. Verbakel^1,2^, E. W. Steyerberg^3^, H. Uno^4^, B. De Cock^1^, L. Wynants^1^, G. S. Collins^5^, B. Van Calster^1^

##### ^1^KU Leuven, Department of Development and Regeneration, Leuven, Belgium; ^2^Nuffield Department of Primary Care Health Sciences, University of Oxford, UK; ^3^Department of Medical Statistics and Bioinformatics, Leiden University Medical Centre (LUMC), Leiden, the Netherlands; ^4^Division of Population Sciences, Dana-Farber Cancer Institute, Boston (Massachusetts), USA; ^5^Centre for Statistics in Medicine, Nuffield Department of Orthopaedics, Rheumatology and Musculoskeletal Sciences, University of Oxford, Oxford, UK

###### **Correspondence:** J. Y. Verbakel

***Background:***

Receiver operating characteristic (ROC) curves are widely used in reports on clinical risk prediction models. Although the intent to demonstrate the ability of a model to discriminate between patients with and without a certain condition might be sincere, their presentation and interpretation is often inadequate.

***Objectives:***

ROC curves yield an improvement over selective reporting at identified optimal thresholds in early stage studies. However, we argue that most published ROC curves contain little useful information and are used erroneously to evaluate clinical prediction models. At the bare minimum classification thresholds should be displayed on the ROC plots.

***Methods:***

We encourage the use of classification plots, which plot sensitivity and specificity separately by threshold. Such classification plots can be supplemented with measures of clinical utility such as net benefit. We illustrate the usefulness of classification plots with a case study on residual mass diagnosis in metastatic testicular cancer patients.

***Results:***

ROC curves are common in the medical literature to evaluate the performance of clinical prediction models. Our pragmatic search revealed 62% of ROC curves were presented uninformatively. ROC curves provide little information over and above the area under the curve (AUC) as a summary of discriminatory ability when threshold information is not plotted.

***Conclusion:***

We recommend to focus on the AUC, sensitivity and specificity at clinically relevant thresholds, and, if a visualization of discriminatory ability is desired, classification plots where sensitivity and specificity is presented by threshold. Classification plots can be readily augmented with standardized net benefit to assess the potential clinical utility of a model.

**Fig. 1 (abstract O35).**
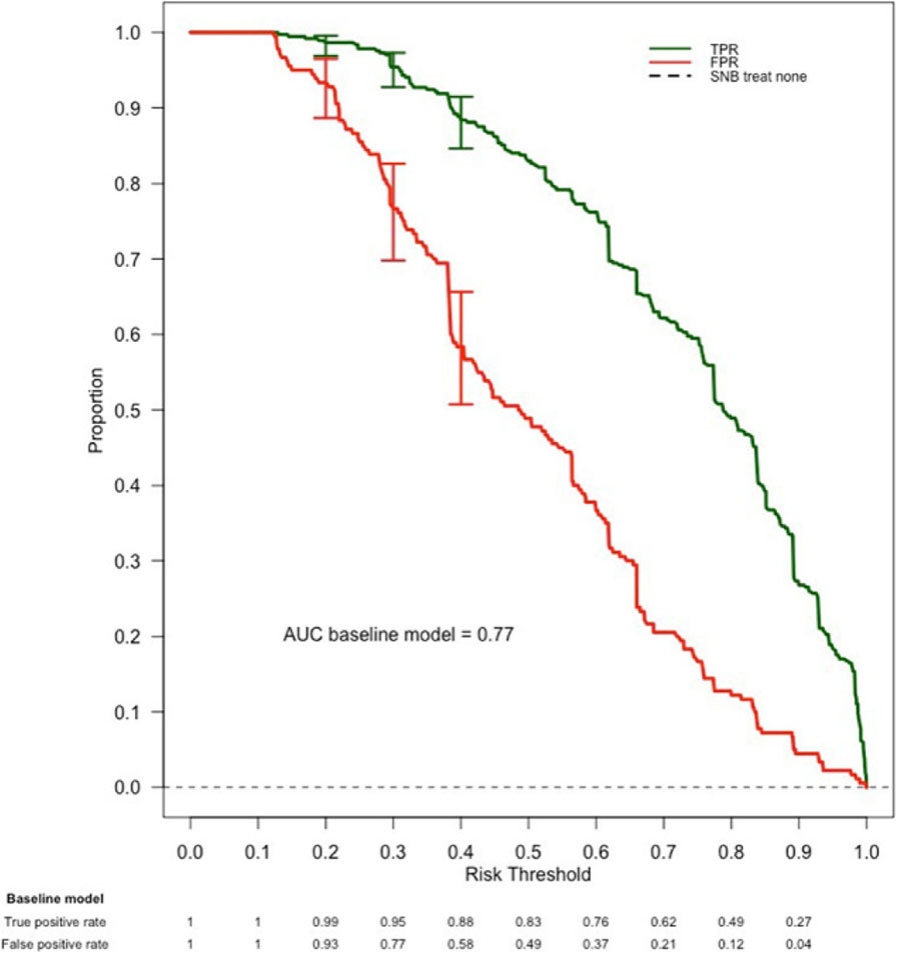
Classification plots showing sensitivity (true positive rate, TPR) and false positive rate (FPR) by threshold for the baseline model only (with 95% pointwise confidence intervals of TPR and FPR for risk thresholds of 20%, 30%, 40% for risk of metastatic remnants after chemotherapy in testicular cancer patients)

**Fig. 2 (abstract O35).**
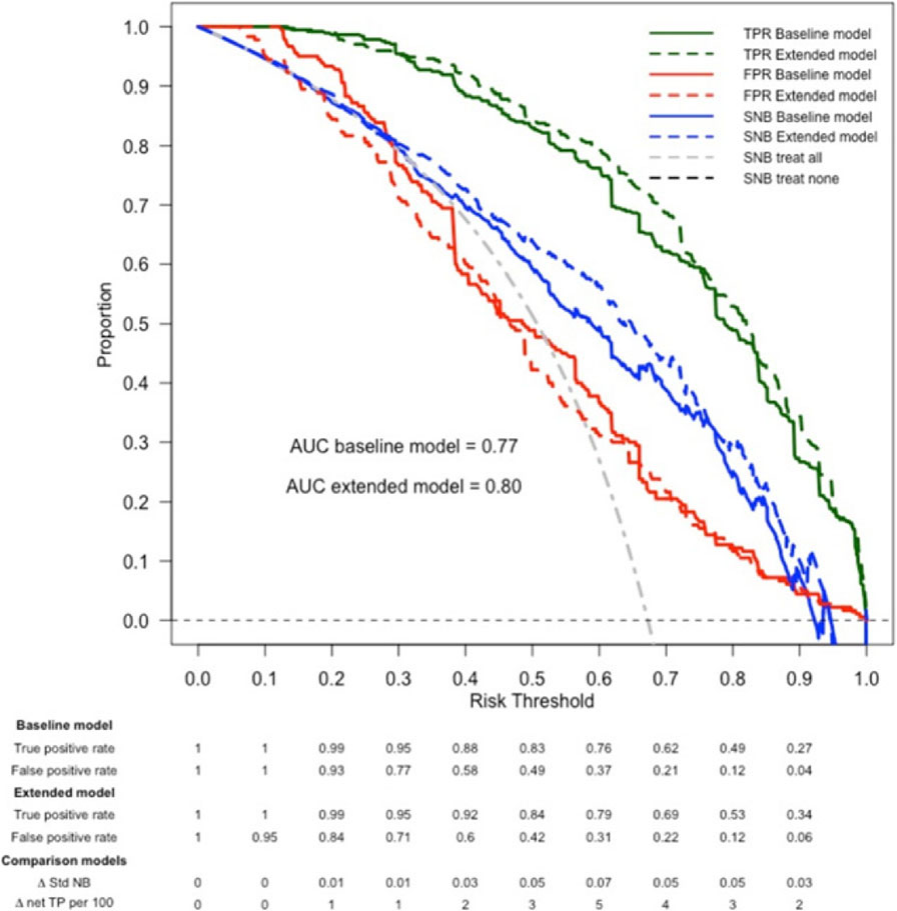
Classification plots including standardized decision curve for the baseline and extended model combined, to predict the risk of metastatic remnants after chemotherapy in testicular cancer patients

### O36 Understanding the effects of conditional dependence in research studies involving imperfect diagnostic tests

#### Zhuoyu Wang^1^, Nandini Dendukuri^1,2^, Lawrence Joseph^1,3^

##### ^1^Department of Epidemiology, Biostatistics and Occupational Health, McGill University; ^2^Technology Assessment Unit, McGill University Health Center; ^3^Division of Clinical Epidemiology, Department of Medicine, McGill University Health Center

###### **Correspondence:** Zhuoyu Wang

This abstract has been previously published.

Wang, Z., Dendukuri, N., and Joseph, L. (2017) Understanding the effects of conditional dependence in research studies involving imperfect diagnostic tests. Statist. Med., 36: 466–480. doi: 10.1002/sim.7148.

### O37 The effects of correlation between the test positive rate and prevalence on tailored meta-analysis

#### Brian H. Willis, Dyuti Coomar, Mohammed Baragilly

##### Institute of Applied Health Research, University of Birmingham

###### **Correspondence:** Brian H. Willis

**Background**

Meta-analysis may produce estimates that are unrepresentative of a test’s performance in practice. Tailored meta-analysis circumvents this by deriving an applicable region for the practice and selecting the studies compatible with the region. It requires the test positive rate, *r* and prevalence, *p* being estimated for the setting but previous studies have assumed their independence.

**Objective**

The aim is to investigate the effects a correlation between test positive rate and prevalence has on estimating the applicable region and how this affects tailored meta-analysis.

**Method**

Four methods for estimating 99% confidence intervals for *r* and *p* were investigated: Wilson’s score, Clopper-Pearson’s exact interval, the Bonferroni correction and Hotelling’s T^2^ statistic. These were analysed in terms of the coverage probability using simulation trials over different correlations, sample sizes, and values for *r* and *p*. The methods were then applied to two published meta-analyses with associated practice data and the effects on the applicable region, studies selected and summary estimates evaluated.

**Results**

Hotelling’s T^2^ statistic with a continuity correction had the highest median coverage (0.9971). This and the Clopper-Pearson method with a Bonferroni correction both had coverage consistently above 0.99. The coverage of Hotelling’s T^2^ statistic intervals varied the least across different correlations. For both meta-analyses, the number of studies selected was largest when Hotelling’s T^2^ statistic was used to derive the applicable region. In one instance this increased the sensitivity by over 4% compared with tailored meta-analysis estimates using other methods.

**Conclusion**

Tailored meta-analysis returns estimates which are tailored to practice providing the applicable region is accurately defined. This is most likely when the 99% confidence interval for test positive rate and prevalence are estimated using Hotelling’s T^2^ statistic with a continuity correction. Potentially, the applicable region may be obtained using routine electronic health data.

### O38 Evidence for reducing cancer specific mortality due to screening for breast cancer in Europe: a systematic review

#### Nadine Zielonke^1^, Andrea Gini^1^, Erik E. L. Jansen^1^, Ahti Anttila^2^, Nereo Segnan^3^, Carlo Senore^3^, Piret Veerus^4^, Harry J. de Koning^1^, Nicolien T. van Ravesteyn^1^ and Eveline A. M. Heijnsdijk^1^

##### ^1^Erasmus MC, Department of Public Health, Rotterdam, The Netherlands; ^2^Finnish Cancer Registry, Helsinki, Finland; ^3^Città della Salute e della Scienza University Hospital, Screening, Cancer Registry, Turin, Italy; ^4^National Institute for Health Development, Tallinn, Estonia

**Background:** Breast cancer screening in Europe has been debated heatedly in the last decades. The aim of this systematic review was to quantify the impact of organized screening on breast cancer mortality across European regions. To our knowledge, this review is the first that comprised all available evidence from different types of studies from all European regions and stringently used clearly defined tools to appraise the quality of each study and thus summarizes the best evidence.

**Methods:** Six databases were searched including Embase, Medline and Web of Science from inception to April 2016. To identify all eligible studies which showed the effect of organized screening on breast cancer mortality, two reviewers independently applied predefined inclusion and exclusion criteria regarding PICOS. Only original studies in English with a minimum of five years of follow-up that were either randomized controlled trials (RCTs) or observational studies were included. The Cochrane risk of bias instrument and the Newcastle-Ottawa Scale were used to assess the risk of bias of the included studies.

**Results:** Of the 3,336 references initially retrieved, 58 were included in the final analysis. Those comprised 37 cohort studies, 14 case-control studies and 7 randomized controlled trials. Surprisingly, none were from Eastern Europe. The quality of the included studies was very miscellaneous: only 14 were judged to be of very good or good quality. Of those, the reduction in breast cancer mortality varied from 4% to 54% in Northern Europe, 45% to 51% in Southern Europe and from 12% to 39% in Western Europe.

**Conclusion**: This systematic review provides evidence that organized screening reduces mortality from breast cancer in all European regions where screening was implemented and monitored, despite a wide range of estimates. Quantification of the actual effects of screening in terms of benefits is still lacking for Eastern Europe.

